# A Proposed Framework for Ranking and Prioritizing Food Safety Risks in Low Resource Settings Using Foodborne Disease Burden Metrics: A Case Study in Ethiopia

**DOI:** 10.1016/j.jfp.2025.100525

**Published:** 2025-06-23

**Authors:** Barbara Kowalcyk, Leon Gorris, Janet Buffer, Kathryn Stolte-Carroll, Bashiru C. Bakin, Allison Howell, Desalegne Degefaw, Binyam Moges, Kara Morgan, Laura Binkley, Getnet Yimer, Arie H. Havelaar

**Affiliations:** 1 Center for Foodborne Illness Research and Prevention, The Ohio State University, Columbus, Ohio, USA; 2 Translational Data Analytics Institute, The Ohio State University, Columbus, Ohio, USA; 3 Milken Institute School of Public Health, George Washington University, Washington, District of Columbia, USA; 4 Food Safety Futures, Nijmegen, the Netherlands; 5 Global One Health Initiative, The Ohio State University, Columbus, Ohio, USA; 6 Department of Genetics and Penn Center for Global Genomics & Health Equity, Perelman School of Medicine, University of Pennsylvania, Philadelphia, Pennsylvania, USA; 7 Emerging Pathogens Institute, Global Food Systems Institute, Animal Sciences Department, University of Florida, Gainesville, Florida, USA

**Keywords:** Decision-making, Risk analysis, Risk prioritization, Risk ranking, Risk-based food safety management systems

## Abstract

Risk-based food safety systems are recognized as the best way to address food safety in an effective and efficient manner. Central to risk-based food safety systems is an assessment, ranking, and prioritization of risks associated with foodborne hazards that informs objective, evidence-based decisions on risk mitigation in a systematic and transparent manner. While the importance of such approaches is well recognized, many governments struggle with where to begin. We adapted and implemented a framework for risk-based decision-making in low-resource settings using Ethiopia as an example, engaging Ethiopian stakeholders in a multiphase process to identify food safety priorities. First, stakeholder representatives were engaged to define statements of concern and purpose; identify foodborne hazards of public health relevance in Ethiopia; and select appropriate risk metrics for risk ranking and prioritization. Second, estimates for each risk metric were calculated for selected foodborne hazards using World Health Organization (WHO) data and expert elicitation. Third, stakeholder representatives were engaged in ranking the hazards as High, Medium, or Low risk; notably, mortality was the preferred risk metric in decision-making. Fourth, attribution estimates to food groups were developed for hazards ranked as High risk using expert elicitation to supplement WHO data. Lastly, stakeholder representatives were engaged in a risk prioritization exercise that identified key control points in four food supply chains and quantified their impact on risk through mitigation of hazards or prevention of contamination. Our proposed framework provides a systematic, evidence-based, and adaptable method for prioritizing the allocation of public sector resources. The process can generate the evidence needed to strengthen regulatory systems and support the efficient implementation of national-level food safety strategies that span from farm to fork to improve public health.

## Highlights

• Scope of FBD risk ranking/prioritization efforts should be defined by stakeholders.
• WHO FBD data combined with expert elicitation support risk ranking.
• FBD burden risk ranking in Ethiopia was driven by mortality.
• FBD burden risk prioritization in Ethiopia was informed by risk ranking.
• Close coordination and collaboration are needed within and between stakeholder groups.

Each year, an estimated 600 million people are sickened, and 420,000 die from foodborne disease (FBD), resulting in the loss of more the 33 million healthy life years (Havelaar et al., 2015). Low- and middle-income countries (LMICs) bear a disproportionate amount of this burden at a significant cost to human health, trade, and development (Grace, 2015). For example, the World Bank estimates that, each year, unsafe food costs LMICs $15 billion in medical costs and $95 billion in productivity losses (Jaffee et al., 2019). Despite the staggering impact of foodborne illness in LMICs, most food safety resources have typically been dedicated to ensuring the safety of food exports (Unnevehr & Ronchi, 2014). There is increasing awareness that domestic food safety systems need to be strengthened but many LMICs have not developed priorities for investment (Jaffee et al., 2019).

To ensure that food is safe for consumption, it is essential to identify, assess, and control all relevant hazards, with a focus on those hazards that have the largest impact on public health. Such systems are rooted in risk analysis and include processes for identifying food safety hazards and associated risks, estimating the level of public health impact of those risks (i.e., risk assessment), ranking the risks based on their level of public health impact, identifying risk management options to prevent or mitigate risks, selecting decision criteria, gathering evidence, and setting priorities for managing the risks (Food and Agriculture Organization of the United Nations (FAO), 2006, FAO, 2017, FAO, 2020, CAC, 2007, OIE, 2010). Importantly, risk-based decision-making utilizes data and information on the likelihood and severity of harm (i.e., risk), including characterization of uncertainty in these risk estimates, to rank risks and prioritize the allocation of resources (Morgan et al., 2021). Lack of a systematic, risk-based approach to facilitate decision-making can cause problems ranging from a decrease in public trust to the occurrence of unintended consequences to society, the environment, and the marketplace (Food and Agriculture Organization of the United Nations (FAO), 2006, IM-NRC, 2010).

Risk ranking and prioritization help identify hazards that pose the greatest risk to public health and support the development and implementation of control measures with the greatest potential to prevent or mitigate harm to human health (Koutsoumanis & Aspridou, 2016). The process should begin with risk ranking which systematically identifies, analyzes, sorts, and orders potential hazards that may occur at different stages of the food chain (i.e., from production to consumption) using estimates of the likelihood and severity of adverse impacts on human health (FAO, 2020). Risk prioritization then sets priorities for managing the ranked risks, which is often a complex task that involves making trade-offs (IM-NRC, 2010, Ruzante et al., 2017).

Many food safety risk ranking and prioritization efforts have been conducted worldwide using a range of quantitative and qualitative methods (Batz et al., 2012, Chen et al., 2013, Lake et al., 2010, van Asselt et al., 2013, Van der Fels-Klerx et al., 2018). While a universal methodology for risk ranking is lacking (European Food Safety Authority (EFSA) Panel on Biological Hazards (BIOHAZ), 2012), efforts have been made to provide guidance and to facilitate governments and others in adopting risk ranking in the food safety area and prioritizing allocation of resources to most efficiently minimize foodborne illness risks (FAO, 2020). Qualitative approaches typically involve the use of risk summaries, risk matrices, decision trees, or risk rules, such as a likelihood-severity grid (Chen et al., 2013). While such approaches may be more efficient in terms of time and resources, they, by necessity, rely on many unspecified and/or unvalidated assumptions and subjective categorizations, and final risk estimates, therefore, may not accurately reflect the true risk (FAO, 2020). Quantitative approaches are more objective, data-driven, and reproducible than qualitative methods but require a significant amount of data and resources and may not be feasible for all hazards (FAO, 2020). Regardless of the approach used, it is crucial that risk ranking and prioritization efforts use reliable methods and data to ensure that results accurately reflect risk (Lindqvist et al., 2020). While the most appropriate method for ranking may vary, the approach should be structured, transparent, and driven by a clearly stated purpose, availability of data, and available resources (EFSA-BIOHAZ, 2012).

Recognizing the integral role of risk ranking and prioritization in ensuring food safety, several intergovernmental agencies, including the European Food Safety Authority and FAO, have published technical reports on risk ranking and prioritization methods (Food and Agriculture Organization of the United Nations (FAO), 2006, FAO, 2017, FAO, 2020, EFSA-BIOHAZ, 2012, European Food Safety Authority (EFSA) Panel on Biological Hazards (BIOHAZ), 2015). These publications tend to focus their discussions on the mathematical and methodological approaches for generating risk estimates, ranking the risks, and selecting priorities. Few describe the work that is done ***before*** such approaches can be undertaken. In 2020, FAO released a risk ranking guidance document to provide direction to national food safety authorities and stakeholders on how to rank foodborne hazards in terms of the food safety risks they pose in their countries based on their impact on public health (FAO, 2020). The guidance document emphasizes the importance of including stakeholders throughout the risk ranking process, including defining the scope, developing the approach, and conducting the risk ranking. However, the approach has not been tested and did not address the best approach for risk prioritization and engagement of stakeholders/experts therein.

Given the global importance of preventing FBD, there is a need for additional guidance on the implementation of risk-based approaches in low-resource settings, such as those encountered in LMICs as well as areas where limited data are available. Many countries, including Ethiopia, have limited resources for food safety, and there is a lack of coordination and connectivity between the agencies that work on food safety (Ayalew et al., 2013). Even if stakeholders (e.g., government agencies, academic institutions, food producers, consumers) are poised to start addressing food safety, working without a shared set of priorities will result in siloed and fragmented efforts that are inefficient and, too often, ineffective in advancing public health goals. Multistakeholder dialogues informed by evidence-based analysis are critical to developing a shared vision for transforming food systems, providing legitimacy to the process, and increasing the likelihood of sustainable change (UNEP et al., 2023). Therefore, the primary goal of this work was to implement and expand the FAO risk ranking guidance document to develop a systematic and transparent framework for ranking and prioritizing food safety risks in low-resource settings, such as LMICs, using Ethiopia as an example.

## Materials and methods

**Figure 1:**
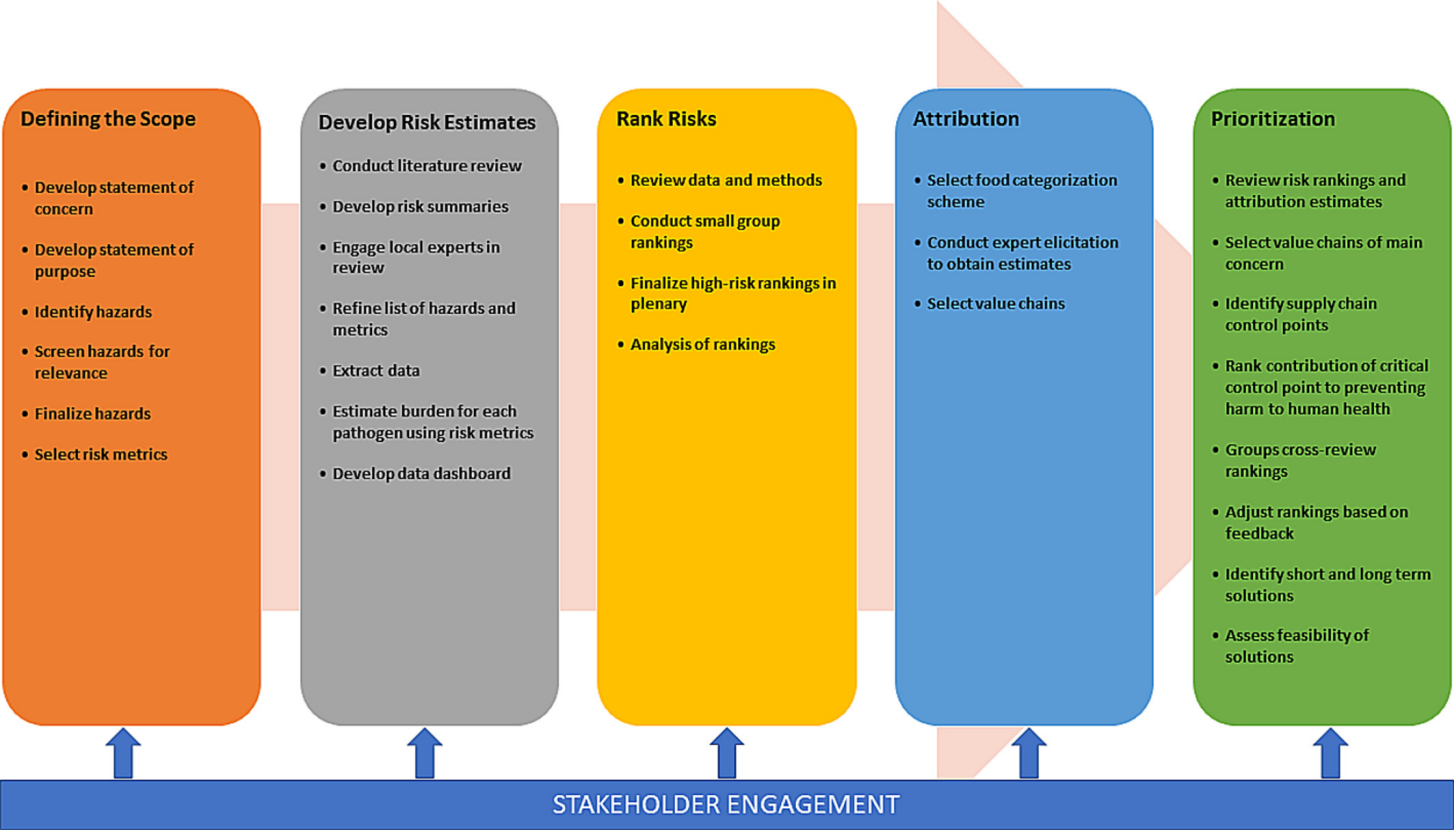
Overview of hazard identification, risk ranking, and intervention prioritization activities undertaken in this study.

A stakeholder-driven, multiphase approach was used to develop a shared list of risk ranking and prioritization activities for food safety in Ethiopia (Fig. 1). Ethiopia was selected because (1) our team had strong in-country collaborators, and (2) there was evidence that Ethiopian stakeholders were ready to engage in the proposed activities. As described below, a workshop was held to define the scope of the risk ranking and prioritization effort, in terms of most relevant hazards, which was informed by a situation analysis conducted by the study team. The public health risk of the selected hazards was estimated using World Health Organization (WHO) data, published literature, and expert elicitation and, subsequently, used in a second workshop to rank the hazards. The burden of high-priority hazards was attributed to food groups using existing data and expert elicitation. A third workshop was held to identify and prioritize potential strategies for mitigating the risk of contamination in the food groups with the highest attributable burden. Government agencies, academic institutions, and other nongovernmental organizations with a role in food safety (i.e., stakeholders) were identified and invited to send a representative to participate in the workshops. A highly skilled and professional facilitator was engaged in all workshops to ensure that best practices in group decision-making and stakeholder engagement were employed. Workshop agendas with participating organizations and a background document provided to stakeholders are presented in Appendices A and B, respectively.

**Defining the scope.** A 4-day Scoping workshop was held in March 2020 in Addis Ababa, Ethiopia, to engage stakeholders in defining the scope of the risk ranking and prioritization. Relevant government agencies and academic, nongovernmental, and private sector organizations were identified, and participants were recruited from each organization. Outputs from the workshop included a Statement of Concern, a Statement of Purpose, the hazards to be ranked, and the preferred risk metrics for conducting the ranking.

Government agency decision-makers were engaged on Days 1 and 4 with technical leads from each agency (i.e., risk managers) working in subgroups on technical issues on Days 2 and 3. This approach was designed to achieve maximum engagement with the risk managers who would use the risk ranking for decision-making but also provide a small enough group to facilitate a high level of interaction on the technical issues. Each session lasted approximately four hours. Participants were asked to complete evaluations at the end of each session, and the results were used to inform planning for the following session.

Draft documents for the Statement of Concern and Statement of Purpose were developed by the project team in advance based on initial feedback from Ethiopian partners, and participants modified the language until it accurately represented their needs. Participants were then provided an overview of the global burden of disease estimates produced by the WHO Foodborne Disease Burden Epidemiology Reference Group (FERG), as well as an explanation of which hazards were included in these estimates, and what data were used to develop estimates. It was noted that the selection of hazards presented on the FERG list was based on global levels of concern and global data availability, rather than on concerns and data specific to Ethiopia. Participants were asked to identify, in subgroups, additional hazards that might be included in the subsequent risk ranking and prioritization effort.

Once the complete list of potential hazards was developed, participants screened the additional hazards for relevance in Ethiopia. There was no set target number of hazards but, given limited resources, it was important to narrow the list of hazards to those most relevant for Ethiopia. First, each hazard was reviewed to determine if it could be transmitted by food and, if not, the hazard was excluded. Participants then worked in subgroups to identify (1) the reason for including the hazard and (2) the government agencies currently working to mitigate the hazard in the food supply. The full list of hazards was discussed in a plenary session and participants voted on the hazards to include or not in the risk ranking and prioritization.

Finally, participants were engaged in selecting preferred risk metrics for the risk ranking. Participants were provided with an overview of the metrics used in the FERG estimates, including incidence, mortality, Years Lived with Disability (YLD), Years of Life Lost (YLL), and Disability Adjusted Life Years (DALYS). The technical basis of these metrics was presented, and participants walked through various metrics to explain underlying concepts and address their questions. The final list of risk metrics was selected by the participants, who voted iteratively until there was consensus around the priority metrics to be used in the risk ranking.

**Estimating disease burden.** Disease burden estimates for a range of foodborne hazards have been published (Havelaar et al., 2015), and a selection of these were used to calculate estimates for each hazard and risk metric selected in the Scoping workshop. While the global and regional estimates have been published in a range of research papers, country-specific estimates, while available, have been largely unpublished. The project team sought and obtained permission from the Ethiopian government to publish country-specific estimates for the FERG hazards (Havelaar et al., 2022). Estimates for metals (arsenic, cadmium, lead, methylmercury) were based on Gibb et al. (2019).

Mean estimates and 95% uncertainty intervals were calculated for each of the nine selected risk metrics for hazards not considered by FERG (i.e., non-FERG hazards). When available, data were extracted from published literature. In the absence of published data, assumptions were made, reviewed with Ethiopian project team members and, when appropriate, adjusted. A standardized method was used to estimate uncertainty for each input (Appendix C). Since the FERG estimates (Havelaar et al., 2015) are the most recent available global estimates and were based on 2010 data, 2010 was also used as the reference year for all burden estimates and the population size of Ethiopia was assumed to be 87.64 million (Global Burden of Disease (GBD), 2019).

A data dashboard was created using the R package *shiny* (Chang et al., 2023) to visualize disease burden estimates for all hazards and to simulate burden estimates for non-FERG hazards. The dashboard provides multiple ways to view (i.e., box plots, scatter plots) and dynamically compare burden estimates between groups of hazards, side by side. The plots are generated using the R *ggplot2* package (Wickham, 2016). In addition, the dashboard features a simulation that can generate burden estimates for custom hazards, even with a minimal amount of published data by defining uncertainty bounds. Complete details can be found in Appendix C.

**Ranking the risks.** A 2.5-day Ranking workshop was held in June 2022 in Addis Ababa, Ethiopia, to engage stakeholders in ranking the foodborne hazards selected during the Scoping workshop based on their public health burden in Ethiopia. Outputs included a list of hazards ranked as High, Medium, or Low risk. Participants were invited from the same organizations that attended the Scoping workshop. A background document, including an overview of the hazards to be ranked, burden estimates associated with each hazard, and methods used to calculate burden estimates, was shared with all participants prior to the workshop (Appendix B).

At the workshop, participants were refamiliarized with the hazards and risk metrics selected in the Scoping workshop. Ethiopian estimates for the hazards included in the FERG analysis were presented, and participants were invited to walk around the meeting room to review posters presenting the burden associated with individual hazards. Following the poster review, participants were broken into subgroups and rotated through a set of presentations (i.e., a round robin approach) on the methods used to estimate the burden of risk associated with each of the non-FERG hazards. This process allowed participants to raise questions and discuss the information provided in each poster in small groups, increasing their understanding of the burden estimate outcomes for various hazards to be ranked.

Hazards were ranked using an iterative approach. Participants were randomly assigned to four groups. Each group ranked the hazards as either High, Medium, or Low risk in two rounds. In Round 1, the groups were randomly assigned one of the risk metrics selected in the Scoping workshop and ranked various hazards according to their assigned metric. In Round 2, the same groups were invited to consider additional metrics of their choice and revise their initial rankings. A hazard ranking was considered final if all groups gave the hazard the same ranking at the end of Round 1 or Round 2. Hazards without that consensus were discussed in a plenary by the entire group, but only if the hazard had been ranked High risk by at least one group. No effort was made to achieve consensus for hazards rated differently by groups as Low or Medium risk, since the subsequent prioritization was planned for high-risk hazards only. Categorization of these latter hazards was debated, with each subgroup explaining why they ranked the hazard the way they did. Following the discussion, each individual ranked the hazards. In cases where there was still no consensus, participants circulated in small groups to further discuss the remaining hazards before returning to plenary. Remaining discrepancies were discussed in plenary and a vote was taken for the final categorization. After the ranking was finalized, participants were asked to state their level of agreement on the outcome of identifying high-risk hazards.

**Estimating attribution.** To develop a roadmap for building a stronger food safety system in Ethiopia, it is important to consider the foods that contribute the most to transmission of the hazards identified as High risk in the Ranking workshop, and to assess how transmission might best be controlled. To inform such prioritization activities, a complete and consistent set of attribution estimates was needed, which is currently lacking for Ethiopia. Therefore, attribution estimates were developed using a Delphi process (Dalkey and Helmer, 1963), in two rounds, to anonymously elicit information from a panel of workshop participants to be involved in the Prioritization workshop (Appendix C). Briefly, experts were provided with data on the Ethiopian burden of the hazards ranked High in the Ranking workshop (Appendix C) and asked to attribute the burden to food groups that were used in a previously published analysis conducted by FERG (Hoffmann et al., 2017). Food groups in the previously published work that are rarely consumed in Ethiopia (e.g., pork, seaweed) were not included in the attribution exercise. Pathogens transmitted through only one food group (e.g., *Mycobacterium bovis* can only be transmitted through contaminated dairy products) were also not considered by the expert panel. Attribution estimates from the Ethiopian experts were combined with estimates developed by the study team using literature and additional assumptions (Appendix C). Mortality was the main metric considered by stakeholders in the risk ranking, so estimates focused on the attribution of FBD deaths, which was assumed to be proportional to the attribution of FBD cases.

**Prioritization.** A 3-day Prioritization workshop was held in June 2023 to engage stakeholders in (1) prioritizing control options for the high-risk hazards identified in the Ranking workshop in the most relevant food groups; and (2) developing short- and long-term goals for implementation of control options. Outputs included concrete recommendations on food supply chain interventions with expected high impact on public health and for improving coordination and collaboration between various agencies with the potential to advance a stronger food safety system in Ethiopia. Participants were invited from the same organizations that attended the Scoping and Ranking workshops.

The workshop began with an overview of the outcomes of the Scoping and Ranking workshops, followed by a presentation of the attribution estimates for foodborne deaths. Participants used the analysis of attributable deaths to select four food value chains to be considered in the prioritization efforts. Participants were assigned to groups that focused on a single food value chain. Each group developed a diagram of their assigned food group and identified supply chain control points (SCCPs) where a measure, activity, or action could be taken to prevent, control, reduce, or eliminate contamination by a hazard or a group of hazards and minimize the contribution of the food group to the burden of FBD.

Participants were then asked to weigh the relative impact of each SCCP on preventing deaths due to each hazard category. For this purpose, the high-risk hazards were grouped into three categories with similar ecology: (1) anthroponotic pathogens, defined as pathogens that exclusively, or predominantly have human reservoirs (enteropathogenic *Escherichia coli* (EPEC), enterotoxigenic *Escherichia coli* (ETEC), norovirus, rotavirus, *Salmonella* Typhi, *Shigella* spp, *Vibrio cholerae*); (2) zoonotic pathogens, defined as pathogens that exclusively, or predominantly have animal reservoirs (*Campylobacter* spp, *Mycobacterium bovis*, Nontyphoidal *Salmonella enterica*), and (3) chemicals (aflatoxin B1, arsenic). Participants then weighted the SCCPs in their food group in terms of their contribution to preventing contamination for each hazard category in a group discussion. The number of preventable deaths for each SCCP was then calculated as the proportion of points assigned to that SCCP, multiplied by the number of attributable deaths per hazard category for each food chain separately. Sunburst diagrams were constructed to display the levels of hierarchy (point in value chain, SCCP, hazard category) in each food value chain and their proportional impact on FBD deaths (Appendix D).

Short- and long-term solutions for controlling hazards at the highest-ranked SCCPs in each food value chain were identified by each group using a fishbone approach that is also used for root cause analysis (Kane et al., 2024). For each considered SCCP, the groups asked themselves why the SCCP could fail, the effect of the failure, and how to prevent a failure. A fishbone diagram was used to document the root causes of SCCP failures and develop potential risk mitigation strategies. The groups then classified the strategies as short-term, defined to be a strategy that could be implemented in three to five years, or long-term, defined to be a strategy that could be implemented in eight to ten years. Groups then presented their strategies in a plenary session and discussed commonalities.

Additional exercises were conducted to help facilitate stakeholder ownership of advancing identified priorities. First, a fishbowl exercise (Munn & Henderson, 2023) was conducted to discuss existing coordination mechanisms, what is working well and what is not yet working optimally, at the national and local levels to enable a strong food safety system in Ethiopia. Second, a “What I Need From You” (WINFY) exercise (Liberating Structures, n.d.) was conducted to identify what stakeholder groups need from each other to advance the identified priorities. Finally, participants were asked to identify one concrete task or assignment they would take away from the workshop. Evaluations were also collected at the end of the workshop.

**Data analysis.** All data extraction, manipulation, plots, and statistical testing were generated as outlined in Appendix C. In brief, an approximate analytical approach was used to calculate best estimates and uncertainty intervals for each risk metric (e.g., incidence, mortality, DALYs) for each pathogen using country-specific data from WHO FERG for Ethiopia, published literature and, in some cases, assumptions that were reviewed and adjusted by Ethiopian stakeholders. A data dashboard was developed to present results to stakeholders. Descriptive statistics were used to summarize risk ranking results, and ordinal logistic regression was used to identify predictors of the final ranking. A Delphi process was used to attribute foodborne deaths to hazards and food groups. Prioritization results were summarized using sunburst plots. All analyses were conducted in R and/or EXCEL.

## Results

**Defining the scope.** A total of 37 participants from 12 organizations, including 16 from government agencies, attended the Scoping workshop. The finalized statements of concern and purpose are presented in Table 1. Workshop participants reviewed the hazards included in the FERG global and regional burden of disease estimates (Havelaar et al., 2015, Gibb et al., 2019) for relevance in Ethiopia, and nominated eight additional hazards for inclusion in the risk ranking. Of these, *Coxiella burnetii* was eliminated because there is no existing evidence that it can be transmitted through food (EFSA Panel on Animal Health and Welfare (AHAW), 2010). *Aspergillus flavus* was eliminated because the agent is not a hazard itself but rather produces a hazardous toxin (aflatoxin) that was already included on the FERG list. During the review process, three additional hazards were identified by one of the subgroups and the larger group agreed that they should be included. The final list included 24 FERG hazards, four metals, and nine additional hazards, for a total of 37 hazards (Table 2).

**Table 1:** Statement of concern and statement of purpose developed with the Ethiopia stakeholders

**Statement of Concern** According to the World Health Organization, foodborne disease is an important public health problem in Africa. Many stakeholders in Ethiopia are poised to start addressing various foodborne risks posing varying levels of risk to consumers. However, many food safety stakeholders in Ethiopia are working independently without a shared set of priorities, which will have less impact in preventing and reducing the public health impact (e.g., mortality, morbidity, disability).
**Statement of Purpose** The purpose of this risk ranking is to identify the hazards that are relatively higher priorities for Ethiopia in terms of their contribution to the overall public health burden (e.g., mortality, disability, morbidity) of foodborne disease. Ranked risks and a final prioritized set of food hazards can inform stakeholders working on food safety in Ethiopia. These tools can leverage their collaborative effort and available resources in a systems approach for a greater impact in risk reduction.

**Table 2:** List of hazards initially identified by Ethiopian stakeholders during the Scoping workshop to be included in the risk ranking in addition to global hazards

Hazard	Justification from Stakeholders
**Viruses**	
Rift Valley Fever virus	Threat to public health and economy due to presence in neighboring countries. Government priority. Impact on trade. Ethiopia provides favorable climate for RVF.
Rotavirus	Common cause of persistent diarrhea in children that can lead to chronic malnutrition and metabolite imbalance. The most common cause of acute gastroenteritis in children (major cause of child mortality and morbidity).
**Bacterial toxins**	
*Clostridium botulinum*	Very deadly toxin occurring in canned foods and honey. Ethiopia is moving toward industrialization of food processing. Outbreaks have occurred. Outcome is fatal.
*Staphylococcus aureus*	Common cause of foodborne disease including severe diarrhea, especially in the immunocompromised. Widespread exposure to food handlers. Toxin causes food poisoning.
**Invasive infectious disease agents**	
*Bacillus anthracis*	Causes severe morbidity and mortality. Common, especially in rural communities. Raw meat consumption is high. Prevalent in animals, spore-forming, contagious, and zoonotic.
**Helminths**	
*Taenia saginata*	Very common due to raw meat consumption. Morbidity and mortality are high, especially in children. Causes weight loss, discomfort, and constipation, competes with nutrients.
**Natural toxins**	
Aflatoxin M1	Common in milk, which most people use. Morbidity is very serious and mortality is high. More severe in children.
Aflatoxin G1	Exists in staple diet. Morbidity is very serious, and mortality is high.
Other Aflatoxins	Prevalent in shiro, berbere, cereals, and other spices. Causes hepatocellular carcinoma (severe).
Ochratoxin	Commonly found in coffee. As a major coffee producer and frequent consumers of coffee, it has both health and economic impacts, including morbidity and mortality, in Ethiopia.
*Lathyrus sativus*	Disability is high in areas where it is grown.
**Processing contaminants**	
Acrylamide	Carcinogen when cooking foods at high temperatures.
**Adulterants**	
Formalin	Common chemical used as milk preservative by farmers. Adverse health effects.
Urea	Used widely by farmers to preserve milk.
**Agro-chemicals** DDT Aluminum phosphate Malathion	Widely used by farmers for treating crops: khat, vegetables, legumes, cereals, and fruits for sale. Can be fatal (morbidity and mortality are high). Organophosphate poisoning is a major concern for rural population considering they do not rinse fresh produce before consumption. Results in economic loss.
**Drug/antibiotic residues** Tetracycline Trypanocidal agents	Common for farmers to use on animals. Causes allergy and toxicity in humans along with long-term effects such as cancer, AMR, hypersensitivities, adverse reactions, and can be teratogenic. High economic impact: Stock loss due to disease, export income loss due to rejection.

Participants identified five risk metrics that are most meaningful to Ethiopian decision-makers: (1) incidence rate; (2) mortality rate; (3) YLL rate; (4) DALY rate; and (5) case fatality ratio. YLL is highly correlated with other risk metrics, particularly mortality rate, and was ultimately removed as a risk metric.

**Estimating burden.** Best estimates and 95% confidence intervals for the four selected risk metrics are presented by hazard group in Table 3. *Campylobacter* spp. had the highest estimated incidence rate followed by norovirus, rotavirus, ETEC, and nontyphoidal *Salmonella enterica*. *Vibrio* spp. had the highest mortality rate followed by EPEC, nontyphoidal *Salmonella enterica*, ETEC, and norovirus. Aflatoxin B1 had the highest case fatality ratio, followed by arsenic, cadmium, *Listeria monocytogenes*, and *Mycobacterium bovis*. *Vibrio* spp. had the highest DALY rate, followed by EPEC, nontyphoidal *Salmonella enterica*, ETEC, and lead.

**Table 3:** Best estimates (95% uncertainty intervals) for disease burden estimates for Ethiopia by hazard^a^

Hazard Type	Pathogen	Incidence rate (per 100,000)	Mortality rate (per 100,000)	Case-fatality ratio (%)	DALYs rate (per 100,000)
FERG Microbiological Hazards	Aflatoxin B1	0.019 (1.9e−4, 0.094)	0.013 (1.7e−4, 0.085)	90.19 (90.19, 90.19)	0.46 (0.0062, 3)
	*Ascaris* spp*.*	92 (17, 164)	0.0093 (1.3e−5, 0.01)	0.011 (3.7e−5, 0.10)	6 (1, 14)
	*Brucella* spp.	1 (0.013, 63)	0.65 (6.5e−5, 2)	0.50 (0.34, 0.66)	0.36 (0.004, 19)
	*Campylobacter* spp*.*	2152 (309, 8391)	0.75 (0.35, 1.26)	0.034 (0.0089, 0.23)	69 (32, 115)
	*Cryptosporidium* spp.	186 (0, 909)	0.15 (0, 0.53)	0.075 (0.024, 0.28)	12 (0, 44)
	Dioxins	0.092 (0.003, 9.30)	0 (0, 0)	0 (0, 0)	0.11 (0.0033, 11)
	*Echinococcus granulosus*	2 (0.5, 3)	0.017 (0.0036, 0.054)	1.09 (0.40, 2.51)	1.43 (0.036, 4)
	*Entamoeba histolytica*	742 (0, 4288)	0.05 (0, 0.44)	0.0068 (0.001, 0.084)	5 (0, 40)
	Enteropathogenic *E. coli* (EPEC)	430 (17, 1331)	1.67 (0.073, 3.98)	0.38 (0.16, 0.99)	136 (6, 321)
	Enterotoxigenic *E. coli* (ETEC)	939 (141, 2694)	1.27 (0.21, 2.86)	0.13 (0.054, 0.38)	103 (17, 236)
	*Fasciola* spp*.*	0.0052 (0.0018, 0.015)	0 (0, 0)	0 (0, 0)	0.038 (0.013, 0.1)
	*Giardia* spp*.*	663 (0, 3060)	0 (0, 0)	0 (0, 0)	0.68 (0, 3)
	Hepatitis A	337 (45, 1117)	0.671 (0.097, 2.18)	0.20 (0.051, 0.79)	33 (5, 103)
	*Listeria monocytogenes*	0.14 (2.5e−5, 2.5)	0.03 (5.6e−6, 0.055)	22.39 (19.24, 25.77)	1 (2e−4, 21)
	*Mycobacterium bovis*	7 (4, 10)	0.37 (0.22, 0.54)	5.48 (3.63, 8.21)	22 (13, 32)
	Nontyphoidal *S. enterica*	875 (100, 3159)	1.55 (0.34, 2.65)	0.17 (0.053, 0.77)	116 (25, 201)
	Norovirus	1609 (0, 5882)	1 (0, 3)	0.063 (0.026, 0.14)	76 (0, 226)
	*Salmonella* Paratyphi A	24 (0, 87)	0.157 (0, 0.56)	0.65 (0.65, 0.65)	11 (0, 40)
	*Salmonella* Typhi	106 (0, 378)	0.68 (0, 2.44)	0.65 (0.65, 0.65)	49 (0, 174)
	Shiga toxin-producing *E. coli* (STEC)	0.5 (0.07, 2)	5.7e−5 (5.2e−6, 2.4e−4)	0.011 (0.0045, 0.024)	0.0048 (0.0006, 0.02)
	*Shigella* spp.	435 (0, 3296)	0.45 (0, 1.79)	0.093 (0.024, 0.72)	37 (0, 147)
	*Toxoplasma gondii*	353 (163, 617)	0.023 (0.0082, 0.05)	0.0065 (0.0033, 0.012)	28 (13, 51)
	*Trichinella* spp.	6.6e−4 (2.2e−4, 0.001)	2.4e−5 (7.8e−6, 4e−5)	3.57 (3.57, 3.57)	0.0014 (4.7e−4, 0.0024)
	*Vibrio* spp.	72 (2, 207)	2.72 (0.075, 7.30)	3.80 (2.61, 5.01)	190 (5, 511)
FERG Chemical Hazards	Arsenic	2.39 (0.51, 4.31)	0.65 (0.14, 1.67)	27.12 (27.12, 27.12)	21.21(4.55, 38.20)
	Cadmium	0.02 (3e−4, 1.51)	0.0056 (9e−5, 0.37)	27.00 (24.37, 28.17)	0.15 (0.002, 11.33)
	Lead	9.46 (0, 85.39)	0 (0, 0)	0 (0, 0)	78.94 (0, 720.85)
	Methylmercury	3.90 (0.75, 23.94)	0 (0, 0)	0 (0, 0)	34.68 (8.10, 196.17)
Stakeholder Selected Hazards	Acrylamide	5.2e−4 (2.5e−5, 0.0049)	3.7e−4 (1.7e−5, 0.0035)	0.54 (0.055, 0.97)	0.012 (5.8e−4, 0.12)
	Aflatoxin M1	0.001 (2.3e−4, 0.0041)	9.5e−4 (2e−4, 3.6e−4)	0.87 (0.84, 0.9)	0.036 (0.0075, 0.14)
	*Bacillus anthracis*	0.06 (0.012, 0.24)	0.001 (1.9e−4, 0.004)	0.017 (0.013, 0.02)	0.035 (0.0061, 0.15)
	*Clostridium botulinum*	0.04 (0.02, 0.08)	0.0031 (8.9e−4, 0.009)	0.3 (0.1, 0.5)	0.099 (0.018, 0.41)
	*Lathyrus sativus*	1.7 (0.17, 17)	0 (0,0)	0 (0,0)	7.6 (0.16, 110)
	Rift Valley Fever virus	8.8e−6 (3.6e−7, 9.1e−5)	7.2e−8 (2.4e−9, 8.2e−7)	0.01 (0.003, 0.02)	3.4e−7 (4.5e−9, 6e−6)
	Rotavirus	1400 (450, 4100)	0.74 (0.19, 2.4)	5.1e−4 (4.7e−4, 5.5e−4)	63 (17, 200)
	*Staphylococcus aureus*	77 (51, 120)	0.37 (0.16, 0.79)	0.005 (0.0024, 0.009)	0.12 (0.042, 0.3)
	*Taenia saginata*	850 (62, 6300)	0 (0, 0)	0 (0, 0)	36 (0.092, 1100)

a Full details on the derivation of these estimates are provided in Appendix C.

**Risk ranking.** A total of 27 participants from 12 organizations, including seven from government agencies, attended the Ranking workshop. In Round 1, five of the 37 (13.5%) hazards were assigned the same rank by all groups and these ranks were considered final (Appendix C, Fig. 8). In Round 2, an additional 10 of the remaining 32 hazards were assigned the same rank by all groups, resulting in a consensus ranking for 15 out of 37 hazards (41%) and these ranks were considered final (Appendix C, Fig. 11). Of the remaining 17 hazards for which there was no consensus, nine had been ranked High by at least one group and were further considered during a plenary session. Of these nine, seven hazards were collectively ranked as High risk and two as Medium risk. The remaining eight hazards were not considered further. In the final ranking, 12 hazards were ranked High risk, six were ranked Medium risk and 19 were ranked Low risk (Table 4). Ordinal logistic regression suggested that the final rank was mainly driven by mortality, which was consistent with the perceptions that were verbally expressed by some stakeholders.

**Table 4:** Final risk-ranking of 37 foodborne hazards in Ethiopia resulting from Ranking workshop

High risk (12)	Medium risk (6)	Low risk (19)
Aflatoxin B1^a^	*Cryptosporidium* spp.^a^	Acrylamide
Arsenic^a^	*Echinococcus granulosus*^a^	Aflatoxin M1
*Campylobacter* spp.^a^	Hepatitis A virus^a^	*Ascaris* spp.^a^
enteropathogenic *Escherichia coli* (EPEC)^a^	*Listeria monocytogenes*^a^	*Bacillus anthracis*
enterotoxigenic *Escherichia coli* (ETEC)^a^	*Salmonella* Paratyphi^a^	*Brucella* spp.^a^
*Mycobacterium bovis*^a^	Shiga-toxin producing *Escherichia coli* (STEC)^a^	Cadmium^a^
Nontyphoidal *Salmonella enterica*^a^		*Clostridium botulinum*
Norovirus^a^		Dioxins^a^
*Salmonella* Typhi^a^		*Entamoeba histolytica*^a^
*Shigella* spp.^a^		*Fasciola* spp.^a^
Rotavirus		*Giardia* spp.^a^
*Vibrio cholerae*^a^		*Lathyrus sativus*
		Lead^a^
		Methylmercury^a^
		Rift Valley Fever virus
		*Staphylococcus aureus*
		*Taenia saginata*
		*Toxoplasma gondii*^a^
		*Trichinella* spp.^a^

a Denotes a FERG hazard.

**Figure 2:**
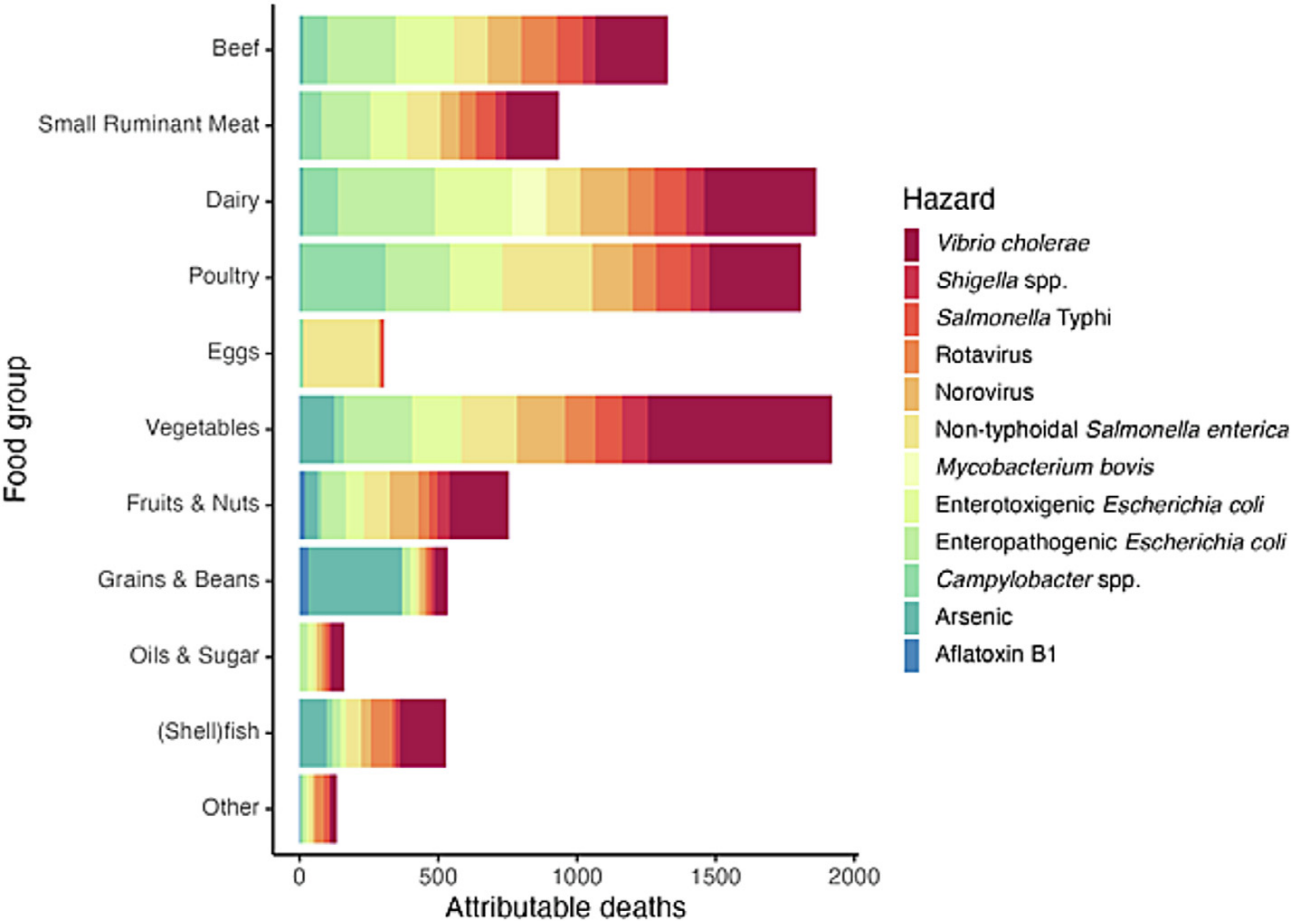
Attribution of foodborne deaths to food group by hazard.

**Figure 3:**
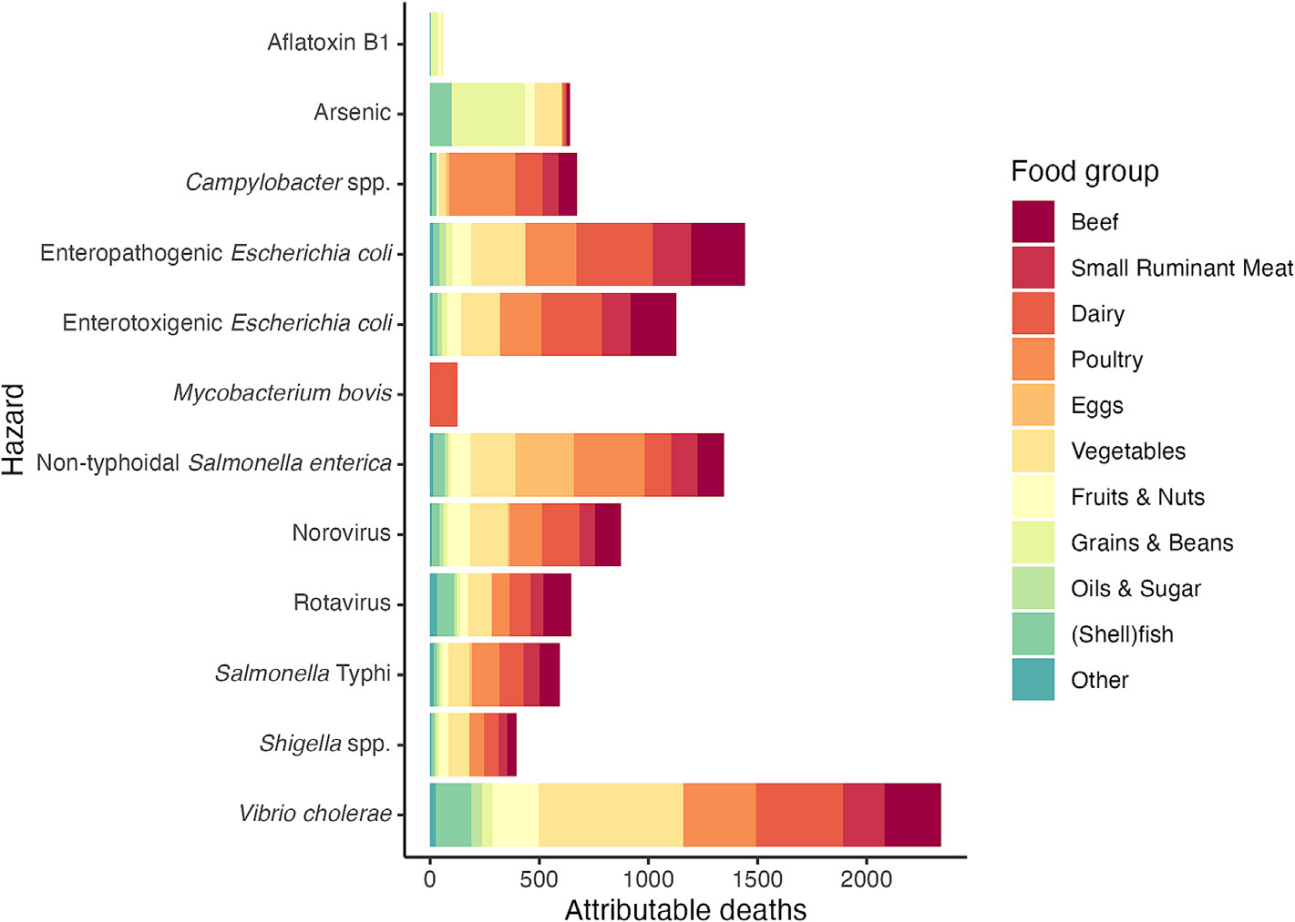
Attribution of foodborne deaths to hazard by food group.

**Estimating attribution.** A total of 15 experts completed Round 1 of the Delphi study and three experts provided revised estimates in Round 2. Vegetables, Dairy, Poultry, and Small Ruminant Meat were estimated to cause the highest number of deaths (Fig. 2), while *Vibrio cholerae*, EPEC, nontyphoidal *Salmonella enterica*, ETEC, and Norovirus were the hazards causing most deaths (Fig. 3).

**Prioritization.** A total of 19 participants from six organizations, including four government agencies, attended the Prioritization workshop to discuss potential strategies for mitigating the risk posed by high-risk foodborne hazards in Ethiopia. Following an overview of previous workshops and derived attribution estimates for deaths (Table 5), participants were broken into groups to focus on the food value chains associated with the largest number of deaths: (1) Vegetables; (2) Dairy; (3) Poultry and Eggs; and (4) Beef and Small Ruminant Meat. To simplify the exercise, Eggs were grouped with Poultry, and Small Ruminant Meat was grouped with Beef.

**Table 5:** Estimated annual number of deaths in Ethiopia due to High risk hazards by food group and type of pathogen category

Food group	Pathogen Category			
	Anthroponotic	Zoonotic	Chemicals	Total
Vegetables	1560	235	125	1920
Dairy	1478	371	13	1862
Poultry and eggs	1195	911	6	2112
Beef and small ruminant meat	1841	401	19	2261

**Figure 4:**
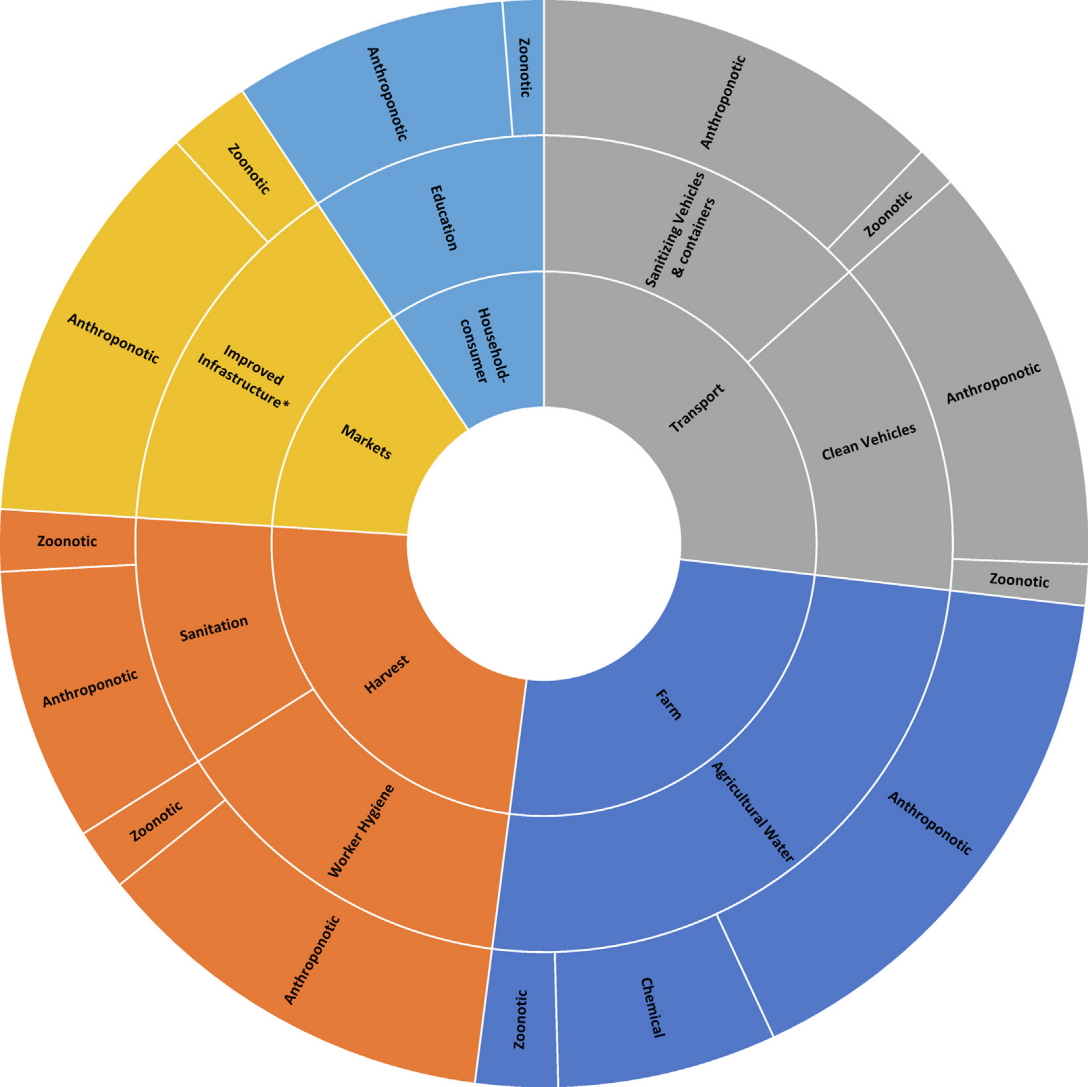
Relative impacts of Supply Chain Control Points in the vegetable value chain on estimated foodborne deaths.

For vegetables, stakeholders identified seven SCCPs across five points in the value chain (Fig. 4). Agricultural water on farm was weighted by stakeholders as contributing the most to preventing FBD deaths, followed by worker hygiene at harvest and cleaning/sanitizing vehicles and containers during transport. Stakeholders noted that knowledge was a problem across all SCCPs and focused training on sanitation and cross-contamination during transportation, growing, handling, storing, and selling of produce could be a short-term goal. Improving water quality and market infrastructure were also considered to be priorities, although stakeholders noted that these should be a long-term strategy since providing an enabling environment for implementing sanitation and hygiene practices (e.g., use of groundwater for irrigation, established sanitation areas in markets, dedicated vehicles for transportation) would require capital investments and a strengthened regulatory framework.

**Figure 5:**
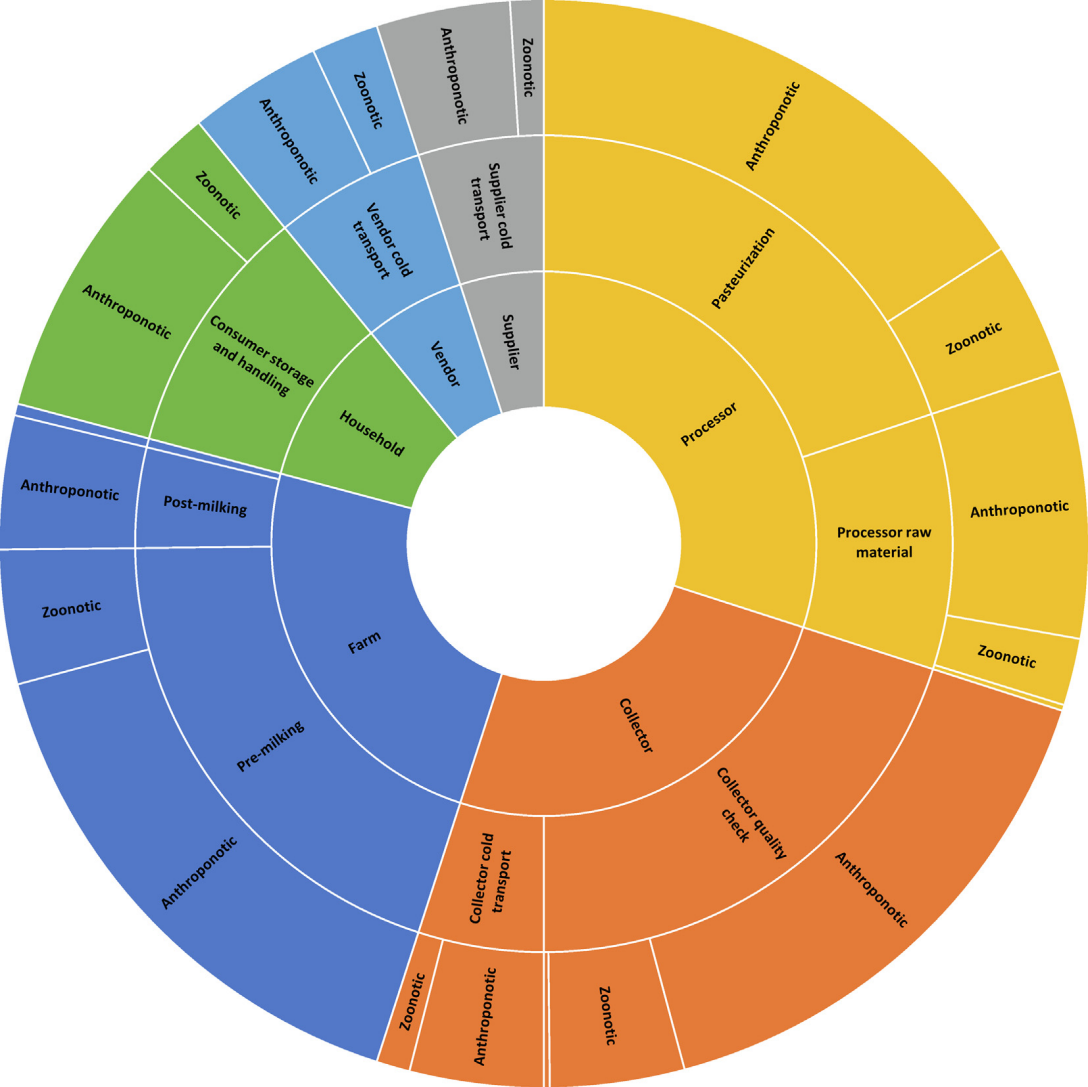
Relative impacts of Supply Chain Control Points in the dairy value chain on estimated foodborne deaths.

For dairy, stakeholders identified ten SCCPs across six points in the value chain (Fig. 5). Premilking sanitation and hygiene, milk collector quality checks, and pasteurization were weighted by stakeholders as contributing the most to preventing FBD deaths. Stakeholders identified the need for increased awareness and training on proper sanitation and food safety as a short-term strategy, but noted that such efforts would likely not be effective without clean water. In the long-term, there needs to be a culture shift to being more food safety-minded, coupled with investments in local equipment (e.g., pasteurization machines) and improved infrastructure for milk collection (e.g., improved roads, reliable electricity, quality-based pricing for producers).

**Figure 6:**
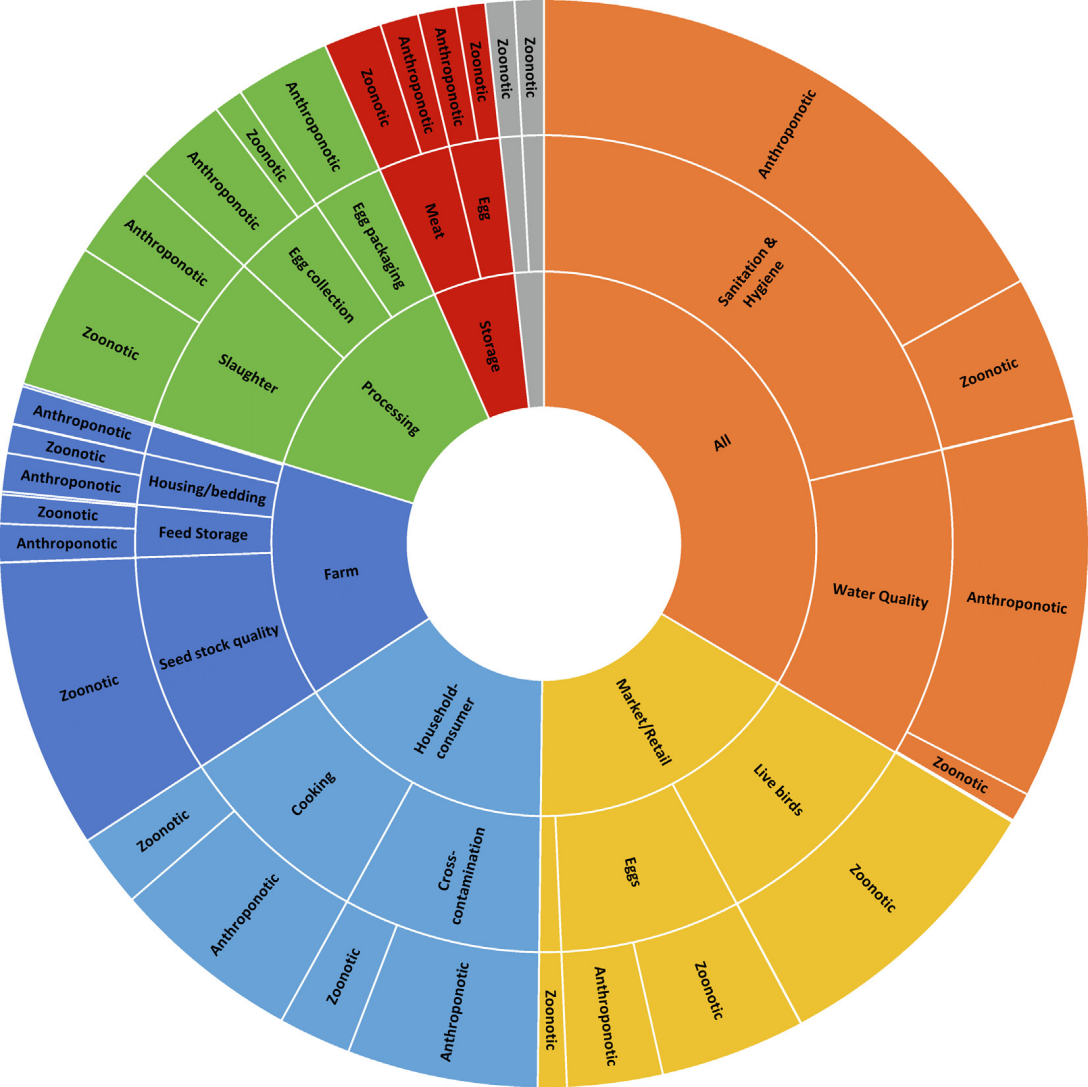
Relative impacts of Supply Chain Control Points in the poultry and egg value chain on estimated foodborne deaths.

For poultry and eggs, stakeholders identified twenty SCCPs in the value chain (Fig. 6). Improving sanitation and hygiene across the entire value chain was identified as having the highest impact in preventing FBD deaths. In the short-term, efforts should be undertaken to improve handwashing practices and the use of personal protective equipment. In the long term, standards and guidelines for sanitation and hygiene need to be established, and control systems and quality checks need to be implemented.

**Figure 7:**
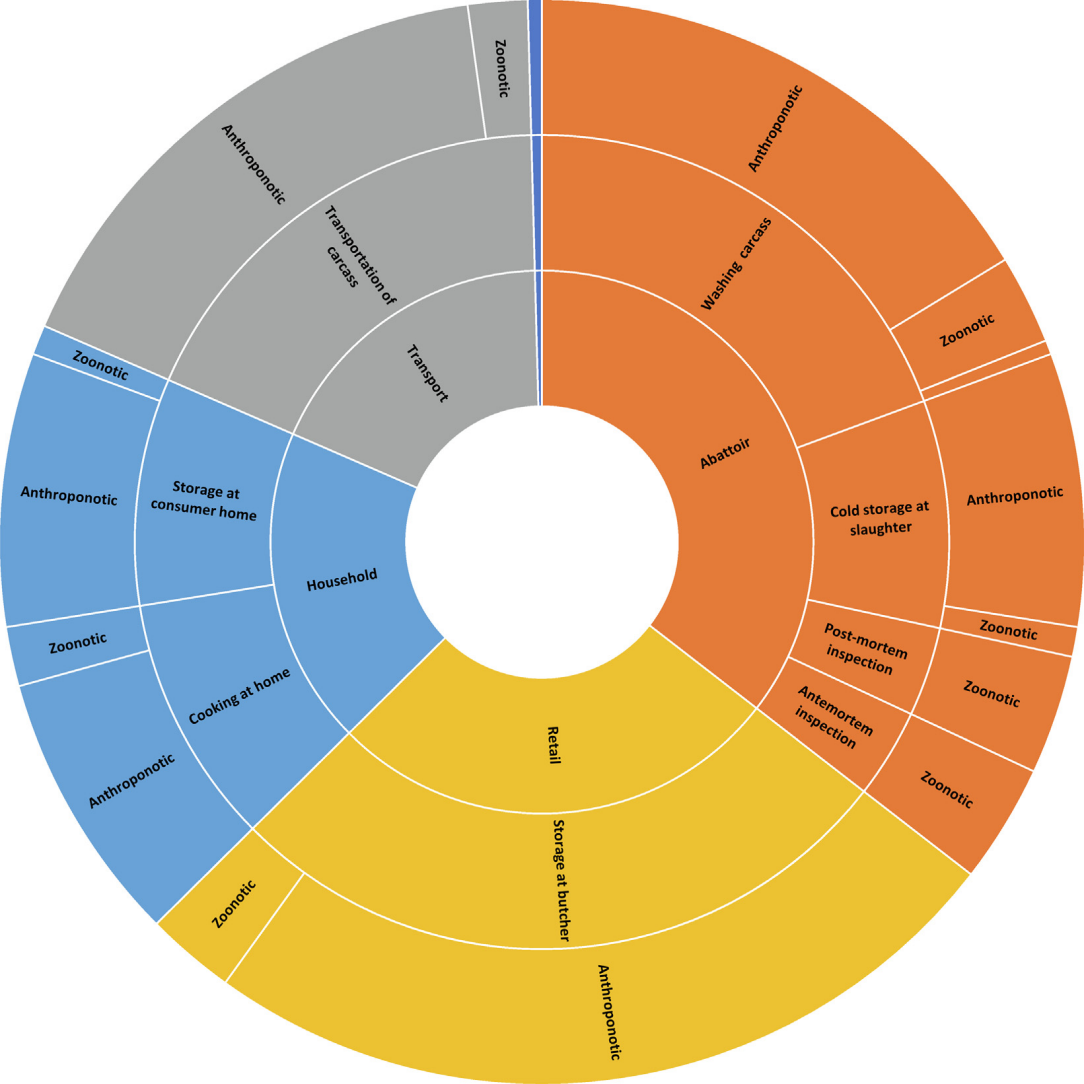
Relative impacts of Supply Chain Control Points in the beef and small ruminant meat value chain on estimated foodborne deaths.

For beef and small ruminants, stakeholders identified ten SCCPs across five points in the value chain (Fig. 7). Storage at retail was identified as having the highest impact on preventing FBD deaths, followed by washing of carcasses at the abattoir and transportation of carcasses from the abattoir to retail. Stakeholders noted the need to update butcher facilities due to inadequate standards for butcher houses. In the short term, livestock stakeholders could be engaged to update and improve the existing standards for butcher houses. Training on regulatory standards would also be of value. In conjunction with this, butchers should be trained on sanitation and hygiene, and an updated sanitation program should be introduced.

In the plenary session, stakeholders expressed concerns about the lack of infrastructure, safe water, and incentives for all value chains. In particular, food and water quality assurance programs that include testing of water samples are needed. Stakeholders noted that water quality is often not checked and existing water standards need to be enforced and updated. Further, standard operating procedures, manuals, and trainings need to be revised, developed, and agreed upon by government agencies with a focus on One Health. Stakeholders also discussed the need for improved coordination and communication using a multisectoral and multidisciplinary approach. During the fishbowl exercise, stakeholders noted there were serious coordination and collaboration issues and voiced concerns that the advances made through the risk ranking and prioritization process would not keep moving forward. Stakeholders challenged each other to identify the root causes and strategies for improvement in these areas as there are many sectors and agencies responsible for progress. It was noted that there is currently a national food safety and nutrition committee that has identified ensuring safe food in all value chains as one of its objectives, but all activities depend on committee members. It was discussed that stakeholders should hold the committee accountable and societal change should be the unifying goal. One suggestion to unify and continue the coordination and communication among the stakeholders was to use the newly initiated World Food Safety Day in Ethiopia to build on the workshop efforts and bring everyone together to advance needed changes.

In the WINFY exercise, stakeholder groups identified at least two requests for each of the other groups. Government agencies were generally asked to ensure that the food safety committee was established with a designated leader, increase funding for food safety, and improve collaboration and coordination. Regulatory agencies were asked to strengthen and enforce food safety regulations, improve communications on guidelines and standards, and share effective policies and procedures among the agencies. Public health agencies were asked to generate science-based evidence on prioritized and emerging food safety risks regularly, strengthen FBD surveillance systems, conduct a national burden of disease study, and advance One Health approaches to food safety. Standards agencies were asked to review, revise and, when necessary, develop new standards for improving food safety in relevant commodities and create awareness about these standards among stakeholders and the public. Research agencies were asked to collaborate on the development of new technologies and interventions, lead the development and implementation of molecular methods, and conserve isolates generated by other agencies. Universities were asked to provide training and capacity building, develop innovative technologies, lead the integration of water safety and health (WASH) and food safety efforts, and improve research collaborations. The nongovernment organization was asked to provide funding, technical support, and capacity building as well as improve research collaborations and communication of research findings. All stakeholders were asked to actively participate in the development of standards. Responses to requests were varied but generally positive; a summary of requests was distributed to participants. Stakeholders did not develop a plan for monitoring the progress of implementing next steps during the workshop but the outcomes informed the development of a National Food Safety Master Plan that was released in May 2024 (Ethiopia Food and Drug Authority, 2024).

## Discussion

The goal of this work was to develop a systematic and transparent framework for ranking and prioritizing food safety risks in low-resource settings, such as LMICs. Risk analysis frameworks have been previously developed (Food and Agriculture Organization of the United Nations (FAO), 2006, CAC, 2007, OIE, 2010) with several published guidance documents supporting their implementation (EFSA-BIOHAZ, 2012, European Food Safety Authority (EFSA) Panel on Biological Hazards (BIOHAZ), 2015, Codex Alimentarius Commission, 2013, FAO, 2017, FAO, 2020). Our efforts built specifically upon the FAO *Guide to Ranking Food Safety Risks at the National Level* (FAO, 2020), using Ethiopia as a case study for risk ranking foodborne hazards based on their disease burden and prioritizing points where food safety interventions might be impactful in reducing that burden.

To our knowledge, this is the first ranking and prioritization of a range of food safety risks in Ethiopia. Previously, the Ethiopian government and the US Centers for Disease Control and Prevention collaborated with stakeholders to rank 34 different zoonotic hazards in Ethiopia (Pieracci et al., 2016). The final ranking was Rabies, Anthrax, Brucellosis, Leptospirosis, and *Echinococcus*, in that order. Of these, Anthrax, Brucellosis, and *Echinococcus* can also be transmitted by food, but were not ranked as High risk based on their disease burden in the current exercise. On the other hand, *Salmonella* and *Mycobacterium bovis* were ranked High in the current exercise but did not make it to the final list of the previous study. Note, however, that the final score of *Salmonella* was tied with Brucellosis and Leptospirosis, and the normalized score of *Mycobacterium bovis* was only 1 percentage point lower. Differences in ranking can potentially be explained by, among other things (e.g., objectives, hazard types), differences in methodology. The ranking of zoonotic hazards used a Multi Criteria Decision Analysis approach, using five criteria with approximately equal weights: severity of human disease (based on case-fatality ratio), proportion of human disease attributable to animal exposure, burden of animal disease, availability of interventions and existing intersectoral collaboration. Using these five criteria means that the final score was an aggregate of elements of risk ranking (the first three criteria) and risk prioritization (the last two criteria). However, risk ranking and risk prioritization should explicitly be separated to help ensure scientific integrity and reduce conflicts of interest (CAC, 2007) and, as such, were separately implemented in our approach (e.g., a hazard would not be prioritized if the disease burden is low, even though interventions are available).

Defining the scope of the risk ranking and prioritization efforts was the first step in our approach. Government risk managers, food safety and epidemiology experts, and other stakeholders played a key role in identifying hazards to be included in the risk ranking and screening of those hazards for relevance and risk potential. Not surprisingly, there were differences of opinion between the experts, who focus narrowly on assessing risks based on their public health impact, and the risk managers, who must consider a broader array of factors (e.g., resources, trade, food security, perceptions) in their decision-making. For example, there was lack of consensus among the risk managers and the experts about the inclusion of one of the initial pathogens. The pathogen was retained in the scope of the risk ranking exercise, at which point there was quick consensus around its ranking, demonstrating how focusing on public health impact first, coupled with careful presentation of evidence, can help align risk managers, risk analysts, and stakeholders and advance evidence-based decision making. Notably, we included a knowledge-building step about risk-based approaches, risk assessment, and risk management. We recommend incorporating this step prior to the scoping work as it helped various stakeholders understand the process for developing the risk ranking and its relationship to the risk-based framework.

Developing the risk ranking process was the second step in our framework, albeit with some modifications to the process outlined in the 2020 FAO risk ranking guide. In particular, we incorporated the identification of the risk metrics into the scoping work as knowing what metrics will be used is critical to gathering the appropriate information and evaluating for data availability. The choice of risk metrics cannot be objectively done since all of the risk metrics are valid; rather it is based on the preferences of the decision-makers as to which metric(s) are most relevant for their context. Multiple risk metrics were presented to stakeholders during the Scoping workshop and five were identified as being relevant in the Ethiopian context. It became apparent during subsequent risk ranking and prioritization efforts that mortality was considered to be the most important metric for decision-making by Ethiopian stakeholders, followed closely by incidence. Even though DALYs provide a more complete picture of disease burden (WHO, 2020), it was the metric that was least well understood by participants and, therefore, used sparingly in the risk ranking. The lack of understanding could be due to participants’ preference to focus on mortality as the key risk metric, but it could also be due to a lack of familiarity with the application of DALYs as a form of integrated metric for evaluating the burden of disease. The FAO guide describes screening for relevance and risk potential, which was initiated at the scoping workshop in the current study. In follow-up steps, we eliminated several hazards due to a lack of data for estimating risk in Ethiopia. Because information gathering and hazard screening can take considerable time, it is important that there is sufficient time between the Scoping and Ranking workshops for data collection, evaluation, and analysis.

The ranking provided by the stakeholders was entirely consistent with a quantitative approach using calculated estimates to rank FBD pathogens, resulting in eight bacteria, two viruses, and two chemicals being considered high-risk hazards. Ethiopian stakeholders ranked bacterial hazards including *Campylobacter* spp., EPEC, ETEC, *Mycobacterium bovis*, nontyphoidal *Salmonella enterica, Salmonella Typhi, Shigella* spp., and *Vibrio cholerae* to be High risk, which is consistent with the WHO global ranking (Havelaar et al., 2015). Likewise, both the WHO global ranking (Havelaar et al., 2015) and the ranking by Ethiopian stakeholders identified Norovirus as an important foodborne virus. While the case-fatality ratio for Norovirus is low, the high incidence associated with the virus results in an appreciable number of deaths (see Appendix C). This highlights the importance of considering many risk metrics during the decision-making process. Ethiopian stakeholders also considered Rotavirus to be High risk, even though Rotavirus was not included in the global ranking. Even though only a small proportion of Rotavirus cases are foodborne, the public health impact of foodborne transmission is appreciable given the low vaccination coverage rate and, subsequently, high number of cases and deaths in Ethiopia (Atalell et al., 2022). Of the four metals, arsenic was the only metal perceived to be High risk. It had the highest estimated incidence rate, mortality rate, and case-fatality ratio (data in Appendix C). Lead, on the other hand, also may cause a high disease burden but was not ranked High risk because the only endpoint associated with this hazard was intellectual disability with no associated mortality. Among the foodborne parasites, *Trichinella* spp. was considered Low risk, consistent with the FAO/WHO global ranking (Havelaar et al., 2015). While the disease burden of *Taenia saginata* was ranked Low risk based on mortality*,* it was estimated to be relatively high in terms of DALYs (data in Appendix C), which is due to the extremely high prevalence of the disease in Ethiopia due to the common consumption of raw beef (Jorga et al, 2020). Of note, stakeholders were keen to include Aflatoxin M1 in the risk ranking during the Scoping workshop because aflatoxin M1 levels in dairy that exceed current Ethiopian standards recently led to destruction and consumer avoidance of milk (Gizachew et al., 2016, Tadesse et al., 2020). Burden estimates were based on a recent risk assessment (SahaTurna et al., 2022) and, despite levels in Ethiopia being among the highest in the world, the public health impact was estimated to be low and Aflatoxin M1 was ranked Low risk.

By design, the risk ranking and prioritization results were informed by the available disease burden and attribution estimates. Stakeholders recognized there are limitations to the available data and would have preferred having a variety of data sources that could be used to calibrate disease burden estimates. However, as in many countries, Ethiopia lacks data on foodborne illness due to developing FBD surveillance systems. However, as demonstrated here, country-level estimates derived by WHO as part of the development of the 2010 global burden of disease estimates for a range of hazards (also known as FERG hazards) provided a starting point. While these are the most comprehensive estimates available, lack of data may have led to under- or over-estimates of the disease burden associated with some hazards (e.g., STEC) and the omission of other hazards (e.g., rotavirus, *Lathyrus sativus*) from the WHO global estimates study (Havelaar et al., 2015). Data for the non-FERG hazards nominated by Ethiopian stakeholders were even more limited, requiring several assumptions to be made in developing burden estimates. For example, data for *Staphylococcus aureus* were only available for high-income countries and, thus, the pathogen was excluded from the global estimates by FERG. Because of the limited data available, our estimates assumed that the incidence in Ethiopia is comparable to the incidence in high-income countries and that the attribution of FBD deaths was proportional to the attribution of FBD illnesses, assumptions that could have led to biased and highly uncertain results. It is important to note that such biases and uncertainty in risk estimates will greatly influence risk ranking and prioritization results. Therefore, we strongly recommend that risk estimates, as well as risk rankings and prioritizations, be updated on a regular basis, particularly when new data become available, and efforts be undertaken to generate high-quality data that is representative of the populations of interest. Ideally, these data would be generated by in-country surveillance systems that are appropriately designed to provide the epidemiologic data needed to estimate burden and attribute illnesses to their sources. We also strongly recommend presenting data in a structured way as it helps ensure transparency and consistency in decision-making. For example, the background document and data dashboard were extremely helpful in the risk ranking process. In particular, the data dashboard enabled us to provide information on multiple risk metrics in a more consumable manner during the Ranking workshop.

In keeping with the FAO 2006 and OIE 2020 risk analysis frameworks, we extended the 2020 FAO guidance by including a prioritization step as proof of concept. This additional step focused on the pathogens identified as High risk, a logical step since prioritization activities should be conditional on ranking results. In this step, an aggregated (i.e., zoonotic, anthroponotic, chemicals) hazards approach was used to identify key points of hazard control in four food value chains, all complex and aggregated in their own right, along with potential risk mitigation strategies. The prioritization effort was achieved in two days by a limited number of individuals, representing key stakeholders, and SCCPs were identified based on the expertise in the room. Ideally, there would be a broader engagement of stakeholders with intimate knowledge of the food value chains and potential risk mitigation strategies as well as a more systematic and thorough evaluation of the risk management options. Even so, the current approach provides for a model of how the risk prioritization process can be informed by quantitative estimates, instead of the frequently used traffic light risk matrices. In particular, it is recommended that risk managers use defined criteria to identify, evaluate, and select risk management options (FAO, 2017). Such decisions are complex and using a structured approach, if designed properly, would help provide clarity around the factors considered and the evidence used in the decision-making process. The SCCPs and risk management options identified here may be considered as a starting point for further work that systematically uses the best available science and well-defined criteria to prioritize the allocation of resources in a consistent, transparent, and well-documented manner. However, this step should be given the time and diligence it deserves and, as such, we recommend that potential SCCPs and associated interventions, including other measures that could help control hazards (e.g., proper operation design, prerequisites, good manufacturing practices), be more fully flushed out in follow-on activities. It is also recommended that, rather than focusing on highly aggregated food chains, such efforts should consider the nuances within each supply chain (e.g., raw milk, yoghurt, soft cheese rather than just dairy) as well as production, consumption, and behavioral patterns (e.g., use of pasteurization, susceptibility of consumers). Finally, cost and feasibility are important considerations in the prioritization and allocation of resources. Ideally, cost-benefit analyses would be conducted to aid in this effort.

Engagement of stakeholders can be challenging but is critical to the success of the proposed approach and advancing food safety priorities. Conducting a situational analysis was helpful in identifying relevant government agencies, academic institutions, and nonprofit organizations to participate in the workshops. In-country partners were indispensable in working with the identified organizations to ensure that representatives with the appropriate background and expertise were engaged at each step of the process. Notably, this approach provided a forum for sustained conversation among stakeholders, which is critical for building momentum for broader strategic planning efforts. For example, it was clear at the first meeting that many of the Ethiopian food safety professionals had not met each other previously. At the end of the project, the stakeholders were familiar with each other and working more collaboratively. Notably, in July 2024, Ethiopia announced a new five-year food safety plan that was developed by more than a dozen institutions (Ethiopian News Agency, 2024), many of which participated in the activities presented here.

## Conclusion

In conclusion, the 2020 FAO risk ranking guide proved to be a valuable tool for guiding our work to help establish a risk-based framework surrounding food safety in Ethiopia. It provided a foundation for ranking and prioritizing food safety risks within four supply chains in Ethiopia. Notably, risk ranking and prioritization efforts should be defined by the needs of the risk managers and informed by the context and values of the society and the agri-food system in which the risk managers function. Therefore, the results are specific to Ethiopia and the risk management questions addressed are not generalizable to other countries. Other countries should conduct their own scoping, risk ranking, and prioritization activities. Further, moving to a risk-based approach for food safety is a continuous improvement process and, as such, this should be seen as a first step in establishing a risk-based food safety system in Ethiopia. Additional work is needed to evaluate, select, implement, monitor, and review risk management options for reducing the public health impact of the high-risk hazards in Ethiopia. The framework proposed here provides a structured, transparent process for making evidence-informed decisions around food safety in low-resource settings that are expected to positively impact public health.

## CRediT authorship contribution statement

**Barbara Kowalcyk:** Writing – original draft, Visualization, Supervision, Resources, Methodology, Investigation, Funding acquisition, Formal analysis, Data curation, Conceptualization. **Leon Gorris:** Writing – review & editing, Methodology, Investigation, Formal analysis. **Janet Buffer:** Writing – review & editing, Project administration, Investigation. **Kathryn Stolte-Carroll:** Writing – review & editing, Project administration, Investigation. **Bashiru C. Bakin:** Writing – review & editing, Investigation. **Allison Howell:** Writing – review & editing, Investigation. **Desalegne Degefaw:** Writing – review & editing, Supervision, Project administration, Methodology, Investigation. **Binyam Moges:** Writing – review & editing, Project administration, Investigation. **Kara Morgan:** Writing – review & editing, Visualization, Supervision, Methodology, Investigation, Formal analysis. **Laura Binkley:** Writing – review & editing, Project administration, Investigation. **Getnet Yimer:** Writing – review & editing, Supervision, Methodology, Investigation. **Arie H. Havelaar:** Writing – original draft, Visualization, Resources, Methodology, Investigation, Funding acquisition, Formal analysis, Data curation, Conceptualization.

## Declaration of competing interest

The authors declare that they have no known competing financial interests or personal relationships that could have appeared to influence the work reported in this paper.
