# Supplementary material for: A Proposed Framework for Ranking and Prioritizing Food Safety Risks in Low Resource Settings Using Foodborne Disease Burden Metrics: A Case Study in Ethiopia
Source: J Food Prot. 2025 Jun 23;88(7):100525. doi: 10.1016/j.jfp.2025.100525 (PMC12548780; doi:10.1016/j.jfp.2025.100525)
Supplement: Supplementary Appendix A [file mmc1.docx]

Table A1. Scoping workshop agenda

| **DAY 1** | | |
| --- | --- | --- |
| **Time** | **Session topics (What)** | **Outcome (Why)** |
| 9.00 am | Registration |  |
| 9.30 am | Welcome and introductions | Everyone is welcome and knows the expected outcomes and agenda of the 4 days of meetings. |
| 10.00 am | Introduction to the project | All participants are aware of the TARTARE project outcomes, timeline, intentions around risk assessment, management, and ranking. Participants understand the need for and possible uses of risk ranking. |
| 10.45 am | *Break* |  |
| 11.05 am | Reviewing list of hazards | Participants are aware of the hazards currently on the list.  Participants have given indications on most important hazards to focus on. |
| 12.00 pm | Understanding a risk-based approach | Participants are refreshed about material covered in online short courses. Highlights are shared by key informants about how risk rankings are conducted and used within the country. |
| 12.45 pm | Closing | Everyone is clear on what has been achieved in that day, what is following, and what is expected to emerge by the end of the week. |
| 1.00 pm | *Lunch* |  |
| **DAY 2** | | |
| **Time** | **Session topics (What)** | **Outcome (Why)** |
| 9.00 pm | Registration |  |
| 9.30 pm | Welcome and introductions | Everyone knows what is planned in the next few days, they have a sense of the agenda and have connected with each other and the topic in a deeper fashion. |
| 10.30 pm | *Break* |  |
| 10.50 pm | Risk definitions | All have shared a clearer understanding about how risk assessment and risk ranking is dealt with in Ethiopia and how this upcoming work could find its place and use. |
| 11.50 pm | Improving the statements of concern and purpose | These statements have been presented then improved with suggestions from the participants. |
| 12.35 pm | Comparing risk-ranking methods | Participants have compared various methods and appreciate value of the one suggested for TARTARE. |
| 12.55 pm | Hazards to be ranked | Everyone has seen the list of hazards from FERG and consolidated the additional hazards identified the previous day to end up with some confidence in the completeness of this list for Ethiopia. |
| 2.00 pm | *Lunch* |  |
| **DAY 3** | | |
| **Time** | **Session Topics (What)** | **Outcome (Why)** |
| 9.00 am | Check-in | All participants have reconnected with each other and the topic. |
| 9.30 am | Consideration of risk metrics | Everyone has understood and appreciated how different risk metrics lead to different risk rankings, and therefore why selecting the metrics being considered is an important step. |
| 9.50 am | Clarifying metrics | All metrics are explored in terms of what they mean and how they are calculated for adults and children. |
| 10.50 am | *Break* |  |
| 11.10 am | Using Risk Assessment results for Risk Ranking | Participants understand how they can use the data dashboard tool to display the data in terms of different metrics and they have provided additional feedback to fine-tune it. |
| 11.50 am | Negotiating helpful metrics | One or more helpful metrics have been chosen as useful and are understood by the participants. |
| 12.50 am | Preparing the next day | Plans for interactions the next day are clear to all and preparations are in place. |
| 1.00 am | *Lunch* |  |
| **DAY 4** | | |
| **Time** | **Session Topic (What)** | **Outcome (Why)** |
| 9.00 am | Registration |  |
| 9.30 am | Welcome and introductions | All participants are reminded about the general outcomes hoped for and the agenda for the morning. |
| 9.45 am | Sharing results from the workshop | All stakeholders are aware of the final list of hazards that will be ranked and of the metrics that are considered most useful and have given their feedback about these. |
| 11.15 am | *Break* |  |
| 11.35 am | Next steps toward risk ranking | Everyone is clear on the reporting from this workshop, next steps, upcoming risk ranking workshop, and (possible) data collection process, etc. |
| 12.00  pm | *Close* |  |

 Table A2. Organizations participating in Scoping workshop

| Federal Agencies |
| --- |
| Animal Products, Veterinary Drug and Feed Quality Assessment Center (under Veterinary Drug and Animal Feed Administration and Control Authority [VDFACA]) |
| Ethiopian Commercial Dairy Products Association |
| Ethiopian Conformity Assessment Enterprise (Under Ethiopian Conformity Assessment Enterprise [ECAE]) |
| Ethiopian Food and Drug Authority |
| Ethiopian Institute of Agricultural Research |
| Ethiopian Livestock Research Institute |
| Ethiopian Meat and Dairy Development Industry Institute |
| Ethiopian Public Health Institute |
| Ethiopian Standardization Agency |
| Ethiopian Veterinary Drug and Feed Administration and Control Agency |
| Ministry of Agriculture - Veterinary Public Health Department |
| Ministry of Agriculture- Crop Directorate |
| Ministry of Water Irrigation and Electricity |
| National Animal Health Diagnostic and Institution Center |
| National Disaster Risk Management Commission |
| Trade Competitions and Consumer Protection Authority |
| Academic Institutions |
| Addis Ababa University |
| Haramaya University |
| Jimma University |
| University of Gondar |
| Private Sector/NGOs/Other |
| Bless Agri food Laboratory |
| Ethiopian Milers Association |
| Feed the Future Value Chain Project |
| Food and Agriculture Organization |
| Global Alliance for Improved Nutrition |
| International Livestock Research Institute |
| Nutrition International |
| Techno Serve |

Table A3. Ranking workshop agenda

| **DAY 1** | | |
| --- | --- | --- |
| **Time** | **Topics (What)** | **Outcome (Why)** |
| 8.30 am | Registration |  |
| 9.00 am | Welcome and introductions,  TARTARE Refresher | Everyone feels welcome and knows the expected outcomes / process of the meeting.  Everyone is clear on the TARTARE project outcomes, timeline, intentions around risk assessment, management, and ranking, and about the 2020 risk scoping workshop and general approach about risk-based planned in general and about risk ranking in particular. |
| 10.15 am | *Break and group photo* |  |
| 10.45 am | Reviewing non-FERG hazards | Data collected on the public health impact of Ethiopia specific non-FERG hazards is clear to all participants so they have a better understanding of all hazards. |
| 11.45 am | Reviewing FERG hazards | Everyone is reminded of important information about the impact of FERG hazards in Ethiopia (includes group work). |
| 12.30 pm | *Lunch* |  |
| 1.30 pm | Meeting the metrics again | The participants are reminded about the metrics chosen at the scoping workshop. The dashboard is introduced. They have discussed the relative importance of each metric in the Ethiopian context. The logic, and tradeoffs, of risk ranking are further clarified (group work). |
| 2.30 pm | Discussing hazards’ initial ranking and group work ranking hazards | The exercise ahead is clear to everyone. Groups are formed and have started ranking hazards, each as per one metric of comparison, adding their rationale to it. |
| 3.00 pm | *Break* |  |
| 3.20 pm | Discussing hazards’ initial ranking and group work ranking hazards | Groups of participants have started ranking hazards, each as per one metric of comparison, adding their rationale to it. |
| 4.10 pm | Taking stock and closing | The groups’ individual (and collective) progress is gauged.  Everyone has drawn lessons and reflections for the day. |
| 4.30 pm | Closed |  |
| **Day 2** | | |
| **Time** | **Session Topic (What)** | **Outcome (Why)** |
| 8.30 am | Registration |  |
| 9.00 am | Welcome and review of the previous day | Everyone has landed again with the topic and is clear on what lies ahead for the day. |
| 9.15 am | Parallel group work ranking hazards (continued) | Groups continue their work on ranking hazards (group work). |
| 10.30 am | Break |  |
| 10.50 am | Reviewing group work results and ranking | Every group has realized what other groups have done, with what rationale (group work). |
| 11.20 am | Plenary review of the group results | The whole group has reviewed what emerges as the collective work and has identified what is still under debate. |
| 12.05 pm | *Lunch* |  |
| 1.05 pm | Group work | Different (reconfigured) groups are working on ranking the hazards including 2 metrics. Their rationale is captured (group work). |
| 2.35 pm | *Break* |  |
| 2.55 pm | Group work review and plenary sense-making | Everyone has reviewed all groups’ work and has collectively singled out what hazards remain to be ranked . |
| 3.55 pm | Closing | Participants have shared their final reflections for the day. |
| 4.10 pm | *Closed* |  |
| **DAY 3** | | |
| **Time** | **Session Topic (What)** | **Outcome (Why)** |
| 8.30 am | Registration |  |
| 9.00 am | Welcome and review of the previous day | Everyone has landed again with the topic and is clear on what lies ahead for the day. |
| 9.15 am | Finalizing risk ranking | In plenary, the group has agreed on the final risk ranking. |
| 10.00 am | *Break* |  |
| 10.15 am | Understanding values underpinning risk prioritization | Everyone has shared which societal values seem important to them, as valuable information for the risk prioritization workshop coming up later. |
| 11.00 am | Any other matter | Any final matter of importance to all is addressed. |
| 11.30 am | Closing | Everyone is clear on the reporting from this workshop, next steps, on the next workshop, on the (possible) data collection process etc. They have collectively assessed the value of this workshop also and are thanked for their participation. |
| 12.00 pm | *Lunch* |  |

Table A4. Organizations participating in Ranking workshop

| Federal Agencies |
| --- |
| Animal Products, Veterinary Drug and Feed Quality Assessment Center (under Veterinary Drug and Animal Feed Administration and Control Authority [VDFACA]) |
| Ethiopian Agricultural Authority |
| Ethiopian Disaster Risk Management Commission |
| Ethiopian Food and Drug Authority |
| Ethiopian Institute of Agricultural Research |
| Ethiopian Livestock Research Institute |
| Ethiopian Meat and Dairy Development Industry Institute |
| Ethiopian Public Health Institute |
| Ethiopian Standardization Agency |
| Ethiopian Veterinary Drug and Feed Administration and Control Agency |
| Ministry of Agriculture - Veterinary Public Health Department |
| Academic Institutions |
| Addis Ababa University |
| Jimma University |
| Haramaya University |
| University of Gondar |
| Private Sector/NGOs/Other |
| Bless Agri food Laboratory |
| Ethiopian Milers Association |
| Millers Association |
| Techno Serve |

Table A5. Prioritization workshop agenda

| **DAY 1** | | | |
| --- | --- | --- | --- |
| **Time** | | **Topics (WHAT?)** | **Outcome (WHY?)** |
| 9.00 am | | Welcome | Everyone is welcome, clear on program and has a sense of who is in the room. |
| 9.30 am | | TARTARE ambitions and progress | Everyone is clear about project again, its ambitions, and what has been achieved + how this workshop fits in the picture. |
| 10.00 am | | Reorientation - starting back from risk ranking workshop | All people present are clear on what happened in the risk ranking workshop. |
| 10.30 am | | *Break* |  |
| 10.50 am | | Our existing knowledge about top-ranked hazards | People have updated information about how we know that these hazards are found in various food groups and about strategies to manage these (i.e., prevent or decrease). |
| 11.50 am | | Supply chain inspection part 1 | In groups, participants have started looking at the supply chain for high risk areas and identified where hazards emerge for each food/pathogen pair, indicating their confidence about this. |
| 12.35 pm | | *Lunch* |  |
| 1.35pm | | Supply chain inspection part 2 | In groups, participants have started looking at the supply chain for high risk areas and identified where hazards emerge for each food/pathogen pair, indicating their confidence about this. |
| 2.20pm | | Reporting back from groups | All group results are reported and known, clear areas of research are highlighted, key critical control points are identified. |
| 3.05pm | | *Break* |  |
| 3.25pm | | Risk management: prelude | Everyone is clear on risk management terminology, they have considered how international standards could be applied domestically. |
| 4.15pm | | Closing |  |
| 4.30pm | | *Closed* |  |
| **DAY 2** | | | |
| **Time** | | **Topics (WHAT?)** | **Outcome (WHY?)** |
| 8.30 am | | Registration |  |
| 9.00 am | | Welcome back | Everyone has landed back with the topic and each other |
| 9.30 am | | Risk management: prelude B2b (70’ - of which 20’ covered today + 15’ optional to finalize group work) | Everyone is clear on risk management terminology; they have considered how international standards could be applied domestically. |
| 10.05 am | | *Break* |  |
| 11.00 am | | Risk management strategies | Food groups have suggested 2-3 prioritized risk management strategies. |
| 12.30 pm | | *Lunch* |  |
| 1.30 pm | | Introducing risk management strategies | Everyone is aware of all the risk management strategies and has identified opportunities and gaps for the food safety roadmap. |
| 2.30 pm | | *Break* |  |
| 2.50 pm | | Additional risk management strategies for a roadmap towards [https://docs.google.com/document/u/0/d/13_UIdxwqBly00IrigPir26vPnecUS01iY_FGEodM4GU/mobilebasic?usp=gmail](https://docs.google.com/document/u/0/d/13_UIdxwqBly00IrigPir26vPnecUS01iY_FGEodM4GU/mobilebasic?usp=gmail#cmnt11)improved food safety | Participants have suggested additional aspects of the roadmap. |
| 4.00 pm | | Closing | All participants have reflected |
| 4.20pm | | *Closed* |  |
| **DAY 3** | | | |
| **Time** | | **Topics (WHAT?)** | **Outcome (WHY?)** |
| 8.30 am | | Check-in |  |
| 9.00 am | | Welcome back  <https://docs.google.com/document/u/0/d/13_UIdxwqBly00IrigPir26vPnecUS01iY_FGEodM4GU/mobilebasic?usp=gmail> | Everyone has landed back with the topic and each other and everyone is reminded about the upcoming nutritional strategy. |
| 09.30 am | | Existing coordination mechanisms: the good, the bad, the ugly and the lovely | Existing coordination mechanisms about addressing food safety are known to all, what works, what doesn’t so well, what would be welcome next. |
| 10.20 am | | *Break* |  |
| 10.40 am | | Improving existing coordination mechanisms | Participants have dreamed the worst possible result about their communication and collaboration to fail food safety, have done a reality check and identified what to stop. |
| 11.20 am | | Further improving collaborations | The key agencies involved have expressed their needs viz. each other and expressed what they can do about it. |
| 12.20pm | Closing | | Everyone has identified what they can do to support the workshop results. We have some feedback about the workshop (content/process) and next steps are clear to all. |
| 12.50pm | *Closed* | |  |

Table A6. Organizations participating in Prioritization workshop

| Federal Agencies |
| --- |
| Animal Products, Veterinary Drug and Feed Quality Assessment Center (under Veterinary Drug and Animal Feed Administration and Control Authority [VDFACA]) |
| Ethiopian Agricultural Authority |
| Ethiopian Food and Drug Authority |
| Ethiopian Institute of Agricultural Research |
| Ethiopian Livestock Research Institute |
| Ethiopian Meat and Dairy Development Industry Institute |
| Ethiopian Public Health Institute |
| Ethiopian Veterinary Drug and Feed Administration and Control Agency |
| Ministry of Agriculture - Veterinary Public Health Department |
| Academic Institutions |
| Addis Ababa University |
| Bahirdar University |
| Haramaya University |
| Oromia State University |
| University of Gondar |
