# Supplementary material for: A Proposed Framework for Ranking and Prioritizing Food Safety Risks in Low Resource Settings Using Foodborne Disease Burden Metrics: A Case Study in Ethiopia
Source: J Food Prot. 2025 Jun 23;88(7):100525. doi: 10.1016/j.jfp.2025.100525 (PMC12548780; doi:10.1016/j.jfp.2025.100525)
Supplement: Supplementary Appendix B [file mmc2.docx]

**TARTARE Burden of foodborne disease in Ethiopia**

**Estimates to support Risk Ranking of Food Safety Hazards**

**Table of Contents**

[Abstract 4](#_Toc185237757)

[List of Acronyms 5](#_Toc185237758)

[Glossary 6](#_Toc185237759)

[Introduction 7](#_Toc185237760)

[Methods and Results 9](#_Toc185237761)

[FERG Hazards 10](#_Toc185237762)

[Metals 11](#_Toc185237763)

[Additional Hazards 11](#_Toc185237764)

[Acrylamide 13](#_Toc185237765)

[Aflatoxin M1 15](#_Toc185237766)

[*Bacillus anthracis* 17](#_Toc185237767)

[*Clostridium botulinum* 19](#_Toc185237768)

[*Lathyrus sativa* 21](#_Toc185237769)

[Rift Valley Fever 23](#_Toc185237770)

[Rotavirus 25](#_Toc185237771)

[*Staphylococcus aureus* 27](#_Toc185237772)

[*Taenia saginata* 29](#_Toc185237773)

[Appendix A: Risk Summaries for FERG Hazards and Metals 31](#_Toc185237774)

[*Brucella* spp. 31](#_Toc185237775)

[*Campylobacter* spp. 31](#_Toc185237776)

[*Cryptosporidium* spp. 31](#_Toc185237777)

[Entamoeba histolytica 32](#_Toc185237778)

[Enteropathogenic *E. coli* (EPEC) 32](#_Toc185237779)

[Enterotoxigenic *E. coli* (ETEC) 32](#_Toc185237780)

[*Giardia* spp. 33](#_Toc185237781)

[Hepatitis A 33](#_Toc185237782)

[*Listeria monocytogenes* 33](#_Toc185237783)

[Non-typhoidal *S.* enterica 34](#_Toc185237784)

[Norovirus 34](#_Toc185237785)

[Shiga toxin-producing *E. coli* 34](#_Toc185237786)

[*Shigella* spp. 35](#_Toc185237787)

[*Vibrio* spp. 35](#_Toc185237788)

[Aflatoxin B1 35](#_Toc185237789)

[*Ascaris* spp 35](#_Toc185237790)

[Dioxins 36](#_Toc185237791)

[*Echinococcus granulosus* 36](#_Toc185237792)

[*Fasciola* spp. 37](#_Toc185237793)

[*Mycobacterium bovis* 37](#_Toc185237794)

[*Salmonella* Paratyphi A 37](#_Toc185237795)

[*Salmonella* Typhi 38](#_Toc185237796)

[*Toxoplasma gondii* 38](#_Toc185237797)

[*Trichinella* spp. 39](#_Toc185237798)

[Appendix B: Risk Summary Sheets for Non-FERG Hazards 40](#_Toc185237799)

[Acrylamide 40](#_Toc185237800)

[Aflatoxin M1 42](#_Toc185237801)

[*Bacillus anthracis* 43](#_Toc185237802)

[*Clostridium botulinum* 44](#_Toc185237803)

[*Lathyrus sativus* 45](#_Toc185237804)

[Rift Valley Fever Virus 47](#_Toc185237805)

[Rotavirus 49](#_Toc185237806)

[*Staphylococcus aureus* 50](#_Toc185237807)

[*Taenia saginata* 52](#_Toc185237808)

[Appendix C: Expert Feedback 53](#_Toc185237809)

[*Bacillus anthracis* 53](#_Toc185237810)

[*Clostridium botulinum* 55](#_Toc185237811)

[*Lathyrus sativus* 56](#_Toc185237812)

[Rift Valley Fever Virus 57](#_Toc185237813)

[Rotavirus 58](#_Toc185237814)

[*Staphylococcus aureus* 59](#_Toc185237815)

[*Taenia saginata* 60](#_Toc185237816)

**Abstract**

This report presents burden estimates for selected foodborne hazards in Ethiopia using 2010 as a reference year. It includes estimates developed by the World Health Organization Foodborne Disease Burden Epidemiology Reference Group for Ethiopia, estimates generated through a different method for four metals, and estimates generated by the TARTARE project for hazards that were not estimated by FERG but were deemed important by Ethiopian food safety stakeholders.

# **List of Acronyms**

CAMPY *Campylobacter*

DALY Disability Adjusted Life Years

DDT Dichlorodiphenyltrichloroethane

DEC Diarrheagenic *Escherichia coli*

DFID United Kingdom Department for International Development

ECAE Ethiopian Conformity Assessment Enterprise

EPHI Ethiopian Public Health Institute

FAO Food and Agriculture Organization of the United Nations

FBD Foodborne Disease

FERG Foodborne Disease Burden Epidemiology Reference Group

GOHi Global One Health initiative

ILRI International Livestock Research Institute

NTS Non-typhoidal *Salmonella*

TARTARE The *Assessment and Management of Risk from Non-typhoidal Salmonella, Diarrheagenic Escherichia coli and Campylobacter in Raw Beef and Dairy in Ethiopia*

VDFACA Veterinary Drug and Animal Feed Administration and Control Authority

YLL Years of Life Lost

# **Glossary**

| Burden of disease | The total, cumulative consequences of a defined disease or a range of harmful diseases with respect to disabilities in a community. Often measured by Disability Adjusted Life Years (DALY)s and can be considered the sum of DALYs across a population. |
| --- | --- |
| Case fatality ratio | Proportion of people who die from a specified disease among all individuals diagnosed with the disease over a certain period of time. |
| DALY | A health gap measure that combines the years of life lost due to premature death (YLL) and the years lived with disability (YLD) from a disease or condition, for varying degrees of severity, making time itself the common metric for death and disability. One DALY equates to 1 year of healthy life lost. |
| Data dashboard | Information management tool that visually displays data. |
| Foodborne Disease | A disease commonly transmitted through ingested food. FBDs comprise a broad group of illnesses, and may be caused by microbial pathogens, parasites, chemical contaminants, and biotoxins. |
| Food Hazard | A biological, chemical, or physical agent in, or condition of, food with the potential to cause an adverse health effect. |
| Incidence rate | Number of new cases of disease during specified time interval. Often expressed in summed person-years of observation or average population during time interval as a denominator. |
| Metric | Measure that is used to track and assess the status of a specific process. |
| Mortality rate | A measure of the frequency of occurrence of death in a defined population during a specified interval. |
| Plenary session | A session presenting a broad range of content. |
| Risk | The likelihood and severity of harm |
| Risk assessment | Evaluation of known or potential effects. Consists of Hazard Identification, Hazard Characterization, Exposure Assessment, and Risk Characterization. |
| Risk-based decision making | A process to ensure that decisions utilize information on likelihood and severity of harm for decision making. |
| Risk-based food safety system | A system that includes consideration of actions that can have the most effective impact on reducing risk. This system includes processes for estimating food safety risk, knowledge of possible interventions for reducing risk, and a set of priorities that are informed by these estimates and knowledge. |
| Risk-based framework | A guide for helping everyone in the system use risk information to inform their actions. |
| Risk management | The process of weighing policy alternatives in the light of the results of risk assessment and, if required, selecting, and implementing appropriate control options, including regulatory measures. |
| Risk-ranking process | An analysis and ordering of hazards based on risk |
| Stakeholder | Person or group with an invested interest or concern about the matter at hand. |
| Statement of concern | A description in simple terms of the motivation for conducting the risk ranking exercise. |
| Statement of purpose and objectives | Concise paragraph describing the management goals of the risk ranking effort. |
| Years of Life Lost (YLL) | The number of deaths due to a specific disease or condition multiplied by the standard life expectancy at the age at which death occurs. |
| Zoonotic disease | A disease spread between animals and people and can be caused by viruses, bacteria, parasites, and fungi. |

# **Introduction**

According to the World Health Organization (WHO), 420,000 deaths occurred globally among 600 million cases of foodborne disease (FBD) in 2010 and 230,000 of these deaths were due to foodborne hazards which primarily cause diarrheal disease. Seven diarrheal disease hazards (Norovirus, *Campylobacter* spp., enteropathogenic *Escherichia coli* (EPEC), Enterotoxigenic *E. coli* (ETEC), non-typhoidal *Salmonella enterica* (NTS), *Shigella* spp. and *Vibrio cholerae*) were identified to each cause a global foodborne disease burden of 1–3 million disability-adjusted life years (DALYs), as estimated for 2010 (Havelaar et al., 2015).

It was estimated that Africa had the highest FBD burden of all regions analyzed (1,230 DALYs per 100,000 population compared to a global average of 477 DALYs per 100,000 population). By assuming 1 lost DALY equates to a productivity loss of 1 unit of Gross national Income per capita, the World Bank has estimated that the economic cost of FBD in low- and middle-income countries (LMIC) amounts to $95.2 billion per year (Jaffe *et al.*, 2019), nearly all of which is attributable to food bought in domestic markets, and the annual cost of treating foodborne illnesses is estimated at $15 billion. While both assessments only capture part of the total burden and cost of FBD in LMIC, important public health and economic benefits could be achieved by improving food safety in in such countries.

The aim the TARTARE project (The Assessment and Management of Risk from non-typhoidal *Salmonella*, diarrheagenic *Escherichia coli* and *Campylobacter* in Raw Beef and Dairy in Ethiopia) is to reduce morbidity and mortality from FBD by developing a risk-based framework for making decisions and allocating resources around food safety across the food systems in LMICs using Ethiopia as a model country. TARTARE will use mixed methods to address three overarching research questions:

1. What are the public health burden and costs associated with the three selected pathogens in Ethiopia?
2. What are cost-effective, gender sensitive, and socio-culturally acceptable approaches to mitigating public health risks associated with CAMPY, NTS and STEC in raw beef and dairy products?
3. Where should resources be allocated nationally to effectively reduce FBD risk from all causes in Ethiopia?

This report is relevant for Objective 3 which aims to provide stakeholders with a framework for systematically informing objective, evidence-based decisions around food safety. This will be accomplished partly by organizing a series of workshops with governmental decision makers and relevant stakeholders using methods recommended by the United Nations Food and Agriculture Organization (FAO, 2020) for ranking foodborne hazards based on risk estimates for the burden of foodborne disease.

The first step was a Scoping Workshop held in March 2020 in Addis Ababa, which was attended by 37 stakeholders. As noted in the TARTARE scoping workshop report, a *Statement of Concern* and *Statement of Purpose* for the Risk Ranking were developed (Table 1).

***Table 1. Statement of Concern and Statement of Purpose for TARTARE Risk Ranking***

Statement of Concern

**According to the World Health Organization, foodborne disease is an important public health problem in Africa. Many stakeholders in Ethiopia are poised to start addressing various foodborne risks posing varying levels of risk to consumers. However, many food safety stakeholders in Ethiopia are working independently without a shared set of priorities, which will have less impact in preventing and reducing the public health impact (e.g., mortality, morbidity, disability).**

Statement of Purpose

**The purpose of this risk ranking is to identify the hazards that are relatively higher priorities for Ethiopia in terms of their contribution to the overall public health burden (e.g., mortality, disability, morbidity) of foodborne disease. Ranked risks and a final prioritized set of food hazards can inform stakeholders working on food safety in Ethiopia. These tools can leverage their collaborative effort and available resources in a systems approach for a greater impact in risk reduction.**

The stakeholders also nominated, screened and selected hazards to include in the risk ranking. In 2015, the World Health Organization (WHO) Foodborne Disease Burden Epidemiology Reference Group (FERG) published [global and regional foodborne disease illness estimates](https://journals.plos.org/plosmedicine/article?id=10.1371/journal.pmed.1001923) for 31 foodborne hazards (Havelaar *et al.*, 2015) and, in 2019, global estimates for four metals (arsenic, cadmium, lead and methylmercury) were published (Gibb *et al.*, 2019). Seven of the FERG hazards were assumed to not occur in Ethiopia and/or Africa (*E. multilocularis*, *C. sinensis*, intestinal flukes, *Opistorchis* spp., *T. solium*, *Paragonimus* spp., cassava cyanide). Country level estimates for the remaining 28 hazards were obtained for Ethiopia and selected for inclusion in the risk ranking. Stakeholders identified 15 additional hazards/categories of hazards for inclusion. There was insufficient evidence to support the generation of burden estimates for six of the additional hazards (Aflatoxin G1, other aflatoxins, ochratoxin, formalin, pesticides and antibiotics). Aflatoxin and other aflatoxins (except aflatoxin M1) typically co-occur with aflatoxin B1 (AFB1), but in lower concentrations. Therefore, the risk is expected to be lower than the risk of AFB1 while preventive measures against AFB1 are also expected to reduce the risk of other aflatoxins. Therefore, 35 hazards were selected for the risk ranking (Table 2).

***Table 2. List of hazards to be considered in TARTARE Risk Ranking***

| **FERG Hazards** | |
| --- | --- |
| 1. Norovirus 2. *Campylobacter* spp. 3. Enteropathogenic *E. coli* 4. Enterotoxigenic *E. coli* 5. Shiga toxin-producing *E. coli* 6. Non-typhoidal *S. enterica* 7. *Shigella* spp. 8. *Vibrio cholerae* 9. *Cryptosporidium* spp. 10. *Entamoeba histolytica* 11. *Giardia* spp. 12. Hepatitis A virus | 1. *Brucella* spp. 2. *Listeria monocytogenes* 3. *Mycobacterium bovis* 4. *Salmonella* Paratyphi A 5. *Salmonella* Typhi 6. *Toxoplasma gondii* 7. *Echinococcus granulosus* 8. *Ascaris* spp. 9. *Trichinella* spp. 10. *Fasciola* spp. 11. Aflatoxin B1 12. Dioxins |
| **Metals** | |
| 1. Arsenic 2. Cadmium | 1. Lead 2. Methylmercury |
| **Non-FERG Hazards** | |
| 1. Acrylamide 2. Aflatoxin M1 3. *Bacillus anthracis* 4. *Clostridium botulinum* 5. *Lathyrus sativa* | 1. Rift Valley Fever 2. Rotavirus 3. *Staphylococcus aureus* 4. *Taenia saginata* |

Stakeholders also reviewed the risk metrics used by FERG for the global burden of disease estimates and identified five that were most meaningful to Ethiopian decision-makers:

1. Incidence rate
2. Mortality rate
3. Years of life lost (YLL) rate
4. Disability adjusted life years (DALY) rate
5. Case fatality ratio

Definitions for each risk metric are provided in the Glossary and additional details on DALYs are provided in Table 3. Four of these metrics were retained for the risk ranking.

***Table 3. Defining Disability Adjusted Life Years***

**Disability Adjusted Life Years**

One DALY can be thought of as one lost year of "healthy" life. The sum of these DALYs across the population, or the burden of disease, can be thought of as a measurement of the gap between current health status and an ideal health situation where the entire population lives to an advanced age, free of disease and disability.

YLL correspond to the number of deaths in a particular time period multiplied by the standard life expectancy at the age at which death occurs.

YLD correspond to the number of incident cases in a particular time period multiplied by the average duration of the disease and a disability weight factor that reflects the severity of the disease on a scale from 0 (perfect health) to 1 (dead).

In a hazard-based approach, the burden includes all health states, i.e., symptoms and sequelae, including death, that are causally related to of a specific hazard, and which may become manifest at different time scales and/or have different severity levels.

Source: <https://www.who.int/healthinfo/global_burden_disease/metrics_daly/en/>, (Devleesschauwer *et al.* 2015).

This report provides risk estimates to inform the risk ranking and risk prioritization workshops. It includes estimates of the burden of foodborne disease in Ethiopia from the FERG estimates, estimates generated through a different method for four metals, and estimates generated by the project for hazards that were not estimated by FERG. Concerning the risk ranking workshop, YLL estimates are not included in this report since this metric is highly correlated with other risk metrics, particularly mortality rate. Results from the various workshops will be used to inform Ethiopian food safety decision makers about the relative risk of different food hazards in Ethiopia.

**References**

Devleesschauwer B, Haagsma JA, Angulo FJ, Bellinger DC, Cole D, Dopfer D, et al. Methodological Framework for World Health Organization Estimates of the Global Burden of Foodborne Disease. PLoS One. 2015;10(12):e0142498.

Havelaar AH, Kirk MD, Torgerson PR, Gibb HJ, Hald T, Lake RJ, et al. World Health Organization Global Estimates and Regional Comparisons of the Burden of Foodborne Disease in 2010. PLoS Med. 2015;12(12):e1001923.

Jaffee S, Henson S, Unnevehr L, Grace D, Cassou E. The Safe Food Imperative: Accelerating Progress in Low- and Middle-Income Countries. Washington, D.C.: World Bank; 2019.

# **Methods and Results**

Estimates for the four selected risk metrics were calculated for each of the 37 hazards included in the TARTARE Risk Ranking. Since the FERG estimates are the most recent available global estimates, 2010 was used as the reference year for all burden estimates and the population size of Ethiopia was assumed to be 87.64 million (GBD, 2019). All data extraction, manipulation, plots and statistical testing were generated in **R** statistical software version 3.6.0 and later. The data dashboard was generated using the R package *shiny*.

## **FERG Hazards**

WHO FERG estimates provide the best currently available basis for risk ranking of foodborne hazards. The FERG global and regional burden estimates have been published in a range of research papers, mainly in PLOS Medicine and PLOS One (https://collections.plos.org/collection/ferg2015/). Country-level estimates were available but unpublished as these have not been cleared by the Member States. We, therefore, sought and obtained permission of the Ethiopian government to publish country-specific burden of disease estimates for the FERG hazards (Table 4).

***Table 4. Best estimates (95% uncertainty intervals) for risk metrics for Ethiopia by FERG hazard***

| **Pathogen** | **Incidence rate**  **(per 100,000)** | **Mortality rate**  **(per 100,000)** | **Case-fatality ratio (%)** | **DALYs rate**  **(per 100,000)** |
| --- | --- | --- | --- | --- |
| *Brucella* spp. | 1  (0.013, 63) | 0.65  (0.000065, 2) | 0.50  (0.34, 0.66) | 0.36  (0.004, 19) |
| *Campylobacter* spp*.* | 2152  (309, 8391) | 0.75  (0.35, 1.26) | 0.034  (0.0089, 0.23) | 69  (32, 115) |
| *Cryptosporidium* spp. | 186  (0, 909) | 0.15  (0, 0.53) | 0.075  (0.024, 0.28) | 12  (0, 44) |
| *Entamoeba histolytica* | 742  (0, 4288) | 0.05  (0, 0.44) | 0.0068  (0.001, 0.084) | 5  (0, 40) |
| Enteropathogenic *E. coli* (EPEC) | 430  (17, 1331) | 1.67  (0.073, 3.98) | 0.38  (0.16, 0.99) | 136  (6, 321) |
| Enterotoxigenic *E. coli* (ETEC) | 939  (141, 2694) | 1.27  (0.21, 2.86) | 0.13  (0.054, 0.38) | 103  (17, 236) |
| *Giardia* spp*.* | 663  (0, 3060) | 0  (0, 0) | 0  (0, 0) | 0.68  (0, 3) |
| Hepatitis A | 337  (45, 1117) | 0.671  (0.097, 2.18) | 0.20  (0.051, 0.79) | 33  (5, 103) |
| *Listeria monocytogenes* | 0.14  (0.000025, 2.5) | 0.03  (0.0000056, 0.055) | 22.39  (19.24, 25.77) | 1  (0.0002, 21) |
| Non-typhoidal *S. enterica* | 875  (100, 3159) | 1.55  (0.34, 2.65) | 0.17  (0.053, 0.77) | 116  (25, 201) |
| Norovirus | 1609  (0, 5882) | 1  (0, 3) | 0.063  (0.026, 0.14) | 76  (0, 226) |
| Shiga toxin-producing *E. coli* (STEC) | 0.5  (0.07, 2) | 0.000057  (0.0000052, 0.00024) | 0.011  (0.0045, 0.024) | 0.0048  (0.0006, 0.02) |
| Shigella spp. | 435  (0, 3296) | 0.45  (0, 1.79) | 0.093  (0.024, 0.72) | 37  (0, 147) |
| Vibrio spp. | 72  (2, 207) | 2.72  (0.075, 7.30) | 3.80  (2.61, 5.01) | 190  (5, 511) |
| Aflatoxin B1 | 0.019  (0.00019, 0.094) | 0.013  (0.00017, 0.085) | 90.19  (90.19, 90.19) | 0.46  (0.0062, 3) |
| *Ascaris* spp*.* | 92  (17, 164) | 0.0093  (0.000013, 0.01) | 0.011  (0.000037, 0.10) | 6  (1, 14) |
| Dioxins | 0.092  (0.003, 9.30) | 0  (0, 0) | 0  (0, 0) | 0.11  (0.0033, 11) |
| *Echinococcus granulosus* | 2  (0.5, 3) | 0.017  (0.0036, 0.054) | 1.09  (0.40, 2.51) | 1.43  (0.036, 4) |
| *Fasciola* spp*.* | 0.0052  (0.0018, 0.015) | 0  (0, 0) | 0  (0, 0) | 0.038  (0.013, 0.1) |
| *Mycobacterium bovis* | 7  (4, 10) | 0.37  (0.22, 0.54) | 5.48  (3.63, 8.21) | 22  (13, 32) |
| *Salmonella* Paratyphi A | 24  (0, 87) | 0.157  (0, 0.56) | 0.65  (0.65, 0.65) | 11  (0, 40) |
| *Salmonella* Typhi | 106  (0, 378) | 0.68  (0, 2.44) | 0.65  (0.65, 0.65) | 49  (0, 174) |
| *Toxoplasma gondii* | 353  (163, 617) | 0.023  (0.0082, 0.05) | 0.0065  (0.0033, 0.012) | 28  (13, 51) |
| *Trichinella spp.* | 0.00066  (0.00022, 0.001) | 0.000024  (0.0000078, 0.00004) | 3.57  (3.57, 3.57) | 0.0014  (0.00047, 0.0024) |

**References**

Havelaar AH, Kirk MD, Torgerson PR, Gibb HJ, Hald T, Lake RJ, et al. World Health Organization Global Estimates and Regional Comparisons of the Burden of Foodborne Disease in 2010. PLoS Med. 2015;12(12):e1001923.

## **Metals**

Estimates for arsenic, cadmium, lead and methylmercury were extracted from Gibb *et al.*, 2019. Since these are rates, no adjustments needed to be made for population size.

***Table 5. Best estimates (95% uncertainty intervals) for risk metrics for Ethiopia by metal group***

| **Metal** | **Incidence rate**  **(per 100,000)** | **Mortality rate**  **(per 100,000)** | **Case-fatality ratio (%)** | **DALYs rate**  **(per 100,000)** |
| --- | --- | --- | --- | --- |
| Arsenic | 2.39  (0.51, 4.31) | 0.65  (0.14, 1.67) | 27.12  (27.12, 27.12) | 21.21  (4.55, 38.20) |
| Cadmium | 0.02  (0.0003, 1.51) | 0.0056  (0.00009, 0.37) | 27.00  (24.37, 28.17) | 0.15  (0.002, 11.33) |
| Lead | 9.46  (0, 85.39) | 0  (0, 0) | 0  (0, 0) | 78.94  (0, 720.85) |
| Methylmercury | 3.90  (0.75, 23.94) | 0  (0, 0) | 0  (0, 0) | 34.68  (8.10, 196.17) |

**References**

Gibb H, Devleesschauwer B, Bolger PM, Wu F, Ezendam J, Cliff J, et al. World Health Organization estimates of the global and regional disease burden of four foodborne chemical toxins, 2010: a data synthesis [version 1; referees: 1 approved with reservations]. F1000Research. 2015;4:1393.

## **Additional Hazards**

Mean estimates and 95% uncertainty intervals (UI) were calculated for each of the selected risk metrics for the 9 additional hazards selected by stakeholders. Data were extracted from the literature, when available. In the absence of data, assumptions were made, as noted below. These assumptions were reviewed with Ethiopian experts and, when appropriate, adjusted (Appendix C).

We used a standardized method to estimate uncertainty for each input. Whenever possible, uncertainty estimates were computed from reported data. If no data were available to assess uncertainty, we assumed broad uncertainty. For age of onset, we assumed uncertainty bounds were ± 20 years of the midpoint. In all other cases, we divided/multiplied the midpoint by 10 to obtain lower and upper uncertainty bounds. If data were available, uncertainty in proportions (e.g., case-fatality ratio) was modeled as a Beta distribution while uncertainty in rates was modeled with a Gamma distribution (Vose, 2008). Both distributions can be modeled by simulation in R or in a spreadsheet as analytical solutions are available.

Once inputs and assumptions were finalized, the best estimates and UI were calculated, as outlined below, for each risk metric for each pathogen. In many cases, this involved multiplying or dividing two uncertainty distributions. If samples from these distributions were available, this could be achieved using Monte Carlo simulations. However, in many cases, we only had an estimate of the mean and a 95% uncertainty interval. We, therefore, used an approximate solution and assumed that uncertainty can appropriately be modeled by lognormal distributions. The sum of two distributions on the log scale is the same as the product of two distributions on the arithmetic scale. Similarly, the difference between two distributions on the log scale is the same as the ratio of two distributions on the arithmetic scale. The approximate analytical approach has the added advantage that it can also be applied in a spreadsheet if there are no resources to model in a statistical language such as R due to limited resources.

Due to lack of data, it was not possible to separately estimate the burden for under and over 5-years of age.

### **Acrylamide**

Acrylamide (AA) is a chemical formed as a by-product of the Maillard reaction in certain foods, especially in those foods containing asparagine and reducing sugars that are prepared at temperatures above 120 °C and at low moisture levels. Fried potato products, coffee, biscuits, crackers, crisp bread and soft bread may contribute most to AA exposure of consumers. Carcinogenicity, genotoxicity, neurotoxicity, and reproductive toxicity were reported as adverse health effects of human exposure to AA (Dibaba *et al.*, 2018).

**Rationale for inclusion**

Since the recognition of AA as a carcinogen occurring in food in 2002, several researchers have studied its formation and occurrence in heat processed food. There are very limited studies on the level of AA in foods consumed in different parts of Africa. It is not known which foods contribute to significant dietary AA exposure in Ethiopia. Potential dietary AA exposure is of concern given that most staple diets consumed in Africa are made of carbohydrate-based foods subjected to varying degrees of heat during processing.

**Available data**

No estimates of the incidence, deaths, mortality or DALYs of foodborne AA are available for Ethiopia.

There are data from IHME GBD 2010 on the total number of cancer cases and cancer deaths by affected site in Ethiopia as well as DALYs/year associated with these cancers (GBD, 2019). Since certain types of cancer (breast cancer, kidney cancer, ovarian cancer and uterine cancer (endometrial cancer)) are predominantly associated with AA exposure (Jakobsen *et al.*, 2016), we used data on these for both sexes in Ethiopia as a starting point. GBD 2010 estimates for Addis Ababa, urban Ethiopia (assumed to be proportional to Addis Ababa data) and Ethiopia were used as lower, middle and upper estimates for, respectively, the overall distribution for cancer incidence, cancer deaths and cancer DALYs. We chose these populations because foods associated with AA exposure are typically consumed by higher income households, who are more likely to reside in urban areas. Addis Ababa was, therefore, assumed to be the lower exposure level for the country, urban areas of the country were assumed to represent the midpoint and the total population of Ethiopia overall was assumed as the upper level.

To estimate the proportion of the four most relevant cancer types for which cases attributable to AA exposure, we used data from a Danish study that estimated that dietary AA exposure causes 0.021% of these specific cancer types in Denmark (Jakobsen *et al.*, 2016). Uncertainty estimates were not provided so we estimated the upper and lower bounds by multiplying/dividing by 10. We also assumed that AA associated exposure would be comparable between Denmark and Ethiopia.

Assuming a similar proportion of cancer cases in Ethiopia can be attributed to dietary AA exposure, we calculated:

$${Incidence}_{AA}= ET cancer incidence*proportion of cases attributed to AA$$

$${Incidence rate}_{AA}= \frac{{Mortality}_{AA}}{ET population size}*100,000$$

$${Mortality}_{AA}= ET cancer mortality*proportion of cases attributed to AA$$

$${Mortality rate}_{AA}= \frac{{Mortality}_{AA}}{ET population size}*100,000$$

$${Case fatality ratio}_{AA}= \frac{{Mortality}_{AA}}{{Incidence}_{AA}}$$

$${DALYs}_{AA}= ET cancer DALYs*proportion of cases attributed to AA$$

$${DALYs rate}_{AA}= \frac{{DALYs}_{AA}}{ET population size}*100,000$$

$${DALYs per case}_{AA}= \frac{{DALYs}_{AA}}{{Incidence}_{AA}}$$

Incidence, mortality, DALYs and DALYs per case were estimated using the approximate approach described above, assuming lognormal distributions. CFR was calculated using a beta distribution. Input data are summarized in Table 6. Best estimates and uncertainty estimates for risk metrics are provided in Table 7, using 2010 data.

| **Table 6. Acrylamide input data** | | | |
| --- | --- | --- | --- |
| **Metric** | **Lower** | **Middle** | **Upper** |
| Cancer Incidence (GBD, 2010) | 410 | 2000 | 5300 |
| Cancer Mortality (GBD, 2010) | 270 | 1300 | 3900 |
| Cancer DALYs (GBD, 2010) | 9300 | 45000 | 130000 |
| Proportion of cases attributed to Acrylamide (Jakobsen et al., 2016 with adjustments) | 0.000021 | 0.00021 | 0.0021 |

| **Table 7. Acrylamide foodborne estimates by risk metric** | | | |
| --- | --- | --- | --- |
| **Metric** | **Lower** | **Middle** | **Upper** |
| Incidence (Cases per year) | 0.022 | 0.46 | 4.3 |
| Incidence per 100000 | 0.000025 | 0.00052 | 0.0049 |
| Mortality (deaths per year) | 0.015 | 0.32 | 3.1 |
| Mortality per 100000 | 0.000017 | 0.00037 | 0.0035 |
| Case Fatality Ratio | 0.055 | 0.54 | 0.97 |
| DALYs | 0.51 | 11 | 99 |
| DALYs per 100000 | 0.00058 | 0.012 | 0.11 |
| DALYs per case | 23 | 23 | 23 |

**References**

Dibaba, Kumela, Lelise Tilahun, Neela Satheesh, and Melkayo Geremu. “Acrylamide Occurrence in Keribo: Ethiopian Traditional Fermented Beverage.” Food Control 86 (April 1, 2018): 77–82. <https://doi.org/10.1016/j.foodcont.2017.11.016>

[Global Burden of Disease Study 2019 (GBD 2019)](https://ghdx.healthdata.org/record/ihme-data/gbd-2016-cancer-incidence-mortality-years-life-lost-years)  https://vizhub.healthdata.org/gbd-results/

Jakobsen, L. S., Granby, K., Knudsen, V. K., Nauta, M., Pires, S. M., & Poulsen, M. (2016). Burden of disease of dietary exposure to acrylamide in Denmark. Food and Chemical Toxicology, 90, 151- 159.

### **Aflatoxin M1**

Aflatoxin M1 (AFM1), a hydroxylated metabolite of Aflatoxin B1 (AFB1), is secreted in milk from mammalian species or excreted in urine. Hence, AFM1 may be present in milk and other dairy products sourced from cows that consumed AFB1 in their feed. AFB1 is an established carcinogen, causing hepatocellular carcinoma (HCC), i.e., liver cancer. There is also evidence from animal studies, but not from human studies, that AFM1 causes HCC, but the cancer potency of AFM1 is ten times lower than of AFB1 (JECFA references).

**Rationale for inclusion**

Based on both average daily liquid milk consumption patterns and levels of AFM1 contamination in milk, a recent study revealed that globally, Ethiopia, Mexico, Sudan, Syria, and Pakistan have highest exposure levels to AFM1 (Saha Turna and Wu, 2021). This research found that the Average Daily Dose of AFM1 in Ethiopia from consuming liquid milk is 0.79 ng/kg bw/day. For milk, Ethiopia has adopted the maximum permissible limit of 0.05 µg/kg set by the European Commission (EU, 2006). This level is frequently exceeded (Hoddinott *et al.*, 2015), causing concern among regulators and the public and destruction of large volumes of milk.

**Available data**

No estimates of the incidence, deaths, mortality or DALYs of foodborne AFM1 are available for Ethiopia.

The incidence rate of HCC by exposure to AFM1 in Ethiopia was estimated using data from a recently published quantitative cancer risk assessment that analyzed extensive datasets of national population sizes, dairy consumption patterns, AFM1 concentrations in milk in 40 nations, and chronic HBV prevalence (Saha Turna and Wu, 2021; Saha Turna *et al.*, 2022). For the mid-value, we assumed interaction between AFM1 and hepatitis-B virus (HBV); the low value assumed no such interaction. As a high value, we assumed that AFM1 was as toxic as its parent compound AFB1. We then calculated:

${Incidence}_{AFM1}={Incidence rate}_{AFM1}*ET population size$

FERG estimated the total number of foodborne cases, deaths and DALYs for AFB1 (Havelaar *et al.*, 2015). Assuming that, if carcinogenic, AFM1 causes the same cancer as AFB1, the data for AFRE subregion can be used to estimate the CFR and DALYs/case for AFB1 in Ethiopia. Assuming this, we calculated:

$${Case-fatality ratio}_{AFM1}= \frac{AFRE total AFB1 mortality}{AFRE total AFB1 incidence}$$

$${Mortality}_{AFM1}= {Incidence}_{AFM1}*{Case-fatality ratio}_{AFM1}$$

$${Mortality rate}_{AFM1}= \frac{{Mortality}_{AFM1}}{ET population size}*100,000$$

$${DALYs}_{AFM1}= {Incidence per 100,000}_{AFM1}*AFRE DALYs/case$$

$${DALYs rate}_{AFM1}= \frac{{DALYs}_{AFM1}}{ET population size}*100,000$$

Mortality and DALYs were estimated using the approximate approach described above, assuming lognormal distributions. CFR was calculated using a beta distribution. Input data are summarized in Table 8. Best estimates and uncertainty estimates for risk metrics are provided in Table 9, using 2010 data.

| **Table 8. Aflatoxin M1 input data** | | | |
| --- | --- | --- | --- |
| **Metric** | **Lower** | **Middle** | **Upper** |
| AFB1 incidence (WHO FERG, 2015) | 180 | 430 | 1000 |
| AFB1 mortality (WHO FERG, 2015) | 160 | 380 | 910 |
| DALYs per case (WHO FERG, 2015) | 31 | 33 | 35 |

| **Table 9. Aflatoxin M1 foodborne estimates by risk metric** | | | |
| --- | --- | --- | --- |
| **Metric** | **Lower** | **Middle** | **Upper** |
| Incidence (Cases per year) | 0.69 | 2.2 | 6.9 |
| Incidence per 100000 | 0.00079 | 0.0025 | 0.0079 |
| Mortality (deaths per year) | 0.60 | 2.1 | 6.00 |
| Mortality per 100000 | 0.00069 | 0.00233 | 0.0069 |
| Case Fatality Ratio | 0.84 | 0.87 | 0.90 |
| DALYs | 23 | 78 | 230 |
| DALYs per 100000 | 0.026 | 0.089 | 0.26 |
| DALYs per case | 31 | 33 | 35 |

**References**

European Commission (EU). Commission Regulation (EC) No. 1881/2006 of 19 December 2006. Official Journal of European Union, L364/5-24.

Hoddinott J, Headey D, Dereje M. Cows, missing milk markets, and nutrition in rural Ethiopia. The Journal of Development Studies 2015;51(8):958–75.

Saha Turna, Nikita, Arie Havelaar, Adegbola Adesogan and Felicia Wu. 2022. “Aflatoxin M1 in Milk Does Not Contribute Substantially to Global Liver Cancer Incidence.” *The American Journal of Clinical Nutrition*.

Saha Turna, Nikita, and Felicia Wu. 2021. “Aflatoxin M1 in Milk: A Global Occurrence, Intake, & Exposure Assessment.” *Trends in Food Science & Technology* 110 (April): 183–92. <https://doi.org/10.1016/j.tifs.2021.01.093>.

### ***Bacillus anthracis***

*Bacillus anthracis* is a gram-positive bacterium that produces extremely hardy spores that can survive in the environment for decades and is naturally found in soils. Livestock and other animals, particularly ruminants, around the world can be infected. People get infected with anthrax when spores get into the body and are activated in the body, producing toxins (poisons) that cause severe illness.

**Rationale for Inclusion**

Anthrax was ranked the #3 priority zoonotic disease in Ethiopia in a risk ranking exercise conducted by the US CDC, EPHI and various other organizations (Pieracci *et al.*, 2016). Skin and intestinal anthrax are reported in various regions where many outbreaks in livestock (and wildlife) continue to occur. Human consumption of animal products or tissues harvested from an infected animal can lead to gastrointestinal anthrax. As such, all gastrointestinal cases are assumed to be foodborne.

**Available data**

No estimates of the incidence, deaths, mortality or DALYs of (foodborne) anthrax are available for Ethiopia.

There were an estimated 5,197 cases and 86 deaths from human anthrax in Ethiopia between 2009 – 2013 using retrospective data from EPHI (Bahiru *et al.*, 2016). No estimates were provided for the different forms of anthrax (cutaneous, inhalation, gastrointestinal). Two recent papers [Blackburn *et al.*, 2021; Ashenefe Wassie *et al.*, 2022] refer to anthrax CFR related to cutaneous human exposure only and were not considered representative for foodborne anthrax in Ethiopia; data on exposure via (food) ingestion and CFR were not reported in these papers and where not found elsewhere.

To estimate incidence of foodborne anthrax in Ethiopia, we used the Bahiru *et al.* (2016) estimate of overall anthrax as the midpoint of the distribution. We then used a Bayesian approach to obtain lower and upper estimates. We assumed that the number of cases were Gamma distributed with shape parameter of 5,197 (number of cases) and a scale parameter of 1/5 (5 years of observation). We then ran 100,000 simulations to derive a distribution for the incidence. We derived the case-fatality ratio using a similar approach. We fit a beta distribution using the number of cases and deaths reported by Bahiru et al. (2016) to obtain the lower, middle and upper estimates.

The number of foodborne cases of anthrax in Ethiopia can be estimated by multiplying the distribution of the incidence of all anthrax cases by the proportion that are gastrointestinal. Because anthrax is more common in low- and middle-income countries where reporting is not common, the true incidence of GI anthrax is unknown (Maddah et al., 2013). We, therefore, used expert feedback to estimate that 5% (1% lower, 20% upper) of all anthrax cases are foodborne. Assuming this, we calculated:

$${Incidence}_{Foodborne anthrax}={Incidence}_{All anthrax}*Proportion foodborne$$

$${Incidence rate}_{Foodborne anthrax}= \frac{{Incidence}_{Foodborne anthrax}}{ET population size}*100,000$$

$${Mortality}_{Foodborne anthrax}= {Incidence}_{Foodborne anthrax}*{Case-fatality ratio}_{All anthrax}$$

$${Mortality rate}_{Foodborne anthrax}= \frac{{Mortality}_{Foodborne anthrax}}{ET population size}*100,000$$

Total DALYs were calculated as the Years of Life Lost (YLL) since anthrax is a deadly disease and rarely contributes to short term disability. To be consistent with FERG, we assumed a life expectancy of 90 years. Based on expert input and team review, we estimated the average age of onset is 50 years with 30 years as a lower estimate and 70 years as an upper estimate. Assuming this, we calculated:

$$DALYs= {YLL}_{Foodborne anthrax}= {Mortality}_{Foodborne anthrax}*(Life expectancy-Age at onset)$$

$${DALYs rate}_{Foodborne anthrax}= \frac{{Years of Life Lost}_{Foodborne anthrax}}{ET population size}*100,000$$

$${DALYs per case}_{Foodborne anthrax}= \frac{{DALYs}_{Foodborne anthrax}}{{Incidence}_{Foodborne anthrax}}$$

Incidence, mortality, DALYs and DALYs per case were estimated using the approximate approach described above, assuming lognormal distributions. Input data are summarized in Table 10. Best estimates and uncertainty estimates for risk metrics are provided in Table 11, using 2010 data.

| **Table 10. Anthrax input data** | | | |
| --- | --- | --- | --- |
| **Metric** | **Lower** | **Middle** | **Upper** |
| Incidence of all anthrax (Bahiru et al., 2016) | 1000 | 1000 | 1100 |
| Proportion that are foodborne (assumed) | 0.01 | 0.05 | 0.2 |
| Case-fatality ratio (Bahiru et al, 2016 with estimation) | 0.013 | 0.017 | 0.02 |
| Age of onset (assumed) | 30 | 50 | 70 |

| **Table 11. Anthrax foodborne estimates by risk metric** | | | |
| --- | --- | --- | --- |
| **Metric** | **Lower** | **Middle** | **Upper** |
| Incidence (Cases per year) | 10 | 53 | 210 |
| Incidence per 100000 | 0.012 | 0.06 | 0.24 |
| Mortality (deaths per year) | 0.17 | 0.88 | 3.5 |
| Mortality per 100000 | 0.00019 | 0.001 | 0.004 |
| Case Fatality Ratio | 0.013 | 0.017 | 0.02 |
| DALYs | 5.3 | 31 | 130 |
| DALYs per 100000 | 0.0061 | 0.035 | 0.15 |
| DALYs per case | 0.064 | 0.75 | 5.2 |

**References**

Ashenefe Wassie B, Fantaw S, Mekonene Y, Teshale AM, Yitagesu Y, et al. (2022) First PCR Confirmed anthrax outbreaks in Ethiopia—Amhara region, 2018–2019. PLOS Neglected Tropical Diseases 16(2): e0010181. <https://doi.org/10.1371/journal.pntd.0010181>

Blackburn, J. K., Kenu, E., Asiedu-Bekoe, F., Sarkodie, B., Kracalik, I. T., Bower, W. A....Traxler, R. M. (2021). High Case-Fatality Rate for Human Anthrax, Northern Ghana, 2005–2016. *Emerging Infectious Diseases*, *27*(4), 1216-1219. https://doi.org/10.3201/eid2704.204496.

Bahiru et al., “Human and Animal Anthrax in Ethiopia: A Retrospective Record Review 2009-2013,” Ethiopian Veterinary Journal 20, no. 2 (2016): 76–85, https://doi.org/10.4314/evj.v20i2.6.

Maddah, G., Abdollahi, A., & Katebi, M. (2013). Gastrointestinal anthrax: Clinical experience in 5 cases. *Caspian Journal of Internal Medicine*, *4*(2), 672–676. <https://www.ncbi.nlm.nih.gov/pmc/articles/PMC3755822/>

Pieracci EG, Hall AJ, Gharpure R, Haile A, Walelign E, Deressa A, Bahiru G, Kibebe M, Walke H, Belay E. Prioritizing zoonotic diseases in Ethiopia using a one health approach. One Health. 2016 Dec;2:131-135. doi: 10.1016/j.onehlt.2016.09.001. PMID: 28220151; PMCID: PMC5315415.

### ***Clostridium botulinum***

*C. botulinum* is an anaerobic spore-forming Gram-positive bacterium that can produce toxins when growing in foods. Foods that are inappropriately processed or preserved through home canning or home-bottling are most likely to provide an environment where *C. botulinum* can grow and produce toxins (WHO, 2022). The disease caused by exposure to these toxins is called botulism.

**Rationale for inclusion**

*C. botulinum* was recommended for inclusion by participants at the TARTARE Scoping Workshop held in March 2020. All botulism cases are assumed to be foodborne.

**Available data**

No estimates of the incidence, deaths, mortality or DALYs of botulism are available for Ethiopia.

WHO FERG provided estimates for high-income countries only and estimated that, in these countries, there are 475 (range 183 – 999) cases of botulism, 24 (7 – 65) deaths, and 1,036 (299 – 2,805) DALYs (WHO, 2015). The incidence was based on data from Canada: 0.04 per 100,000 (range 0.02 – 0.08) (Kirk *et al.*, 2015). For severe botulism, the case-fatality ratio was estimated to be 15% (range 5 – 25). It was assumed that 35% of cases (range 20 – 50) result in severe botulism. No mortality was associated with mild botulism. Since data were only available for high-income countries, these estimates were not included in the final WHO FERG global estimates.

Assuming that the incidence in Ethiopia is comparable to the incidence in high-income countries, we calculated:

$${Incidence}_{botulism}={Incidence rate}_{Botulism}*ET population size$$

$${Severe Incidence}_{botulism}= {Incidence}_{botulism}*Proportion resulting in severe botulism$$

To estimate mortality and mortality rate for Ethiopia, we assumed the case-fatality ratio for Ethiopia was twice the case-fatality ratio estimated by WHO FERG since antibodies may not be readily available in Ethiopia. Assuming this, we calculated:

$${Mortality}_{botulism}= {Severe Incidence}_{botulism}*2* {Case-fatality ratio}_{botulism}$$

$${Mortality rate}_{botulism}= \frac{{Mortality}_{botulism}}{ET population size}*100,000$$

To estimate DALYs for Ethiopia, we assumed the DALYs/case estimate for Ethiopia is twice the WHO FERG DALYs/case estimate. We then calculated:

$${DALYs/case}_{botulism}= \frac{{Total Global DALYs}_{botulism}}{{Total Global Incidence}_{botulism}}$$

$${DALYs}_{botulism}= {Incidence}_{botulism}*{DALYs/case}_{botulism}$$

$${DALYs rate}_{botulism}= \frac{{DALYs}_{botulism}}{ET population size}*100,000$$

Mortality, DALYs and DALYs per case were estimated using the approximate approach described above, assuming lognormal distributions. Input data are summarized in Table 12. Best estimates and uncertainty estimates for risk metrics are provided in Table 13, using 2010 data.

| **Table 12. Botulism input data** | | | |
| --- | --- | --- | --- |
| **Metric** | **Lower** | **Middle** | **Upper** |
| Incidence in high-income countries (WHO FERG, 2015) | 180 | 480 | 990 |
| Incidence per 100,000 (WHO FERG, 2015) | 0.02 | 0.04 | 0.08 |
| Proportion resulting in severe botulism (WHO FERG, 2015) | 0.2 | 0.35 | 0.5 |
| ET severe incidence (calculated) | 4.8 | 12 | 25 |
| Case-fatality ratio (WHO FERG, 2015 with adjustments) | 0.1 | 0.3 | 0.5 |
| Total global DALYs (WHO FERG, 2015) | 300 | 1000 | 2800 |

| **Table 13. Botulism foodborne estimates by risk metric** | | | |
| --- | --- | --- | --- |
| **Metric** | **Lower** | **Middle** | **Upper** |
| Incidence (Cases per year) | 18 | 35 | 70 |
| Incidence per 100000 | 0.02 | 0.04 | 0.08 |
| Mortality (deaths per year) | 0.78 | 2.7 | 7.9 |
| Mortality per 100000 | 0.00089 | 0.0031 | 0.009 |
| Case Fatality Ratio | 0.1 | 0.3 | 0.5 |
| DALYs | 16 | 87 | 360 |
| DALYs per 100000 | 0.018 | 0.099 | 0.41 |
| DALYs per case | 0.53 | 2.4 | 8.7 |

**References**

World Health Organization, ed., *Botulism*. World Health Organization (Geneva, Switzerland, 2018). Retrieved from <https://www.who.int/news-room/fact-sheets/detail/botulism>

Kirk, M., et al., “World Health Organization Estimates of the Global and Regional Disease Burden of 22 Foodborne Bacterial, Protozoal, and Viral Diseases, 2010: A Data Synthesis,” *PLOS Medicine* 12, no. 12 (December 3, 2015): e1001921, <https://doi.org/10.1371/journal.pmed.1001921>

World Health Organization, ed., *WHO Estimates of the Global Burden of Foodborne Diseases* (Geneva, Switzerland: World Health Organization, 2015).

### ***Lathyrus sativa***

*Lathyrus sativus*, also known as grasspea, is a leguminous crop that is commonly grown and consumed in part of Northern India and Ethiopia. The neuroexcitatory compound β-ODAP (β-N-oxalyl-l-α,β diaminopropionic acid) is essential to *L. sativus* biosynthesis and therefore present in the crop when it is consumed (Yan *et al.,* 2006). When consumed in large quantities, it can lead to paralysis, often of the lower limbs; this disease is known as neurolathyrism. In 1998, it was reported that neurolathyrism was more common when grass pea accounted for greater than 30% of the caloric intake sustained over a period of 3-4 months (Singh and Rao, 2013).

**Rationale for inclusion**

Growth of grasspea has been well documented in Ethiopia for most of history, and its ability to survive through drought makes it a reliable crop for farmers (Tekle-Haimanot *et al.*, 1993). There have been three reported epidemics of neurolathyrism in Ethiopia and Eritrea in the past 50 years (Getahun *et al.*, 1999). In Ethiopia, epidemics of lathyrism were observed in the 1970s, 1980s and 1990s following periods of drought and overconsumption of grasspea.

**Available data**

A survey of northwestern Ethiopia found an estimated disease prevalence of 0.6% to 2.9% with an estimated annual incidence of 1.7 per 10,000 population (Haimanot *et al.*, 1990). Getahun et al. (1999) noted that the incidence rates were an order of magnitude higher during pandemic episodes in Northwestern Ethiopia between 1996-1998. A survey of one million households in northwest and central Ethiopia found prevalence rates ranging from 1 per 10,000 to 7.5 per 1,000 for lathyrism (Haimanot *et al.*, 1993). Following a 1997 – 1999 epidemic in northeastern Ethiopia, a survey of 2,987 individuals from 589 households found the prevalence of neurolathyrism to be 2.38% (1.83% - 2.93%) with a median age of onset of 11 years (3 – 44) (Getahun *et al.*, 2002). Another survey of 118 households in the Amharic region of Ethiopia found at least a third of households had at least one member with neurolathyrism and the mean age of onset was 15.08 years (SD: 11.26) (Fikre *et al.*, 2009).

Since the estimated incidence of 1.7 per 10,000 followed a drought, we assumed this estimate would be a worst-case scenario. We estimated the midpoint by dividing by 10 and estimated the lower level by dividing by 10 again. We then calculated:

$${Incidence}_{neurolathyrism}={Incidence rate}_{neurolathyrism}*ET population size$$

It is assumed that there are no deaths associated with *Lathyrus sativa* and DALYs only result from Years Lived in Disability (YLD). We also assumed that neurolathyrism is a permanent condition. To be consistent with FERG, we assumed a life expectancy of 90 years. Based on expert input and team review, we estimated the average age of onset to be 20 years and assumed the upper and lower bounds to be 5 and 40 years, respectively. Disability weights are not available for neurolathyrism but are available for konzo (disability weight = 0.377), which produces similar health outcomes. Based on these assumptions, we calculated:

$${DALYs= YLD}_{neurolathyrism}= {Incidence}_{neurolathyrism}*\left( Life expectancy-Age at onset \right)*0.377$$

$${DALYs rate}_{neurolathyrism}= \frac{{YLD}_{neurolathyrism}}{ET population size}*100,000$$

$${DALYs per case}_{neurolathyrism}= \frac{{DALYs}_{neurolathyrism}}{{Incidence}_{neurolathyrism}}$$

DALYs and DALYs per case were estimated using the approximate approach described above, assuming lognormal distributions. Input data are summarized in Table 14. Best estimates and uncertainty estimates for risk metrics are provided in Table 15, using 2010 data.

| **Table 14. Lathyrism input data** | | | |
| --- | --- | --- | --- |
| **Metric** | **Lower** | **Middle** | **Upper** |
| Incidence per 10,000 (Getahun et al., 1999 with adjustments) | 0.017 | 0.17 | 1.7 |
| Age of onset (estimated) | 5 | 20 | 40 |
| Disability weight | 0.00377 | 0.0377 | 0.377 |

| **Table 15. Lathyrism foodborne estimates by risk metric** | | | |
| --- | --- | --- | --- |
| **Metric** | **Lower** | **Middle** | **Upper** |
| Incidence (Cases per year) | 150 | 1500 | 15000 |
| Incidence per 100000 | 0.17 | 1.7 | 17 |
| Mortality (deaths per year) | 0 | 0 | 0 |
| Mortality per 100000 | 0 | 0 | 0 |
| Case Fatality Ratio | 0 | 0 | 0 |
| DALYs | 140 | 6700 | 96000 |
| DALYs per 100000 | 0.16 | 7.6 | 110 |
| DALYs per case | 0.245 | 3.3 | 25 |

**References**

A. Fikre, M. Van Moorhem, S. Ahmed, F. Lambein, G. Gheysen, Studies on neurolathyrism in Ethiopia: Dietary habits, perception of risks and prevention, Food and Chemical Toxicology, Volume 49, Issue 3, 2011, Pages 678-684, ISSN 0278-6915, <https://doi.org/10.1016/j.fct.2010.09.035>.

Getahun, A. Mekonnen, R. Tekle-Haimanot, and F. Lambein, “Epidemic of neurolathyrism in Ethiopia,” *The Lancet*, vol. 354, no. 9175, pp. 306–307, Jul. 1999, doi: 10.1016/S0140-6736(99)02532-5.

Getahun H, Lambein F, Vanhoorne M, Van der Stuyft P. Pattern and associated factors of the neurolathyrism epidemic in Ethiopia. Trop Med Int Health. 2002 Feb;7(2):118-24. Doi: 10.1046/j.1365-3156.2002.00836.x. PMID: 11841701.

Haimanot RT, Kidane Y, Wuhib E, Kalissa A, Alemu T, Zein ZA, Spencer PS. Lathyrism in rural northwestern Ethiopia: a highly prevalent neurotoxic disorder. Int J Epidemiol. 1990 Sep;19(3):664-72. Doi: 10.1093/ije/19.3.664. PMID: 2262262.

Haimanot RT, Kidane Y, Wuhib E, Kassina A, Endeshaw Y, Alemu T, Spencer PS. The epidemiology of lathyrism in north and central Ethiopia. Ethiop Med J. 1993 Jan;31(1):15-24. Erratum in: Ethiop Med J 1993 Apr;31(2):155-6. PMID: 8436097.

S. S. Singh and S. L. N. Rao, “Lessons from neurolathyrism: A disease of the past & the future of *Lathyrus sativus* (Khesari dal),” *Indian J. Med. Res.*, vol. 138, no. 1, pp. 32–37, Jul. 2013, Accessed: May 15, 2020. [Online]. Available: <https://www.ncbi.nlm.nih.gov/pmc/articles/PMC3767245/>

Haimanot, T.R., B.M. Abegaz, E. Wuhib, A. Kassina, Y. Kidane, N. Kebede,

T. Alemu and P.S. Spencer “Pattern of *Lathyrus sativus* (grass pea) consumption and beta-N-oxalyl-α-β-diaminoproprionic acid (β-ODAP) content of food samples in the lathyrism endemic region of northwest ethiopia,” *Nutr. Res.*, vol. 13, no. 10, pp. 1113–1126, Oct. 1993, doi: 10.1016/S0271-5317(05)80736-5.

Z.-Y. Yan *et al.*, “*Lathyrus sativus* (grass pea) and its neurotoxin ODAP,” *Phytochemistry*, vol. 67, no. 2, pp. 107–121, Jan. 2006, doi: 10.1016/j.phytochem.2005.10.022.

### **Rift Valley Fever**

Rift Valley fever virus (RVF) can be transmitted to humans or livestock by mosquitoes or through direct contact with contaminated bodily fluids and tissues of infected animals. This direct contact can occur during slaughter or butchering, while caring for sick animals, during veterinary procedures, and when consuming raw or undercooked animal products. There is some evidence that humans may become infected with RVF by ingesting the unpasteurized or uncooked milk of infected animals. Because foodborne transmission is uncommon, there is little literature characterizing this exposure and associated outcomes.

**Rationale for Inclusion**

As of 2016, Ethiopia had not reported any outbreaks of RVF, but geographical analyses identified areas in the country as highly vulnerable to an RVF outbreak (Kimani *et al.*, 2016; Tran *et al.*, 2016).

**Available data**

No estimates of the incidence, deaths, mortality or DALYs of foodborne RVF are available for Ethiopia.

As of 2016, no cases of human RVF had been reported in Ethiopia so an estimate of 2 (range 0.1 – 5) cases per year was used to generate burden estimates. Several studies have examined the seroprevalence in humans and animals (Ikegami *et al.*, 2011; Grossi-Soyster *et al.*, 2019; Asebe *et al.*, 2020; Ibrahim *et al.*, 2021) but these estimates are likely to be much higher than actual incidence, as antibodies may be long-lived.

RVF is not generally transmitted through food, but the possibility is recognized in Grossi-Soyster et al. (2019). Estimates of 1% (0.1%, 5%) were used to estimate the number of foodborne RVF cases in Ethiopia (Grossi-Soyster *et al.*, 2019).

$${Incidence}_{foodborne RVF}= {Incidence}_{RVF}*proportion that are foodborne$$

$${Incidence rate}_{RVF}= \frac{{Incidence}_{foodborne RVF}}{ET population size}*100,000$$

Mortality (deaths per year) and mortality rate (per 100,000) were estimated assuming a case fatality rate of 1% with upper and lower bounds of 0.3% and 2% respectively. These estimates were derived using a Beta distribution that approximately fit the range of 0.5% to 2% cited by Javelle *et al.* (2020).

$${Mortality}_{foodborne RVF}= {Incidence}_{foodborne RVF}*{Case-fatality ratio}_{RVF}$$

$${Mortality rate}_{foodborne RVF}= \frac{{Mortality}_{foodborne RVF}}{ET population size}*100,000$$

To estimate DALYs for Ethiopia, we assumed 0.029 DALYs/case (Kimani *et al.*, 2016) for the midpoint. We multiplied/divided this by 10 to obtain upper and lower levels. We then calculated:

$${DALYs}_{foodborne RVF}= {Incidence}_{foodborne RVF}*{DALYs per case}_{RVF}$$

$${DALYs rate}_{foodborne RVF}= \frac{{DALYs}_{foodborne RVF}}{ET population size}*100,000$$

$${DALYs per case}_{foodborne RVF}= \frac{{DALYs}_{foodborne RVF}}{{Incidence}_{foodborne RVF}}$$

Incidence, mortality, DALYs and DALYs per case were estimated using the approximate approach described above, assuming lognormal distributions. Input data are summarized in Table 16. Best estimates and uncertainty estimates for risk metrics are provided in Table 17, using 2010 data.

| **Table 16. Rift Valley Fever input data** | | | |
| --- | --- | --- | --- |
| **Metric** | **Lower** | **Middle** | **Upper** |
| Total RVF incidence | 0.1 | 2 | 5 |
| Proportion Foodborne (Grossi-Soyster et al., 2019) | 0.001 | 0.01 | 0.05 |
| Case-fatality ratio (Javelle et al., 2020 with adjustments) | 0.003 | 0.01 | 0.02 |
| DALYs per Case (Kimani et al., 2016 with adjustments) | 0.0029 | 0.029 | 0.29 |

| **Table 17. Rift Valley Fever foodborne estimates by risk metric** | | | |
| --- | --- | --- | --- |
| **Metric** | **Lower** | **Middle** | **Upper** |
| Incidence (Cases per year) | 0.00031 | 0.0077 | 0.079 |
| Incidence per 100000 | 0.00000036 | 0.0000088 | 0.000091 |
| Mortality (deaths per year) | 0.0000021 | 0.000063 | 0.00072 |
| Mortality per 100000 | 0.0000000024 | 0.000000072 | 0.00000082 |
| Case Fatality Ratio | 0.003 | 0.01 | 0.02 |
| DALYs | 0.000004 | 0.0003 | 0.0053 |
| DALYs per 100000 | 0.0000000045 | 0.00000034 | 0.000006 |
| DALYs per case | 0.00031 | 0.093 | 2.7 |

**References**

Asebe, G.; Mamo, G.; Michlmayr, D.; Abegaz, W. E.; Endale, A.; Medhin, G.; Larrick, J. W.; Legesse, M.Seroprevalence of Rift Valley Fever and West Nile Fever in Cattle in Gambella Region, South West Ethiopia. *Vet. Med. Res. Rep.* 2020, *11*, 119–130. <https://doi.org/10.2147/VMRR.S278867>.

Grossi-Soyster, E. N.; Lee, J.; King, C. H.; LaBeaud, A. D. The Influence of Raw Milk Exposures on Rift Valley Fever Virus Transmission. *PLoS Negl. Trop. Dis.* 2019, *13* (3), e0007258. <https://doi.org/10.1371/journal.pntd.0007258>.

Ibrahim, M.; Schelling, E.; Zinsstag, J.; Hattendorf, J.; Andargie, E.; Tschopp, R. Sero-Prevalence of Brucellosis, Q-Fever and Rift Valley Fever in Humans and Livestock in Somali Region, Ethiopia. *PLoS Negl. Trop. Dis.* 2021, *15* (1), e0008100. <https://doi.org/10.1371/journal.pntd.0008100>.

Ikegami, T.; Makino, S. The Pathogenesis of Rift Valley Fever. *Viruses* 2011, *3* (5), 493–519. <https://doi.org/10.3390/v3050493>

Javelle, E.; Lesueur, A.; Pommier de Santi, V.; de Laval, F.; Lefebvre, T.; Holweck, G.; Durand, G. A.; Leparc-Goffart, I.; Texier, G.; Simon, F. The Challenging Management of Rift Valley Fever in Humans: Literature Review of the Clinical Disease and Algorithm Proposal. *Ann. Clin. Microbiol. Antimicrob.* 2020, *19*. <https://doi.org/10.1186/s12941-020-0346-5>.

Kimani, T.; Schelling, E.; Bett, B.; Ngigi, M.; Randolph, T.; Fuhrimann, S. Public Health Benefits from Livestock Rift Valley Fever Control: A Simulation of Two Epidemics in Kenya. *Ecohealth* 2016, *13* (4), 729–742. <https://doi.org/10.1007/s10393-016-1192-y>.

Tran, A.; Trevennec, C.; Lutwama, J.; Sserugga, J.; Gély, M.; Pittiglio, C.; Pinto, J.; Chevalier, V. Development and Assessment of a Geographic Knowledge-Based Model for Mapping Suitable Areas for Rift Valley Fever Transmission in Eastern Africa. *PLoS Negl. Trop. Dis.* 2016, *10* (9). <https://doi.org/10.1371/journal.pntd.0004999>.

### **Rotavirus**

Rotavirus is transmitted via the fecal-oral route. Person-to-person spread is the most common mode of transmission, though foodborne outbreaks are associated with rotavirus. Contamination of food with fecal material can occur anywhere along the farm-to-fork continuum, including viral contamination at the source. Rotavirus vaccine coverage is estimated to be 56% nationally but varies greatly by region (Geweniger and Abbas, 2020).

**Rationale for inclusion**

Rotavirus is a well-studied gastrointestinal illness characterized by fever, dehydration, watery diarrhea, and abdominal pain that typically appear two days after exposure and last 3 to 8 days (CDC, 2019). The disease primarily affects infants; however, adults how are sickened as well but generally have more mild illnesses. In 2015, an estimated 2587.9 deaths were attributed to rotavirus in children below 5 years of age in Ethiopia (GBD, 2015).

**Available data**

No estimates of the incidence, deaths, mortality or DALYs of foodborne rotavirus are available for Ethiopia.

Starting in 1990, the Institute for Health Metrics and Evaluation (IHME) Global Burden of Disease (GBD) has quantified the health loss from more than 350 diseases and injuries in 195 countries each year, including Ethiopia. Incidence for diarrheal disease and mortality and DALYs associated with diarrheal rotavirus were obtained for Ethiopia for 2010 (IHME, 2022). The proportion of diarrheal DALYs attributable to rotavirus in Ethiopia in 2010 were also obtained (IHME, 2022). We assumed that the proportion of total cases that are attributable to rotavirus is the same as the proportion of total DALYs attributable to rotavirus.

We also assumed the proportion of rotavirus cases that are foodborne to be 13% (range 13 – 28) based on an expert elicitation conducted in The Netherlands in 2008 (Havelaar et al., 2008). We then adjusted the lower bound to 5% based on expert input and team review.

To estimate incidence of foodborne rotavirus in Ethiopia, we calculated:

$${Incidence}_{diarrhea}={Incidence rate}_{diarrhea}*ET population size$$

$${Incidence}_{rotavirus}= {Incidence}_{diarrhea}*proportion of DALYs attributable to rotavirus$$

$${Incidence}_{foodborne rotavirus}= {Incidence}_{rotavirus}*proportion that are foodborne$$

$${Incidence rate}_{Foodborne rotovirus}= \frac{{Incidence}_{Foodborne rotavirus}}{ET population size}*100,000$$

To estimate mortality (deaths per year) and mortality rate (per 100,000) for foodborne rotavirus in Ethiopia, we calculated:

$${Mortality}_{foodborne rotavirus}= {Mortality}_{rotavirus}*proportion that are foodborne$$

$${Mortality rate}_{foodborne rotavirus}= \frac{{Mortality}_{foodborne rotavirus}}{ET population size}*100,000$$

$${Case fatality ratio}_{foodborne rotavirus}= \frac{{Mortality}_{foodborne rotavirus}}{{Incidence}_{foodborne rotavirus}}$$

To estimate total DALYs per 100,000 for foodborne rotavirus in Ethiopia, we calculated:

$$Total {DALYs}_{foodborne rotavirus}= {Total DALYs}_{rotavirus}*proportion that are foodborne$$

$${DALYs rate}_{foodborne rotavirus}= \frac{{DALYs}_{foodborne rotavirus}}{ET population size}*100,000$$

$${DALYs per case}_{foodborne rotavirus}= \frac{{DALYs}_{foodborne rotavirus}}{{Incidence}_{foodborne rotavirus}}$$

Incidence, mortality and DALYs were estimated for foodborne rotavirus using the approximate approach described above, assuming lognormal distributions. The same approach was used to estimate case fatality ratio and DALYs per case. Input data are summarized in Table 18. Best estimates and uncertainty estimates for risk metrics are provided in Table 19, using 2010 data.

| **Table 18. Rotavirus input data** | | | |
| --- | --- | --- | --- |
| **Metric** | **Lower** | **Middle** | **Upper** |
| ET diarrheal incidence (IHME)* | 88,000,000 | 97,000,000 | 110,000,000 |
| Proportion diarrheal DALYs due to rotavirus (IHME) | 0.053 | 0.12 | 0.2 |
| ET rotavirus incidence (calculated) | 5,000,000 | 10,000,000 | 20,000,000 |
| ET rotavirus mortality (IHME) | 2,000 | 5,800 | 12,000 |
| ET rotavirus DALYs (IHME) | 170,000 | 490,000 | 1,100,000 |
| Proportion foodborne (Havelaar et al., 2008 with adjustment) | 0.05 | 0.13 | 0.28 |

* Incidence exceeds population size due to multiple occurrences of diarrhea in a single individual

| **Table 19. Rotavirus foodborne estimates by risk metric** | | | |
| --- | --- | --- | --- |
| **Metric** | **Lower** | **Middle** | **Upper** |
| Incidence (Cases per year) | 390,000 | 1,300,000 | 3,500,000 |
| Incidence per 100000 | 450 | 1,400 | 4,100 |
| Mortality (deaths per year) | 170 | 640 | 2,100 |
| Mortality per 100000 | 0.19 | 0.74 | 2.4 |
| Case Fatality Ratio | 0.00047 | 0.00051 | 0.00055 |
| DALYs | 14,000 | 55,000 | 180,000 |
| DALYs per 100000 | 17 | 63 | 200 |
| DALYs per case | 0.0081 | 0.05 | 0.23 |

**References**

Center for Disease Control, ed., *Rotavirus*, Department of Human Health Services Center for Disease Control (Atlanta, Georgia, 2019), Retrieved from <https://www.cdc.gov/rotavirus/index.html>.

Anne Geweniger and Kaja M. Abbas, “Childhood Vaccination Coverage and Equity Impact in Ethiopia by Socioeconomic, Geographic, Maternal, and Child Characteristics,” *Vaccine* 38, no. 20 (April 29, 2020): 3627–38, <https://doi.org/10.1016/j.vaccine.2020.03.040>.

GBD Diarrhoeal Diseases Collaborators, “Estimates of Global, Regional, and National Morbidity, Mortality, and Aetiologies of Diarrhoeal Diseases: A Systematic Analysis for the Global Burden of Disease Study 2015,” The Lancet. Infectious Diseases 17, no. 9 (2017): 909–48, https://doi.org/10.1016/S1473-3099(17)30276-1.

Arie H. Havelaar et al., “Attribution of Foodborne Pathogens Using Structured Expert Elicitation,” Foodborne Pathogens and Disease 5, no. 5 (October 2008): 649–59, https://doi.org/10.1089/fpd.2008.0115.

Institute for Health Metrics and Evaluation (IHME). GBD Compare. Seattle, WA: IHME, University of Washington. Available from http://vizhub.healthdata.org/gbd-compare. Accessed June 11, 2022 using Year =2010, Cause= Diarrheal diseases, Etiology = Rotavirus, Location=Ethiopia, All ages, both sexes.

### ***Staphylococcus aureus***

*Staphylococcus aureus* (SA) is a gram-positive bacterium that can cause a variety of illnesses, including foodborne disease. Certain SA strains produce staphylococcal enterotoxins (SEs) that can cause illness, especially when SA levels are high. Most outbreaks are the result of poor hygiene during processing resulting in transfer of SA or SEs to the food from human skin or dairy animals with mastitis (Hennekinee *et al.*, 2010).

**Rationale for inclusion**

Exposure to SA is well established in Ethiopia. Studies have been conducted in several parts of the country investigating the prevalence of SA in dairy farms, abattoirs, and among human food handlers, but this data is not specific to the presence of SEs (Ayele et al.; 2017; Beyene et al., 2017; Beyene et al., 2019).

**Available data**

No estimates of the incidence, deaths, mortality or DALYs of SA are available for Ethiopia.

WHO FERG estimated the incidence of SA in high-income countries to be 77.3 per 100,000 (range 50.65 – 118.0) using data from Canada and the case-fatality ratio to be 0.0025% (range 0.0012 – 00.0045) using data from the United States (WHO, 2015). Since data were only available for high-income countries, these estimates were not included in the final WHO FERG global estimates. When asked about the appropriateness of these estimates for Ethiopia, experts provided conflicting feedback. We, therefore, did not adjust these estimates.

Assuming that the incidence in Ethiopia is comparable to the incidence in high-income countries, we calculated:

$${Incidence}_{SA}={Incidence rate}_{SA}*ET population size$$

To estimate mortality (deaths per year) and mortality rate (per 100,000) for Ethiopia, we assumed the case-fatality ratio for Ethiopia was twice the case-fatality ratio estimated by WHO FERG. Assuming this, we calculated:

$${Mortality}_{SA}= {Incidence}_{SA}*2* {Case-fatality ratio}_{SA}$$

$${Mortality rate}_{SA}= \frac{{Mortality}_{SA}}{ET population size}*100,000$$

$${Case fatality ratio}_{SA}= \frac{{Mortality}_{SA}}{{Incidence}_{SA}}$$

The total number of global DALYs was not calculated by WHO FERG but an accompanying paper (Kirk *et al.*, 2015) provided estimates of 1,073,339 (658,463 – 1,639,524) cases, 25 (10 – 55) deaths, and 1,575 (702 – 3,244) DALYs for high-income countries. While these global estimates were largely derived from US and European data, we estimated the DALYs/case and DALYs per 100,000 for Ethiopia from these data:

$${DALYs/case}_{SA}= \frac{{Total Global DALYs}_{SA}}{{Total Global Cases}_{SA}}$$

$${DALYs}_{SA}= {Incidence}_{SA}*{DALYs/case}_{SA}$$

$${DALYs rate}_{SA}= \frac{{DALYs}_{SA}}{ET population size}*100,000$$

Mortality, DALYs per case and DALYs were estimated using the approximate approach described above, assuming lognormal distributions. Input data are summarized in Table 20. Best estimates and uncertainty estimates for risk metrics are provided in Table 21, using 2010 data.

| **Table 20. *Staphylococcus aureus* input data** | | | |
| --- | --- | --- | --- |
| **Metric** | **Lower** | **Middle** | **Upper** |
| Incidence per 100,000 (WHO FERG, 2015) | 44,000 | 68,000 | 100,000 |
| Case-fatality ratio (WHO FERG, 2015 with adjustments) | 0.0024 | 0.005 | 0.009 |
| Global DALYs (Kirk et al., 2015) | 700 | 1,600 | 3,200 |
| Global Cases (Kirk et al., 2015) | 660,000 | 1,100,000 | 1,600,000 |

| **Table 21. *Staphylococcus aureus* foodborne estimates by risk metric** | | | |
| --- | --- | --- | --- |
| **Metric** | **Lower** | **Middle** | **Upper** |
| Incidence (Cases per year) | 44,000 | 68,000 | 100,000 |
| Incidence per 100000 | 51 | 77 | 120 |
| Mortality (deaths per year) | 140 | 330 | 690 |
| Mortality per 100000 | 0.16 | 0.37 | 0.79 |
| Case Fatality Ratio | 0.0024 | 0.005 | 0.009 |
| DALYs | 37 | 100 | 260 |
| DALYs per 100000 | 0.042 | 0.12 | 0.3 |
| DALYs per case | 0.0006 | 0.0015 | 0.0035 |

**References**

Yodit Ayele et al., “Assessment of Staphylococcus Aureus along Milk Value Chain and Its Public Health Importance in Sebeta, Central Oromia, Ethiopia,” *BMC Microbiology* 17, no. 1 (27 2017): 141, https://doi.org/10.1186/s12866-017-1048-9(Ayele et al., 2017; G. Beyene et al., 2019; T. Beyene et al., 2017)

Getenet Beyene et al., “Nasal and Hand Carriage Rate of Staphylococcus Aureus among Food Handlers Working in Jimma Town, Southwest Ethiopia,” Ethiopian Journal of Health Sciences 29, no. 5 (September 2019): 605–12, https://doi.org/10.4314/ejhs.v29i5.11.

Takele Beyene et al., “Prevalence and Antimicrobial Resistance Profile of Staphylococcus in Dairy Farms, Abattoir and Humans in Addis Ababa, Ethiopia,” *BMC Research Notes* 10, no. 1 (April 28, 2017): 171, https://doi.org/10.1186/s13104-017-2487-y;

Jacques-Antoine Hennekinne et al., “How Should Staphylococcal Food Poisoning Outbreaks Be Characterized?,” *Toxins* 2, no. 8 (August 10, 2010): 2106–16, <https://doi.org/10.3390/toxins2082106>.

Martyn D. Kirk et al., “World Health Organization Estimates of the Global and Regional Disease Burden of 22 Foodborne Bacterial, Protozoal, and Viral Diseases, 2010: A Data Synthesis,” PLOS Medicine 12, no. 12 (December 3, 2015): e1001921

World Health Organization, ed., *WHO Estimates of the Global Burden of Foodborne Diseases* (Geneva, Switzerland: World Health Organization, 2015).

### ***Taenia saginata***

*Taenia saginata* is an intestinal cestode (tapeworm) that lives in the intestines of humans as the definitive host. The larval stage occurs in tissues of cattle, as the intermediate host, and are also known as *Cysticercus bovis*. Consuming infected meat can lead to human infection.

**Rationale for Inclusion**

Cysticercosis/taeniasis was ranked the #11 priority zoonotic disease in Ethiopia in a risk ranking exercise conducted by CDC, EPHI and various other organizations (Pieracci *et al.*, 2016). The disease is not severe and no fatalities are extremely rare, but infection of beef cattle presents an economic burden because of meat inspection requirements. The European Food Safety Authority has categorized *T. saginata* a low priority for bovine meat inspection (EFSA BIOHAZ Panel, 2013).

**Available data**

A prevalence of 1.95% (95% CI 1.6-2.2%) was estimated from a recently published systematic review and meta-analysis of 44 articles reporting human intestinal parasitic infections based on stool tests in different population groups (Edilu *et al.*, 2020). Prevalence was highest in food handlers, followed by the general population, hospital patients, and school children. Note that estimates based on stool tests are considerably lower than estimates based on self-reports (Hiko and Seifu, 2019). Limited data was available for the duration of taeniasis so we assumed a midpoint of 3 years and multiplied/divided by 10 to obtain upper/lower bounds. We then calculated:

$${Incidence}_{taeniasis}= \frac{{Prevalence}_{taeniasis}}{{Duration}_{taeniasis}}*ET population size$$

$${Incidence rate}_{taeniasis}= \frac{{Total Cases}_{taeniasis}}{ET population size}*100,000$$

It is assumed that there are no deaths associated with *T. saginata* and DALYs only result from Years Lived with Disability (YLD). Not all cases of taeniasis are symptomatic; an estimated 35% feel abdominal pain and/or nausea (no uncertainty range was provided) (Flisser *et al.*, 2011). Disability weights were obtained from the Global Burden of Disease study with a best estimate of 0.011 (mild-abdomino-pelvic problems), a lower estimate of 0.006 (mild nausea) and an upper estimate of 0.074 (mild diarrhea) (Salomon *et al.*, 2015). We then calculated:

$${Symptomatic Incidence}_{taeniasis}= {Incidence}_{taeniasis}*proportion symptomatic$$

$${DALYs=YLD}_{taeniasis}= {Symptomatic Incidence}_{taeniasis}*{Duration}_{taeniasis}* Disability weight$$

$${DALYs rate}_{taeniasis}= \frac{{YLD}_{taeniasis}}{ET population size}*100,000$$

$${DALYs per case}_{taeniasis}= \frac{{DALYs}_{taeniasis}}{{Incidence}_{taeniasis}}$$

Incidence, symptomatic incidence, DALYs and DALYs per case were estimated using the approximate approach described above, assuming lognormal distributions. Input data are summarized in Table 22. Best estimates and uncertainty estimates for risk metrics are provided in Table 23, using 2010 data.

| **Table 22. *Taenia saginata* input data** | | | |
| --- | --- | --- | --- |
| **Metric** | **Lower** | **Middle** | **Upper** |
| Prevalence (Edilu et al., 2020) | 0.016 | 0.019 | 0.022 |
| Duration of taeniasis in years (assumed) | 0.3 | 3 | 30 |
| Proportion symptomatic (Flisser et al.,) | 0.35 | 0.35 | 0.35 |
| Symptomatic incidence (calculated) | 19,000 | 260,000 | 1,900,000 |
| Disability weights (Salomon et al., 2015) | 0.006 | 0.011 | 0.04 |
| YLDs (calculated) | 81 | 31,000 | 990,000 |

| **Table 23. *Taenia saginata* foodborne estimates by risk metric** | | | |
| --- | --- | --- | --- |
| **Metric** | **Lower** | **Middle** | **Upper** |
| Incidence (Cases per year) | 55,000 | 740,000 | 5,500,000 |
| Incidence per 100000 | 62 | 850 | 6,300 |
| Mortality (deaths per year) | 0 | 0 | 0 |
| Mortality per 100000 | 0 | 0 | 0 |
| Case Fatality Ratio | 0 | 0 | 0 |
| DALYs | 81 | 31,000 | 990,000 |
| DALYs per 100000 | 0.092 | 36 | 1100 |
| DALYs per case | 0.000086 | 0.077 | 3.1 |

**References**

EFSA BIOHAZ Panel (EFSA Panel on Biological Hazards), 2013. Scientific Opinion on the public health hazards to be covered by inspection of meat (bovine animals). EFSA Journal 2013;11(6):3266, 261 pp. doi:10.2903/j.efsa.2013.3266

Flisser A, Craig PS, Ito A. Cysticercosis and taeniosis: Taenia solium, Taenia saginata and Taenia asiatica. In: Palmer SR, Soulsby L, Torgerson PR, Brown DWG, editors. Oxford Textbook of Zoonoses, Biology, Clinical Practice and Public Health Control. Oxford, UK: Oxford University Press; 2011. pp. 627–644.

Jorga Edilu, Inge Van Damme, Bizunesh Mideksa, and Sarah Gabriël. 2020. “Identification of Risk Areas and Practices for *Taenia Saginata* Taeniosis/Cysticercosis in Ethiopia: A Systematic Review and Meta-Analysis.” *Parasites & Vectors* 13 (1): 375. [https://doi.org/10.1186/s13071-020-04222-y](https://urldefense.com/v3/__https:/doi.org/10.1186/s13071-020-04222-y__;!!KGKeukY!zorvUpIrPIBnvVyitbDl7VGE4eHKiyEo_s0RtVbCEydz6c-54NL3pAaT0M_qW9eZmK9vHstGDh12xVf8kCvp5jzBEwxneEEL4eHaMg$).

E.G. Pieracci, A.J. Hall, R. Gharpure, A. Haile, E. Walelign, A. Deressa, G. Bahiru, M. Kibebe, H. Walke and E. Belay. One Health 2 (2016) 131–135. <http://dx.doi.org/10.1016/j.onehlt.2016.09.001>

Salomon, Joshua A., Juanita A. Haagsma, Adrian Davis, Charline Maertens de Noordhout, Suzanne Polinder, Arie H. Havelaar, Alessandro Cassini, et al. 2015. “Disability Weights for the Global Burden of Disease 2013 Study.” *The Lancet Global Health* 3 (11): e712–23. [https://doi.org/10.1016/S2214-109X(15)00069-8](https://urldefense.com/v3/__https:/doi.org/10.1016/S2214-109X(15)00069-8__;!!KGKeukY!zorvUpIrPIBnvVyitbDl7VGE4eHKiyEo_s0RtVbCEydz6c-54NL3pAaT0M_qW9eZmK9vHstGDh12xVf8kCvp5jzBEwxneEFs2HQTsQ$).

# **Appendix A: Risk Summaries for FERG Hazards and Metals**

## ***Brucella* spp.**

**Background:**. *Brucella* is transmitted via contact with infected animals or contaminated animal products. Animals that are most commonly infected include sheep, cattle, goats, pigs, and dogs. *Brucella* infection is most commonly associated with consumption of raw/unpasteurized dairy products. Infection from inhalation of the bacteria in slaughter and laboratory settings have also been reported.^1^

| **Annual estimates for risk metrics for this hazard in Ethiopia** | | | | |
| --- | --- | --- | --- | --- |
|  | **Incidence rate**  **(per 100,000)** | **Mortality rate**  **(per 100 000)** | **Case-fatality ratio** | **DALYs rate**  **(per 100,000)** |
| Best estimate | 1 | 0.65 | 0.50 | 0.36 |
| Confidence Interval | (0.013, 63) | (0.000065, 2) | (0.34, 0.66) | (0.004, 19) |

^1^ Centers for Disease Control and Prevention. (2021, July 8). Brucellosis. Centers for Disease Control and Prevention. Retrieved February 15, 2022, from <https://www.cdc.gov/brucellosis/index.html>

## ***Campylobacter* spp.**

**Background:**  *Campylobacter* is most commonly transmitted through contaminated food and water. Person-to-person and animal contact have been associated with illness. Animals can carry *Campylobacter* and contamination can occur during slaughter if sanitation is lacking and/or proper slaughter practices are not followed.^1^

| **Annual estimates for risk metrics for this hazard in Ethiopia** | | | | |
| --- | --- | --- | --- | --- |
|  | **Incidence rate**  **(per 100,000)** | **Mortality rate**  **(per 100 000)** | **Case-fatality ratio** | **DALYs rate**  **(per 100,000)** |
| Best estimate | 2152 | 0.75 | 0.034 | 69 |
| Confidence Interval | (309, 8391) | (0.35, 1.26) | (0.0089, 0.23) | (32, 115) |

^1^ Centers for Disease Control and Prevention. (2021, April 14). Campylobacter (campylobacteriosis). Centers for Disease Control and Prevention. Retrieved February 15, 2022, from <https://www.cdc.gov/campylobacter/index.html>

## ***Cryptosporidium* spp.**

**Background:**. *Cryptosporidium* is a parasite that is transmitted via the fecal-oral route. Contaminated water is the most common source of *Cryptosporidium* infection. Drinking clean water and partaking in recreation water events in clean water is the best way to prevent infection. *Cryptosporidium* has a durable outer layer that allows it to survive in extreme conditions and tolerance to chlorine disinfection.^1^

| **Annual estimates for risk metrics for this hazard in Ethiopia** | | | | |
| --- | --- | --- | --- | --- |
|  | **Incidence rate**  **(per 100,000)** | **Mortality rate**  **(per 100 000)** | **Case-fatality ratio** | **DALYs rate**  **(per 100,000)** |
| Best estimate | 186 | 0.15 | 0.075 | 12 |
| Confidence Interval | (0, 909) | (0, 0.53) | (0.024, 0.28) | (0, 44) |

^1^ Centers for Disease Control and Prevention. (2019, July 19). *Parasites - Cryptosporidium (also known as "Crypto")*. Centers for Disease Control and Prevention. Retrieved February 15, 2022, from <https://www.cdc.gov/parasites/crypto/index.html>

## **Entamoeba histolytica**

**Background:**. *Entamoeba histolytica* is a parasite transmitted via the fecal-oral route. Person-to-person spread is the most common mode of transmission, although consuming food or water that are contaminated with feces are associated with infections. Amebiasis can be extremely difficult to diagnosis due to its long incubation period and inconsistence of presence in fecal samples.^1^

| **Annual estimates for risk metrics for this hazard in Ethiopia** | | | | |
| --- | --- | --- | --- | --- |
|  | **Incidence rate**  **(per 100,000)** | **Mortality rate**  **(per 100 000)** | **Case-fatality ratio** | **DALYs rate**  **(per 100,000)** |
| Best estimate | 742 | 0.05 | 0.0068 | 5 |
| Confidence Interval | (0, 4288) | (0, 0.44) | (0.001, 0.084) | (0, 40) |

^1^ Centers for Disease Control and Prevention. (2021, December 3). *Parasites Amebiasis – Entamoeba histolytica Infection*. Centers for Disease Control and Prevention. Retrieved February 15, 2022, from <https://www.cdc.gov/parasites/amebiasis/general-info.html>

## **Enteropathogenic *E. coli* (EPEC)**

**Background:** Enteropathogenic *E. coli* (EPEC) is transmitted via the fecal-oral route. Consumption of food, water or ice contaminated with EPEC are the most common source of infection. Signs of infection of EPEC include diarrhea, fever, vomiting and in severe cases dehydration. Treatment for EPEC is consumption of fluids and most individuals recover quickly.^1^

| **Annual estimates for risk metrics for this hazard in Ethiopia** | | | | |
| --- | --- | --- | --- | --- |
|  | **Incidence rate**  **(per 100,000)** | **Mortality rate**  **(per 100 000)** | **Case-fatality ratio** | **DALYs rate**  **(per 100,000)** |
| Best estimate | 430 | 1.67 | 0.38 | 136 |
| Confidence Interval | (17, 1331) | (0.073, 3.89) | (0.16, 0.99) | (6, 321) |

^1^ Department of Health Services. (2018, February). *Enteropathogenic E. coli*. State of Wisconsin Bureau of Communicable Diseases. Retrieved February 15, 2022, from <https://www.dhs.wisconsin.gov/publications/p02094.pdf>

## **Enterotoxigenic *E. coli* (ETEC)**

**Background:** Enterotoxigenic *E. coli* (ETEC), also known as traveler’s diarrhea, is transmitted via the fecal-oral route. Consumption of food, water, or ice contaminated with ETEC are the most common source of infection. ETEC symptoms include fever, nausea, vomiting and muscle aches but the illness can be more severe if left untreated. ETEC is typically treated by consuming clear liquids and rehydration salts. In severe cases, antibiotics may be needed.^1^

| **Annual estimates for risk metrics for this hazard in Ethiopia** | | | | |
| --- | --- | --- | --- | --- |
|  | **Incidence rate**  **(per 100,000)** | **Mortality rate**  **(per 100 000)** | **Case-fatality ratio** | **DALYs rate**  **(per 100,000)** |
| Best estimate | 939 | 1.27 | 0.13 | 103 |
| Confidence Interval | (141, 2694) | (0.21, 2.86) | (0.054, 0.38) | (17, 236) |

^1^ Centers for Disease Control and Prevention. (2014, December 1). *Enterotoxigenic E. coli (ETEC)*. Centers for Disease Control and Prevention. Retrieved February 15, 2022, from <https://www.cdc.gov/ecoli/etec>

## ***Giardia* spp.**

**Background:** *Giardia* is a parasite transmitted via the fecal-oral route. Ingestion of contaminated drinking waters or recreational waters is the most common mode of transmission. Contaminated food and person-to-person contact have also been associated with illness. Symptoms can range from gas to diarrhea to dehydration. Practicing good hygiene, including hand washing, and avoiding contaminated water is the best way to prevent infection^1^.

| **Annual estimates for risk metrics for this hazard in Ethiopia** | | | | |
| --- | --- | --- | --- | --- |
|  | **Incidence rate**  **(per 100,000)** | **Mortality rate**  **(per 100 000)** | **Case-fatality ratio** | **DALYs rate**  **(per 100,000)** |
| Best estimate | 663 | 0 | 0 | 0.68 |
| Confidence Interval | (0, 3060) | (0, 0) | (0, 0) | (0, 3) |

^1^ Centers for Disease Control and Prevention. (2021, February 26). *Parasites - Giardia*. Centers for Disease Control and Prevention. Retrieved February 15, 2022, from https://www.cdc.gov/parasites/giardia/general-info.html

## **Hepatitis A**

**Background:** *Hepatitis A* can be spread from person-to-person as well as via the fecal-oral route, particularly when sanitation and personal hygiene are lacking. Symptoms include yellowing of skin or eyes, stomach pain, diarrhea, and joint pain. Most symptoms can be remedied with rest and fluids with severe cases needing medical care^1^.

| **Annual estimates for risk metrics for this hazard in Ethiopia** | | | | |
| --- | --- | --- | --- | --- |
|  | **Incidence rate**  **(per 100,000)** | **Mortality rate**  **(per 100 000)** | **Case-fatality ratio** | **DALYs rate**  **(per 100,000)** |
| Best estimate | 337 | 0.671 | 0.20 | 33 |
| Confidence Interval | (45, 1117) | (0.097, 2.18) | (0.051, 0.79) | (5, 103) |

^1^ Centers for Disease Control and Prevention. (2020, July 28). Viral *Hepatitis - Hepatitis A*. Centers for Disease Control and Prevention. Retrieved February 15, 2022, from <https://www.cdc.gov/hepatitis/hav/afaq.htm>.

^2^ New York State Department of Health. (2007, February). *Hepatitis A* and Food Safety Workers (infectious *hepatis*. Retrieved June 22, 2022, from <https://www.health.ny.gov/diseases/communicable/hepatitis/hepatitis_a/food_service_workers_fact_sheet.htm#:~:text=Hepatitis%20A%20virus%20is%20usually,%22fecal%2Doral%22%20route>.

## ***Listeria monocytogenes***

**Background:** *Listeria monocytogenes (LM)* is transmitted through contaminated foods or from mother to fetus during pregnancy. Listeriosis, the disease caused by *LM*, primarily affects pregnant women, their unborn children, and immunocompromised individuals. Infections can range in severity from mild illness to sepsis to meningitis to death. *LM* can grow at refrigerated temperatures, so the best protection is avoiding high risk foods (e.g., deli meats, raw milk) and keeping food storage areas clean^1^.

| **Annual estimates for risk metrics for this hazard in Ethiopia** | | | | |
| --- | --- | --- | --- | --- |
|  | **Incidence rate**  **(per 100,000)** | **Mortality rate**  **(per 100 000)** | **Case-fatality ratio** | **DALYs rate**  **(per 100,000)** |
| Best estimate | 0.14 | 0.03 | 22.39 | 1 |
| Confidence Interval | (0.000025, 2.5) | (0.0000056, 0.055) | (19.24, 25.77) | (0.0002, 21) |

^1^ Food and Drug Administration. (2018, March 22). *What You Need to Know About Preventing Listeria Infections.* Food and Drug Administration. Retrieved February 15, 2022, from <https://www.fda.gov/food/buy-store-serve-safe-food/what-you-need-know-about-preventing-listeria-infections.>

## **Non-typhoidal *S.* enterica**

**Background:**  *Non-typhoidal Salmonella* is transmitted via the fecal-oral route. The most common mode of transmission is though person-to-person. Outbreaks have also been associated with infected animals and their environment. Contamination of food with fecal material can occur anywhere along the farm-to-fork continuum. Practicing good hygiene and sanitation practices limits the spread of the bacteria^1^.

| **Annual estimates for risk metrics for this hazard in Ethiopia** | | | | |
| --- | --- | --- | --- | --- |
|  | **Incidence rate**  **(per 100,000)** | **Mortality rate**  **(per 100 000)** | **Case-fatality ratio** | **DALYs rate**  **(per 100,000)** |
| Best estimate | 875 | 1.55 | 0.17 | 116 |
| Confidence Interval | (100, 3159) | (0.34, 2.65) | (0.053 , 0.77) | (25, 201) |

^1^World Health Organization. (2018, February 20). *Salmonella (non-typhoidal).* World Health Organization. Retrieved February 15, 2022, from https://www.who.int/news-room/fact-sheets/detail/salmonella-(non-typhoidal)#:~:text=Salmonellosis%20in%20humans%20is%20generally,been%20implicated%20in%20its%20transmission.

## **Norovirus**

**Background:** Norovirus is a highly contagious virus most commonly transmitted person-to-person. Infections also occur through the fecal-oral route. Contamination of food with fecal material can occur anywhere along the farm-to-fork continuum, including at the source^1^.

| **Annual estimates for risk metrics for this hazard in Ethiopia** | | | | |
| --- | --- | --- | --- | --- |
|  | **Incidence rate**  **(per 100,000)** | **Mortality rate**  **(per 100 000)** | **Case-fatality ratio** | **DALYs rate**  **(per 100,000)** |
| Best estimate | 1609 | 1 | 0.063 | 76 |
| Confidence Interval | (0, 5882) | (0, 3) | (0.026, 0.14) | (0, 226) |

^1^ Centers for Disease Control and Prevention. (2021, March 5). *Norovirus*. Centers for Disease Control and Prevention. Retrieved February 15, 2022, from <https://www.cdc.gov/norovirus/index.html>.

## **Shiga toxin-producing *E. coli***

**Background:** *Shiga toxin-producing E. coli* (STEC) is transmitted via food and water that have been contaminated with fecal matter. Animals are the reservoir for STEC and contamination of the carcass can occur during slaughter and processing. Fruits and vegetables can also become contaminate through environmental exposures such as contaminated irrigation water or animal intrusion. STEC can cause hemolytic uremic syndrome, a serious illness that results in organ failure^1^.

| **Annual estimates for risk metrics for this hazard in Ethiopia** | | | | |
| --- | --- | --- | --- | --- |
|  | **Incidence rate**  **(per 100,000)** | **Mortality rate**  **(per 100 000)** | **Case-fatality ratio** | **DALYs rate**  **(per 100,000)** |
| Best estimate | 0.5 | 0.000057 | 0.011 | 0.0048 |
| Confidence Interval | (0.07, 2) | (0.0000052, 0.00024) | (0.0045 , 0.024 ) | (0.0006, 0.02) |

^1^ Centers for Disease Control and Prevention. (2014, December 1). *E. coli (Escherichia coli) (ETEC).* Centers for Disease Control and Prevention. Retrieved February 15, 2022, from <https://www.cdc.gov/ecoli/general/index.html>.

## ***Shigella* spp.**

**Background**: *Shigella* is a bacterium that is commonly transmitted via person-to-person. Contaminated food and water have also been associated with shigellosis; the illness caused by *Shigella.* Proper sanitation and handwashing are critical in preventing contamination and illness^1^.

| **Annual estimates for risk metrics for this hazard in Ethiopia** | | | | |
| --- | --- | --- | --- | --- |
|  | **Incidence rate**  **(per 100,000)** | **Mortality rate**  **(per 100 000)** | **Case-fatality ratio** | **DALYs rate**  **(per 100,000)** |
| Best estimate | 435 | 0.45 | 0.093 | 37 |
| Confidence Interval | (0, 3296) | (0, 1.79) | (0.024, 0.72 ) | (0, 147) |

^1^ Centers for Disease Control and Prevention. (2020, October 8). *Shigella – Shigellosis*. Centers for Disease Control and Prevention. Retrieved February 15, 2022, from <https://www.cdc.gov/shigella/general-information.html>.

## ***Vibrio* spp.**

**Background:**  *Vibrio cholerae* bacteria is most commonly transmitted to humans through food and water contaminated by sewage. The disease can also be acquired by consuming raw or undercooked mollusks or shellfish harvested from unsafe water sources. The best prevention of the disease is to purchase or consume food and water from safe sources, and practice good handwashing, especially after using the bathroom. Casual contact with an infected individual is not a risk for contracting infection^1^.

| **Annual estimates for risk metrics for this hazard in Ethiopia** | | | | |
| --- | --- | --- | --- | --- |
|  | **Incidence rate**  **(per 100,000)** | **Mortality rate**  **(per 100 000)** | **Case-fatality ratio** | **DALYs rate**  **(per 100,000)** |
| Best estimate | 72 | 2.72 | 3.80 | 190 |
| Confidence Interval | (2, 207) | (0.075, 7.30) | (2.61 , 5.01 ) | (5, 511) |

^1^ Centers for Disease Control and Prevention. (2021, June 30). *Cholera*. Centers for Disease Control and Prevention. Retrieved February 15, 2022, from <https://www.cdc.gov/cholera/general/index.html#one>.

## **Aflatoxin B1**

**Background:** Aflatoxins are a natural toxin found in soils, and foods. The toxigenic strains of aflatoxins, which are derived from the Aspergillus flavus fungus, are harmful to humans and are known to cause liver failure and cancer. Foods known to be contaminated by Aflatoxins are grains e.g. maize, nuts, dairy products, tea, spices, and cocoa. Improper storage and drying practices is a major risk factor for the formation of the toxin^1^.

| **Annual estimates for risk metrics for this hazard in Ethiopia** | | | | |
| --- | --- | --- | --- | --- |
|  | **Incidence rate**  **(per 100,000)** | **Mortality rate**  **(per 100 000)** | **Case-fatality ratio** | **DALYs rate**  **(per 100,000)** |
| Best estimate | 0.019 | 0.013 | 90.19 | 0.46 |
| Confidence Interval | (0.00019 – 0.094) | (0.00017 – 0.085) | (90.19, 90.19) | (0.62– 3) |

^1^Negash, Demissie. “A Review of Aflatoxin: Occurrence, Prevention, and Gaps in Both Food and Feed Safety.” Journal of Nutritional Health & Food Engineering Volume 8, no. Issue 2 (March 29, 2018). https://doi.org/10.15406/jnhfe.2018.08.00268

^2^Gizachew, Dawit, Barbara Szonyi, Azage Tegegne, Jean Hanson, and Delia Grace. “Aflatoxin Contamination of Milk and Dairy Feeds in the Greater Addis Ababa Milk Shed, Ethiopia.” *Food Control* 59 (January 1, 2016): 773–79. https://doi.org/10.1016/j.foodcont.2015.06.060.

## ***Ascaris* spp**

**Background:**  *Ascaris lumbricoides*, or *A. lumbricoides* is the world's most common human nematode and is one of three soil-transmitted parasites of public health and socio-economic concern. The World Health Organization lists *A. lumbricoides* a neglected tropical diseases (NTD), infecting more than one billion people. When the eggs of the parasite are ingested, they reside in the intestine of the human host. Most often people appear asymptomatic, but symptoms can include abdominal discomfort and pain. An infestation in children can inhibit normal growth. The eggs are ingested by eating contaminated foods or putting soiled hands in the mouth. Good hygiene practices and washing fruits and vegetables before consuming are effective preventative measures for preventing an intestinal infestation.

| **Annual estimates for risk metrics for this hazard in Ethiopia** | | | | |
| --- | --- | --- | --- | --- |
|  | **Incidence rate**  **(per 100,000)** | **Mortality rate**  **(per 100 000)** | **Case-fatality ratio** | **DALYs rate**  **(per 100,000)** |
| Best estimate | 92 | 0.0093 | 0.011 | 6 |
| Confidence Interval | 1. – 164) | (0.000013– 0.01) | (0.000037, 0.10) | (1– 14) |

^1^Cavallero, Serena, Viliam Snabel, Francesca Pacella, Vitantonio Perrone, and Stefano D’Amelio. 2013. “Phylogeographical Studies of Ascaris Spp. Based on Ribosomal and Mitochondrial DNA Sequences.” Edited by Xiao-Nong Zhou. *PLoS Neglected Tropical Diseases* 7 (4): e2170. https://doi.org/10.1371/journal.pntd.0002170.

^2^Monteiro, Kerla J. L., Deiviane A. Calegar, Jessica P. Santos, Polyanna A. A. Bacelar, Beatriz Coronato-Nunes, Elis Regina C. Reis, Márcio N. Boia, Filipe A. Carvalho-Costa, and Lauren H. Jaeger. 2019. “Genetic Diversity of Ascaris Spp. Infecting Humans and Pigs in Distinct Brazilian Regions, as Revealed by Mitochondrial DNA.” Edited by Tzen-Yuh Chiang. *PLOS ONE* 14 (6): e0218867. https://doi.org/10.1371/journal.pone.0218867.

## **Dioxins**

**Background:**  *Dioxins* are unintentional contaminants released into the environment from combustion processes. *Dioxins* and related compounds, including dioxin-like polychlorinated biphenyls (referred to collectively as DLCs), accumulate in the lipid component of animal foods. DLC exposure through foods occurs primarily by consumption of animal fats. Public concern persists about the safety of the food supply and potential adverse outcomes to DLC exposure in sensitive and highly exposed population groups, especially developing fetuses and infants^1^.

| **Annual estimates for risk metrics for this hazard in Ethiopia** | | | | |
| --- | --- | --- | --- | --- |
|  | **Incidence rate**  **(per 100,000)** | **Mortality rate**  **(per 100 000)** | **Case-fatality ratio** | **DALYs rate**  **(per 100,000)** |
| Best estimate | 0.092 | 0 | 0 | 0.11 |
| Confidence Interval | (0.003 – 9.30) | (0 – 0) | (0 – 0) | (0.0033– 11) |

^1^National Academy of Sciences (U.S.), and Institute of Medicine (U.S.), eds. 2003. *Dioxins and Dioxin-like Compounds in the Food Supply: Strategies to Decrease Exposure*. Washington, D.C: National Academies Press

## ***Echinococcus granulosus***

**Background:**  *Echinococcus granulosus* (sensu lato), also known as tapeworm, is a cestode helminth belonging to the Taeniidae family. During infection, fluid-filled larval cysts develop and localize in the liver and lungs and, to a lesser extent, in the abdominal cavity, muscle, heart, bone and nervous system. Human hosts can remain asymptomatic for years, but when a cyst bursts the individual may experience fever, hives, increased white blood cell count, and possibly anaphylactic shock. Humans become infected by consuming embryonic eggs excreted in the feces of an infected host^1^.

| **Annual estimates for risk metrics for this hazard in Ethiopia** | | | | |
| --- | --- | --- | --- | --- |
|  | **Incidence rate**  **(per 100,000)** | **Mortality rate**  **(per 100 000)** | **Case-fatality ratio** | **DALYs rate**  **(per 100,000)** |
| Best estimate | 2 | 0.017 | 1.09 | 1.43 |
| Confidence Interval | (0.5 – 3) | (0.0036 – 0.054) | (0.40, 2.51) | (0.36– 4) |

^1^Khademvatan, S., H. Majidiani, M. Foroutan, K. Hazrati Tappeh, S. Aryamand, and H.R. Khalkhali. 2019. “*Echinococcus Granulosus* Genotypes in Iran: A Systematic Review.” *Journal of Helminthology* 93 (2): 131–38. https://doi.org/10.1017/S0022149X18000275.

^2^Budke, Christine M., Qiu Jiamin, Philip S. Craig, and Paul R. Torgerson. 2005. “Modeling the Transmission of Echinococcus Granulosus and Echinococcus Multilocularis in Dogs for a High Endemic Region of the Tibetan Plateau.” *International Journal for Parasitology* 35 (2): 163–70. https://doi.org/10.1016/j.ijpara.2004.10.026.

## ***Fasciola* spp.**

**Background:** *Fasciola hepatica* and *Fasciola gigantica,* also known as liver fluke, are parasites that infect both human and animals. These parasites are found all over the world, especially in areas where there are sheep and cattle. Humans become infected by consuming raw water plants contaminated with larvae. In the acute phase, symptoms include nausea, vomiting, and abdominal pain. In the chronic phase, the individual may experience a blockage of the bile duct. The disease is often treatable and preventable^1^.

| **Annual estimates for risk metrics for this hazard in Ethiopia** | | | | |
| --- | --- | --- | --- | --- |
|  | **Incidence rate**  **(per 100,000)** | **Mortality rate**  **(per 100 000)** | **Case-fatality ratio** | **DALYs rate**  **(per 100,000)** |
| Best estimate | 0.0052 | 0 | 0 | 0.038 |
| Confidence Interval | (0.0018 – 0.015) | (0 – 0) | (0 – 0) | (0.013– 0.1) |

^1^Shafiei, Reza, Bahador Sarkari, Seyed Mahmuod Sadjjadi, Gholam Reza Mowlavi, and Abdolali Moshfe. 2014. “Molecular and Morphological Characterization of Fasciola Spp. Isolated from Different Host Species in a Newly Emerging Focus of Human Fascioliasis in Iran.” *Veterinary Medicine International* 2014: 1–10. https://doi.org/10.1155/2014/405740.

^2^Keiser, Jennifer, Dirk Engels, Gottfried Büscher, and Jürg Utzinger. 2005. “Triclabendazole for the Treatment of Fascioliasis and Paragonimiasis.” *Expert Opinion on Investigational Drugs* 14 (12): 1513–26. https://doi.org/10.1517/13543784.14.12.1513.

## ***Mycobacterium bovis***

**Background:** *Mycobacterium bovis* is a member of the Mycobacterium tuberculosis complex (MTBC). *Mycobacterium bovis* is commonly found in cattle, bison, and elk. People may contract the bacteria by eating or drinking unpasteurized dairy products and/or by direct contact with a diseased animal through an open wound. If contracted, the disease may negatively affect the lungs and lymph nodes, although some people are asymptomatic. Person-to-person transmission can occur through coughs and sneezes.

| **Annual estimates for risk metrics for this hazard in Ethiopia** | | | | |
| --- | --- | --- | --- | --- |
|  | **Incidence rate**  **(per 100,000)** | **Mortality rate**  **(per 100 000)** | **Case-fatality ratio** | **DALYs rate**  **(per 100,000)** |
| Best estimate | 7 | 0.37 | 5.48 | 22 |
| Confidence Interval | (4 - 10) | (0.22-0.54) | (3.63, 8.21) | (13-32) |

^1^Duffy, Shannon C, Sreenidhi Srinivasan, Megan A Schilling, Tod Stuber, Sarah N Danchuk, Joy S Michael, Manigandan Venkatesan, et al. 2020. “Reconsidering Mycobacterium Bovis as a Proxy for Zoonotic Tuberculosis: A Molecular Epidemiological Surveillance Study.” *The Lancet Microbe* 1 (2): e66–73. https://doi.org/10.1016/S2666-5247(20)30038-0.

^2^Butler, Rachel E, Alex A. Smith, Tom A. Mendum, Aneesh Chandran, Huihai Wu, Louise Lefrançois, Mark Chambers, Thierry Soldati, and Graham R Stewart. 2020. “Mycobacterium Bovis Uses the ESX-1 Type VII Secretion System to Escape Predation by the Soil-Dwelling Amoeba Dictyostelium Discoideum.” *The ISME Journal* 14 (4): 919–30. https://doi.org/10.1038/s41396-019-0572-z.

## ***Salmonella* Paratyphi A**

**Background:** *Salmonella enterica* serovar *Paratyphi A* is an emerging pathogen and an understudied cause of enteric fever that has started replacing *Salmonella Typhi (S. Typhi)* in some endemic regions. *S. Paratyphi A* is also commonly reported among travelers returning from endemic regions and remains one of the leading causes of enteric (typhoid) fever. *S. Paratyphi A* can cause significant health problems, particularly in low– and middle-income countries such as Ethiopia.

| **Annual estimates for risk metrics for this hazard in Ethiopia** | | | | |
| --- | --- | --- | --- | --- |
|  | **Incidence rate**  **(per 100,000)** | **Mortality rate**  **(per 100 000)** | **Case-fatality ratio** | **DALYs rate**  **(per 100,000)** |
| Best estimate | 24 | 0.157 | 0.65 | 11 |
| Confidence Interval | (0 – 87) | (0 – 0.56) | (0.65, 0.65) | (0– 40) |

^1^Rahman, Sadia Isfat Ara, To Nguyen Thi Nguyen, Farhana Khanam, Nicholas R. Thomson, Zoe A. Dyson, Alyce Taylor-Brown, Emran Kabir Chowdhury, Gordon Dougan, Stephen Baker, and Firdausi Qadri. 2021. “Genetic Diversity of Salmonella Paratyphi A Isolated from Enteric Fever Patients in Bangladesh from 2008 to 2018.” Edited by Abiola Senok. *PLOS Neglected Tropical Diseases* 15 (10): e0009748. https://doi.org/10.1371/journal.pntd.0009748.

^2^Amsalu, Tadele, Chalachew Genet, and Yesuf Adem Siraj. 2021. “Salmonella Typhi and Salmonella Paratyphi Prevalence, Antimicrobial Susceptibility Profile and Factors Associated with Enteric Fever Infection in Bahir Dar, Ethiopia.” *Scientific Reports* 11 (1): 7359. https://doi.org/10.1038/s41598-021-86743-9.

## ***Salmonella* Typhi**

**Background:** *Salmonella spp.* are gram-negative flagellated bacteria that can cause food- and waterborne [gastroenteritis](https://www.sciencedirect.com/topics/immunology-and-microbiology/gastroenteritis) and typhoid fever in humans. *S. Typhi* causes an estimated 11–21 million new cases of typhoid fever worldwide annually. Transmission occurs through the fecal-oral route, typically by ingesting contaminated food or water. Symptoms include headache, abdominal pain, and high fever. *S. Typhi* transiently occupies the gastrointestinal niche, causing minimal inflammation, before rapidly disseminating to systemic sites where it can persist chronically.

| **Annual estimates for risk metrics for this hazard in Ethiopia** | | | | |
| --- | --- | --- | --- | --- |
|  | **Incidence rate**  **(per 100,000)** | **Mortality rate**  **(per 100 000)** | **Case-fatality ratio** | **DALYs rate**  **(per 100,000)** |
| Best estimate | 106 | 0.68 | 0.65 | 49 |
| Confidence Interval | (0 – 378) | (0 – 2.44) | (0.65, 0.65) | (0– 174) |

^1^Mathur, Ramkumar, Hyunju Oh, Dekai Zhang, Sung-Gyoo Park, Jin Seo, Alicia Koblansky, Matthew S. Hayden, and Sankar Ghosh. 2012. “A Mouse Model of Salmonella Typhi Infection.” *Cell* 151 (3): 590–602. https://doi.org/10.1016/j.cell.2012.08.042.

^2^Brewer, Susan M., Christian Twittenhoff, Jens Kortmann, Sky W. Brubaker, Jared Honeycutt, Liliana Moura Massis, Trung H. M. Pham, Franz Narberhaus, and Denise M. Monack. 2021. “A Salmonella Typhi RNA Thermosensor Regulates Virulence Factors and Innate Immune Evasion in Response to Host Temperature.” Edited by Leigh Knodler. *PLOS Pathogens* 17 (3): e1009345. https://doi.org/10.1371/journal.ppat.1009345.

## ***Toxoplasma gondii***

**Background:** *Toxoplasma gondii* is a protozoan parasite that is the causative agent of toxoplasmosis, an infection with high prevalence worldwide and significant medical and veterinary importance. *T. gondii* is transmitted by several pathways but typically involve consumption of oocysts released in the feces of the felines into the environment or undercooked meat.

| **Annual estimates for risk metrics for this hazard in Ethiopia** | | | | |
| --- | --- | --- | --- | --- |
|  | **Incidence rate**  **(per 100,000)** | **Mortality rate**  **(per 100 000)** | **Case-fatality ratio** | **DALYs rate**  **(per 100,000)** |
| Best estimate | 353 | 0.023 | 0.0065 | 28 |
| Confidence Interval | (163 – 617) | (0.0082 – 0.05) | (0.0033, 0.012 ) | (13– 51) |

^1^Attias, Márcia, Dirceu E. Teixeira, Marlene Benchimol, Rossiane C. Vommaro, Paulo Henrique Crepaldi, and Wanderley De Souza. 2020. “The Life-Cycle of Toxoplasma Gondii Reviewed Using Animations.” *Parasites & Vectors* 13 (1): 588. https://doi.org/10.1186/s13071-020-04445-z.

^2^Angel, Sergio O., Laura Vanagas, Diego M. Ruiz, Constanza Cristaldi, Ana M. Saldarriaga Cartagena, and William J. Sullivan. 2020. “Emerging Therapeutic Targets Against Toxoplasma Gondii: Update on DNA Repair Response Inhibitors and Genotoxic Drugs.” *Frontiers in Cellular and Infection Microbiology* 10 (June): 289. https://doi.org/10.3389/fcimb.2020.00289.

## ***Trichinella* spp.**

**Background:** *Trichinella* nematodes are one of the most significant foodborne parasites, infecting a number of domestic and wild vertebrates, including humans. All species and genotypes of *Trichinella* are zoonotic and transmitted by consuming raw or insufficiently cooked meat. Symptoms are diverse and may include generalized fever, abdominal pain, diarrhea, nausea, vomiting, or myalgias.

| **Annual estimates for risk metrics for this hazard in Ethiopia** | | | | |
| --- | --- | --- | --- | --- |
|  | **Incidence rate**  **(per 100,000)** | **Mortality rate**  **(per 100 000)** | **Case-fatality ratio** | **DALYs rate**  **(per 100,000)** |
| Best estimate | 0.00066 | 0.000024 | 3.57 | 0.0014 |
| Confidence Interval | (0.00022 – 0.001) | (0.0000078 – 0.00004) | (3.57 , 3.57 ) | (0.00047 – 0.0024) |

^1^Sharma, Rajnish, N. Jane Harms, Piia M. Kukka, Thomas S. Jung, Sarah E. Parker, Sasha Ross, Peter Thompson, Benjamin Rosenthal, Eric P. Hoberg, and Emily J. Jenkins. 2021. “High Prevalence, Intensity, and Genetic Diversity of Trichinella Spp. in Wolverine (Gulo Gulo) from Yukon, Canada.” *Parasites & Vectors* 14 (1): 146. https://doi.org/10.1186/s13071-021-04636-2.

^2^Rawla P, Sharma S. Trichinella Spiralis. [Updated 2021 Dec 12]. In: StatPearls [Internet]. Treasure Island (FL): StatPearls Publishing; 2022 Jan-. Available from: https://www.ncbi.nlm.nih.gov/books/NBK538511/?report=classic

# **Appendix B: Risk Summary Sheets for Non-FERG Hazards**

## **Acrylamide**

**Background:** Acrylamide (AA), according to EFSA,^1,2^ is a chemical formed as a by-product of the Maillard reaction in certain foods, especially in those foods containing asparagine and reducing sugars that are prepared at temperatures above 120 °C and at low moisture levels. Fried potato products, coffee, biscuits, crackers, crisp bread and soft bread may contribute most to AA exposure of consumers. Carcinogenicity, genotoxicity, neurotoxicity, and reproductive toxicity were reported as adverse health effects of human exposure to AA.

| **Rationale for Inclusion** | Since the recognition of Acrylamide in 2002,^1,2^ several researchers have studied its formation and occurrence in heat processed food. There are very limited studies on the level of AA in foods consumed in different parts of Africa. The potential of dietary AA exposure is of concern given that most staple diets consumed in Africa are made of carbohydrate-based foods subjected to varying degrees of heat during processing.^4^ |
| --- | --- |
| **Overview of Available Data** | The level of dietary AA exposure in Ethiopia is not known. One study found significant variations in AA content in Keribo, a fermented traditional beverage consumed in rural and urban Ethiopia (e.g. 3440 μg AA/kg and 1320 μg AA/kg in Keribo prepared from deep roasted and light roasted unmalted barley, respectively).^5^ A study focused on Addis Ababa found mean AA levels in coffee powder, potato chips and French fries to be 421 μg/kg, 1298 μg/kg and 615 μg/kg, respectively.^6^ It is unknown whether and what food types consumed in Ethiopia’s urban and rural communities cause significant dietary AA exposure. |
| **Proportion foodborne** | AA is heat generated in food. However, human exposure to AA can be through other routes (e.g., inhalation; skin absorption) since it is an industrial chemical with multiple applications.^7^ |
| **Identified Health Outcomes & Disability Weights** | Studies on human subjects have provided limited and inconsistent evidence of increased risk of developing cancer. Based on studies in laboratory animals, EFSA considers that AA in food potentially increases the risk of developing cancer for consumers in all age groups.^1^ In a diet related AA study in Denmark^3^, the rate of dietary AA exposure associated ovarian, uterine (endometrial), breast and kidney cancer (though to be somewhat more specifically related to consequences of AA exposure) was estimated next to the rate associated to total cancer. |
| **Incidence** | Frequency of exposure to AA through food in Ethiopia is not known, though typical foods with potential high AA levels (e.g., coffee, fried potato products) are consumed in Ethiopia, especially in urban settings. Using 2010 data for Ethiopia^8^ and total cancer related AA rates for Denmark in Jakobsen et al. (2016)^3^ as a benchmark, the incidence of AA associated cancer in Ethiopia is estimated as 2.38 (0.11-23.52) in the country or 0.0027 (0.00012-0.027) per 100.000 inhabitants. |
| **Percentage of cases by outcome** | Not available |
| **Disease Duration** | Not available, especially because the health effects of (dietary) AA exposure may not be observed for years following exposure. |
| **Case fatality rate or ratio** | No specific data available from Ethiopia, but based on the benchmarking approach used to derive the incidence of AA associated dietary exposure the CFR was derived to be 0.631 (0.196-0.956) |
| **Average age of fatal cases** | No data available. However, children may be more vulnerable due to their smaller body mass as compared to adults.^1,3^ |
| **DALYs** | Jakobsen et al. (2016)^3^ used different modelling approaches to assess that, for Denmark, the annual burden of disease of AA may be 0.027 to 0.14 DALY/100k based on the four AA associated cancer types and 0.19 to 1.8 DALY/100k based on total cancer. Using this study for benchmarking based on total cancer incidence, the annual burden of disease in Ethiopia is about 0.063 (0.0027-0.63) DALY/100k. Note that studies in the Netherlands^9^ and China^10^ estimated burden of disease values of up to 2–4 DALY/100k.y and 1.7 DALY/100k.y, respectively |
| **References**  ^1^European Food Safety Authority. “Acrylamide in Food Is a Public Health Concern,” June 4, 2015. <https://www.efsa.europa.eu/en/press/news/150604>.  ^2^ European Food Safety Authority. “Acrylamide”. <https://www.efsa.europa.eu/en/topics/topic/acrylamide>  ^3^Jakobsen, Lea Sletting, Kit Granby, Vibeke Kildegaard Knudsen, Maarten Nauta, Sara Monteiro Pires, and Morten Poulsen. “Burden of Disease of Dietary Exposure to Acrylamide in Denmark.” *Food and Chemical Toxicology* 90 (April 1, 2016): 151–59. <https://doi.org/10.1016/j.fct.2016.01.021>.  ^4^Adebo, Oluwafemi Ayodeji, Eugenie Kayitesi, Janet AdeyinkaAdebiyi, Sefater Gbashi, Makumba Chewe Temba, Adeseye Lasekan, Judith Zanele Phoku, and Patrick Berka Njobeh. “Mitigation of Acrylamide in Foods: An African Perspective.” *Acrylic Polymers in Healthcare*, November 2, 2017. <https://doi.org/10.5772/intechopen.68982>.  ^5^Dibaba, Kumela, Lelise Tilahun, Neela Satheesh, and Melkayo Geremu. “Acrylamide Occurrence in Keribo: Ethiopian Traditional Fermented Beverage.” *Food Control* 86 (April 1, 2018): 77–82. <https://doi.org/10.1016/j.foodcont.2017.11.016>.  ^6^Henok, Deribew , Ashagrie, Woldegiorgis. “Acrylamide levels in coffee powder, potato chips and French fries in Addis Ababa city in Ethiopia.” *Food Control* 123 (May, 2021): 77–82. <https://doi.org/10.1016/j.foodcont.2020.107727>.  ^7^ Pelucchi, C., C. La Vecchia, C. Bosetti, P. Boyle, and P. Boffetta. Exposure to acrylamide and human cancer-a review and meta-analysis of epidemiologic studies. Annals of Oncology 22, 2011, 1487-1499. <https://doi.org/10.1093/annonc/mdq610>  ^8^ Global Burden of Disease Study 2019 (GBD 2019). <https://ghdx.healthdata.org/gbd-2019>; query tool <https://vizhub.healthdata.org/gbd-results/>  ^9^ Van Kreijl, C.F., Knaap, A., Van Raaij, J.M.A., 2006. Our food, our health - Healthy diet and safe food in the Netherlands. RIVM Rep. 270555009. <https://www.efsa.europa.eu/sites/default/files/event/2006/af060519-ax10.pdf>  ^10^ Yiling Li, Jialin Liu, Yibaina Wang and Sheng Wei, 2022. Cancer risk and disease burden of dietary acrylamide exposure in China, 2016. <https://doi.org/10.1016/j.ecoenv.2022.113551> | |

## **Aflatoxin M1**

Aflatoxin M1 (AFM1) is one metabolite of AFB1. AFM1, a hydroxylated metabolite of AFB1, is secreted in milk from mammalian species or excreted in urine. Hence, AFM1 may be present in milk and other dairy products sourced from animals that consumed AFB1.

| **Rationale for Inclusion** | Based on both average daily liquid milk consumption patterns and levels of AFM1 contamination in milk, a recent study revealed Ethiopia, Mexico, Sudan, Syria, and Pakistan have higher exposure to AFM1. The Average Daily Dose of AFM1 (ng/kg bw/day) in Ethiopia is 0.79. Ethiopia has set an AFM1 standard of 0.05 µg/kg, adopted from the EU^1^. |
| --- | --- |
| **Overview of Available Data** | There is a wealth of studies measuring AFM1 levels in liquid milk in various forms, showing dramatically different results for AFM1 occurrence across the world as well as within the same country. Sub- Saharan Africa and South Asia have been identified to have AFM1 levels occasionally (and sometimes dramatically) exceeding the FDA action levels. Liquid milk samples from Ethiopia, Kenya, Nigeria, South Africa, Tanzania, and Sudan have been shown to exceed the US FDA action level of 0. 5 μg/L AFM1^2^. Globally, exposure to AFM1 is approximately 100-fold lower than exposure to AFB1. |
| **Proportion foodborne** | Exposure to AFM1, mainly occurs through consumption of contaminated milk. Additional exposure to AFM1 can be present due to consumption of other dairy products such as cheese, butter, and yogurt^2^. |
| **Identified Health Outcomes & Disability Weights** | AFM1 is a metabolite of AFB1 which is secreted in the milk of mammals^2^. An *in vitro* study in human cell lines demonstrated AFM1 alone may have direct toxic potential. Following high-dose oral administration in rats, AFM1 could be carcinogenic; but much less so than AFB1. The Joint Expert Committee on Food Additives (JECFA) of the Food and Agriculture Organization of the United Nations and the World Health Organization estimated AFM1 was ten times less carcinogenic than AFB1. IARC classifies AFM1 as a Group 2B possible carcinogen, based on suggestive evidence in animal studies but no evidence from human studies^1^. |
| **Incidence** | A recent study to estimate the global risk of AFM1-related liver cancer through liquid milk consumption found that AFM1 may contribute about 0.001% of total annual HCC (liver cancer) cases globally assuming there is no synergy between AFM1 and HBV. If there is synergy between AFM1 and HBV infection, AFM1 may contribute about 0.003% of all HCC cases worldwide. In each case, the total expected AFM1-attributable cancer cases are about 13-32 worldwide. For Ethiopia, findings showed that the annual HCC cases, assuming synergy between AFM1 and chronic HBV infection (cases/year/100,000 population/ng AFM1 consumed) is 3.62 , while the annual HCC cases assuming no HBV synergy (cases/year/100,000 population/ng AFM1 consumed) is 0.875. Therefore, the total expected AFM1-attributable cancer cases range from about 0.875 to 3.62^1^. |
| **Percentage of cases by outcome** | If AFM1 is genotoxic, it causes the same disease (HCC) as AFB1 so disease burden estimation can be based on FERG estimates for AFB1. |
| **Duration** | Not relevant, as the contribution of YLD to the total burden of HCC by AFB1 is <1%. |
| **Case fatality rate** | AFRE medians: 433 cases, 376 deaths |
| **Average age of fatal cases** | Not needed |
| **DALYs** | AFRE: YLD per case 0.2 (0.1-0.3), YLL per case 33 (31-35), DALY per case 33 (31-35) |
| **References**  ^1^Saha Turna, Nikita, Arie Havelaar, Adegbola Adesogan and Felicia Wu. 2022. “Aflatoxin M1 in Milk Does Not Contribute Substantially to Global Liver Cancer Incidence.” *The American Journal of Clinical Nutrition*.  ^2^Saha Turna, Nikita, and Felicia Wu. 2021. “Aflatoxin M1 in Milk: A Global Occurrence, Intake, & Exposure Assessment.” *Trends in Food Science & Technology* 110 (April): 183–92. <https://doi.org/10.1016/j.tifs.2021.01.093>. | |

## ***Bacillus anthracis***

*Bacillus anthracis* is a Gram-positive bacterium that produces extremely hardy spores that can survive in the environment for decades and is naturally found in soils. Livestock and other animals, particularly ruminants, around the world can be infected. People get infected with anthrax when spores get into the body and are activated in the body, producing toxins (poisons) that cause severe illness.

| **Rationale for Inclusion** | Anthrax was ranked the #3 priority zoonotic disease in Ethiopia in a risk ranking exercise conducted by the US CDC, EPHI and various other organizations^1^. Skin and intestinal anthrax are reported in various regions where many outbreaks in livestock (and wildlife) continue to occur. Human consumption of animal products or tissues harvested from an infected animal can lead to gastrointestinal anthrax. |
| --- | --- |
| **Overview of Available Data** | Bahiru et al. (2016) retrospectively analyzed the data from EPHI and MOA collected on human and animal anthrax in Ethiopia. Conclusions from this analysis are for all anthrax infections. No estimates are available for the proportion of different forms of anthrax (cutaneous, inhalation, gastrointestinal) ^2^. |
| **Proportion foodborne** | Gastrointestinal anthrax is 100% foodborne. |
| **Identified Health Outcomes & Disability Weights** | Notes from Workshop: Causes severe morbidity and mortality Common problem, especially in rural community, Raw meat consumption is high, Prevalent in animals.  Gastrointestinal anthrax presents with typical food poisoning symptoms in humans but can progress to vomiting blood, severe diarrhea, and severe abdominal pain. Prompt diagnosis and treatment with antibiotics can reduce morbidity and mortality^3^.  Mild disease: diarrhea, nausea, headache: 0.061/0.074  Severe disease: vomiting blood, severe diarrhea, abdominal pain: 0.247 |
| **Incidence** | Bahiru et al. reported an average prevalence/ 100,000 population/year of 1.3, ranging from 0.0 to 6.7. “A total of 5197 and 26737 cases and 86 and 8523 deaths of human and animal anthrax respectively were documented the last five years (2009- 2013) nationally.”  Because anthrax is more common in the developing world where reporting is not common, the true incidence of GI anthrax is unknown. (Maddah et al., 2013) |
| **Percentage of cases by outcome** | According to the US CDC “Without treatment, more than half of patients with gastrointestinal anthrax die. However, with proper treatment, 60% of patients survive.” |
| **Disease Duration** | Seems relatively short as penicillin is required to treat and sometimes a ventilator. (see Maddah et al. 2013) |
| **Case fatality ratio** | Bahiru et al. reported an average case fatality rate of 1.7, ranging from 0.0 to 6.1%^2^.  Mortality rate has been estimated at 4 to 50%^4^. |
| **Average age of fatal cases** | No data available, see note on incidence. |
| **DALYs** | No global or country DALY estimates were identified. |
| **References**  Pieracci EG, Hall AJ, Gharpure R, Haile A, Walelign E, Deressa A, Bahiru G, Kibebe M, Walke H, Belay E. Prioritizing zoonotic diseases in Ethiopia using a one health approach. One Health. 2016 Dec;2:131-135. doi: 10.1016/j.onehlt.2016.09.001. PMID: 28220151; PMCID: PMC5315415.  ^2^ Bahiru et al., “Human and Animal Anthrax in Ethiopia: A Retrospective Record Review 2009-2013,” Ethiopian Veterinary Journal 20, no. 2 (2016): 76–85, https://doi.org/10.4314/evj.v20i2.6.  ^3^ (*Anthrax \| CDC*, 2019)  ^4^Maddah, G., Abdollahi, A., & Katebi, M. (2013). Gastrointestinal anthrax: Clinical experience in 5 cases. *Caspian Journal of Internal Medicine*, *4*(2), 672–676. <https://www.ncbi.nlm.nih.gov/pmc/articles/PMC3755822/>  ^5^Kamal SM, Rashid AK, Bakar MA, Ahad MA. Anthrax: an update. Asian Pac J Trop Biomed. 2011;1(6):496-501. doi:10.1016/S2221-1691(11)60109-3 | |

## ***Clostridium botulinum***

*C. botulinum* is an anaerobic spore-forming Gram-positive bacterium that can produce toxins when growing in foods. Foods that are inappropriately processed or preserved through home canning or home-bottling are the most likely to provide an environment where. *C. botulinum* is able to grow and produce toxins^1^. The disease caused by exposure to these toxins is called botulism.

***All text in quotation marks was pulled directly from the 2015 WHO Estimates of the Global Burden of Foodborne Disease ***

| **Rationale for Inclusion** | *C. botulinum* was recommended for inclusion by participants at the TARTARE Scoping Workshop held in March 2020. There is no literature or data identified with estimates of foodborne botulism in Ethiopia. | |
| --- | --- | --- |
| **Overview of Available Data** | **The data included here for *Clostridium botulinum* was taken from the 2015 FERG WHO Estimates of the global burden of foodborne disease^2^.** This data was collected and analyzed, but ultimately *Clostridium botulinum* was removed from the global estimates because **data was only available for high-income countries.** | |
| **Proportion foodborne** | Assumed to be 100% as there is no other known transmission route for gastrointestinal exposure to botulism. Botulism can also occur through inhalation or cutaneous contact. | |
| **Identified Health Outcomes & Disability Weights** | “Botulism (mild to moderate): GBD2010 disability weight 0.198 (95% UI 0.137–0.278) for multiple sclerosis, mild.” | “Botulism (severe): GBD2010 disability weight 0.445 (95% UI 0.303–0.593) for multiple sclerosis, moderate [82].” |
| **Incidence** | “Estimates of incidence were only conducted for the 61 EUR and other subregion A (low mortality) countries. Based on a literature review for articles with national estimates of foodborne diseases including botulism, we identified national estimates of the incidence of botulism from five countries: Canada [175], France [174], Georgia [269], Poland [270] and the United States of America [188]. The median botulism incidence from these five countries was from Canada, therefore the botulism incidence from Canada (0.04 per 100 000 population, with a 90% confidence interval of 0.02–0.08 per 100 000) was used as the incidence for all 55 countries in EUR and AMR A.” | |
| **Percentage of cases by outcome** |  | “[FERG] assumed that 35% (range 20-50%) of botulism cases resulted in severe botulism” |
| **Disease Duration** | ”10 days (min. 5 days–max. 20 days)” | “30 days (min. 15 days–max. 180 days)” |
| **Case fatality rate or ratio** | “Assume no deaths among mild to moderate botulism cases [188, 269, 270].” | “Estimates of mortality were only conducted for the 55 countries in EUR and AMR A. Severe botulism case fatality ratio 15% (range 5-25%).” |
| **Average age of fatal cases** | “Mild to moderate botulism, severe botulism, and botulism death age distribution: mode 50 years (min. age 4 years–max. age 88 years) [269–271].” | |
| **DALYs** | The total number of global DALYS were not calculated in in the 2015 FERG WHO Estimates of the global burden of foodborne disease, but an accompanying FERG paper estimated that globally there are 475 (183-999) foodborne illnesses, 24 (7-65) deaths, and 1,036 (299-2,805) DALYs for an estimate of ~2 DALYs/Case.^3^ | |
| **References**  *Botulism*. (2018, January 10). World Health Organization. https://www.who.int/news-room/fact-sheets/detail/botulism  ^2^  World Health Organization, ed., WHO Estimates of the Global Burden of Foodborne Diseases (Geneva, Switzerland: World Health Organization, 2015).  ^3^ Martyn D. Kirk et al., “World Health Organization Estimates of the Global and Regional Disease Burden of 22 Foodborne Bacterial, Protozoal, and Viral Diseases, 2010: A Data Synthesis,” PLOS Medicine 12, no. 12 (December 3, 2015): e1001921, https://doi.org/10.1371/journal.pmed.1001921. | | |

## ***Lathyrus sativus***

*Lathyrus sativus* commonly known as the grass pea is a leguminous crop that is commonly grown and consumed in part of Northern India and Ethiopia. The neuroexcitatory compound, β-ODAP (β-N-oxalyl-l-α,βdiaminopropionic acid) is essential to *L. sativus’* biosynthesis and therefore present in the crop when it is consumed^1^.

| **Support for Inclusion** | When consumed in large quantities it can lead to lathyrism, which is characterized by paralysis, often of the lower limbs. In 1998, it was reported that neurolathyrism was more common when the grass pea accounted for greater than 30% of the caloric intake sustained over a period of 3-4 months^2^. |
| --- | --- |
| **Overview of Available Data** | In Ethiopia, epidemics of lathyrism were observed in the 1980s and 1990s following period of drought. “Spastic paraparesis with abrupt onset has been reported in epidemic outbreaks in Africa. Clinically and epidemiologically it is similar to lathyrism but without any association with consumption of *L. sativus*. This disease is now called konzo. Konzo has been reported only from poor rural communities in Africa; it is characterized by the abrupt onset of an isolated and symmetric spastic paraparesis which is permanent but non-progressive”^4^ |
| **Proportion foodborne** | Neurolathyrism is caused by the ingestion of *Lathysus sativus* (grasspea), so all cases are considered to be foodborne. |
| **Identified Health Outcomes & Disability Weights** | Not available for lathyrism. The disability weight for severe konzo (= 0.377) can be used because it most closely approximates the disease caused by *L. sativus*. |
| **Incidence** | “After an epidemic of neurolathyrism in Northwestern Ethiopia in 1976–77, a survey of 1,011,272 people identified 2600 patients afflicted with this crippling neurodegeneration. Since then, the annual incidence has been low and was estimated at 1.7 per 10 000 population/year. The drought of 1995/96 wiped out most of the food crops except grasspea, *Lathyrus sativus*, in the northern Wello area. This was the prelude to a new epidemic that started in February, 1997. By January, 1998, 2000 patients had developed the disease. This is the third reported epidemic in Ethiopia and Eritrea within 50 years. The disease is never lethal, but affects mainly the most productive section of the rural communities during times of food shortages”^5^ |
| **% of cases by outcome** | No information available |
| **Disease Duration** | Disease can result in ongoing disability and treatment options are limited. A 2011 case report identified two cases of neurolathyrism in males that experienced initial symptoms in their early teens, and disease progressed to a more debilitating form in their late teens or early 20s. |
| **CFR** | N/a |
| **Age of death** | N/a |
| **DALYs** | No global or country estimates of DALYs were identified. |
| **References**  ^1^Yan *et al.*, “Lathyrus sativus (grass pea) and its neurotoxin ODAP,” *Phytochemistry*, vol. 67, no. 2, pp. 107–121, Jan. 2006, doi: 10.1016/j.phytochem.2005.10.022.  ^2^S. Singh and S. L. N. Rao, “Lessons from neurolathyrism: A disease of the past & the future of Lathyrus sativus (Khesari dal),” *Indian J. Med. Res.*, vol. 138, no. 1, pp. 32–37, Jul. 2013, Accessed: May 15, 2020. [Online]. Available: https://www.ncbi.nlm.nih.gov/pmc/articles/PMC3767245/  ^3^Haimanot, T.R., B.M. Abegaz, E. Wuhib, A. Kassina, Y. Kidane, N. Kebede, T. Alemu and P.S. Spencer, “Pattern of Lathyrus sativus (grass pea) consumption and beta-N-oxalyl-α-β-diaminoproprionic acid (β-ODAP) content of food samples in the lathyrism endemic region of northwest ethiopia,” *Nutr. Res.*, vol. 13, no. 10, pp. 1113–1126, Oct. 1993, doi: 10.1016/S0271-5317(05)80736-5.  ^4^ WHO, 2006. Neurological Disorders: Public Health Challenges. https://www.who.int/publications/i/item/9789241563369.  ^5^Getahun, A. Mekonnen, R. Tekle-Haimanot, and F. Lambein, “Epidemic of neurolathyrism in Ethiopia,” *The Lancet*, vol. 354, no. 9175, pp. 306–307, Jul. 1999, doi: 10.1016/S0140-6736(99)02532-5. | |

## **Rift Valley Fever Virus**

Rift Valley fever virus (RVFV) can be transmitted to humans or livestock by mosquitoes or through direct contact with contaminated bodily fluids and tissues of infected animals. This direct contact can occur during slaughter or butchering, while caring for sick animals, during veterinary procedures, and when consuming raw or undercooked animal products. **There is some evidence that humans may become infected with RVF by ingesting the unpasteurized or uncooked milk of infected animals. Because foodborne transmission is uncommon, there is little literature characterizing this exposure and associated outcomes.**

| **Rationale for Inclusion** | As of 2016, Ethiopia had not reported any outbreaks of RVF, but a geographical analyses identified the areas as highly vulnerable to an RVF outbreak^1,2^. |
| --- | --- |
| **Overview of Available Data** | No data on levels of exposure could be found in the literature or through requests to government stakeholders. |
| **Proportion foodborne** | “Individuals who milked and also consumed raw milk had greater odds of RVFV exposure than individuals whose only contact to raw milk was through milking. Increased risks were associated with exposure to milk sourced from cows (p < 0.001), sheep (p < 0.001), and goats (p < 0.001), but not camels (p = 0.98 for consuming, p = 0.21 for milking). Our data suggest that exposure to raw milk may contribute to a significant number of cases of RVFV, especially during outbreaks and in endemic areas, and that some animal species may be associated with a higher risk for RVFV exposure.”^3^ |
| **Identified Health Outcomes & Disability Weights** | Most commonly, people with RVF have either no symptoms or a mild illness that includes fever, weakness, back pain, and dizziness at the onset of illness. It Is typically self-limiting. However, a small percentage of people infected with RVFV develop much more severe symptoms, including ocular disease, meningoencephalitis, or hemorrhagic fever.^4^ |
| **Incidence** | As of 2016, no cases of RVF had been reported in Ethiopia. No data on human incidence in ET was found. A seroprevalence rate of 7.6% (95% CI: 5.29-10.81) was estimate for cattle in the Gambella Region, South West Ethiopia.^5^ Another study identified a seroprevalence of RVF in humans as 13.2% (95% CI: 8.7-18.8) and in cattle as 15.2% (95% CI: 12.7-18.0) in Adadle, Somali Region of Ethiopia^6^.Note: These estimates are likely much higher than the country incidence because of differences in seroprevalence in incidence and the higher proportion of pastoralists in these areas. |
| **Percentage of cases by outcome** | No evidence available. |
| **Disease Duration** | No evidence available. |
| **Case fatality rate or ratio** | **“**The overall case fatality rate is estimated from 0.5 to 2%, but higher mortality rates were recorded, as for example 18% by the Saudi Health Ministry in 2000, around 22% in East Africa, West Africa, South Africa and Madagascar from 2006 to 2010, and 28% in Tanzania in 2007.**”**^7^ |
| **Average age of fatal cases** | The assumption is that there are no fatal cases |
| **DALYs** | Extrapolating from a simulated epidemic, an estimate of roughly 0.029 DALY/ case can be calculated.^1^ |
| **References**  ^1^Kimani, T.; Schelling, E.; Bett, B.; Ngigi, M.; Randolph, T.; Fuhrimann, S. Public Health Benefits from Livestock Rift Valley Fever Control: A Simulation of Two Epidemics in Kenya. *Ecohealth* **2016**, *13* (4), 729–742. <https://doi.org/10.1007/s10393-016-1192-y>.  ^2^Tran, A.; Trevennec, C.; Lutwama, J.; Sserugga, J.; Gély, M.; Pittiglio, C.; Pinto, J.; Chevalier, V. Development and Assessment of a Geographic Knowledge-Based Model for Mapping Suitable Areas for Rift Valley Fever Transmission in Eastern Africa. *PLoS Negl. Trop. Dis.* **2016**, *10* (9). <https://doi.org/10.1371/journal.pntd.0004999>.  ^3^Grossi-Soyster, E. N.; Lee, J.; King, C. H.; LaBeaud, A. D. The Influence of Raw Milk Exposures on Rift Valley Fever Virus Transmission. *PLoS Negl. Trop. Dis.* **2019**, *13* (3), e0007258. <https://doi.org/10.1371/journal.pntd.0007258>.  ^4^Ikegami, T.; Makino, S. The Pathogenesis of Rift Valley Fever. *Viruses* **2011**, *3* (5), 493–519. <https://doi.org/10.3390/v3050493>.  ^5^Asebe, G.; Mamo, G.; Michlmayr, D.; Abegaz, W. E.; Endale, A.; Medhin, G.; Larrick, J. W.; Legesse, M. Seroprevalence of Rift Valley Fever and West Nile Fever in Cattle in Gambella Region, South West Ethiopia. *Vet. Med. Res. Rep.* **2020**, *11*, 119–130. <https://doi.org/10.2147/VMRR.S278867>.  ^6^Ibrahim, M.; Schelling, E.; Zinsstag, J.; Hattendorf, J.; Andargie, E.; Tschopp, R. Sero-Prevalence of Brucellosis, Q-Fever and Rift Valley Fever in Humans and Livestock in Somali Region, Ethiopia. *PLoS Negl. Trop. Dis.* **2021**, *15* (1), e0008100. <https://doi.org/10.1371/journal.pntd.0008100>.  ^7^Javelle, E.; Lesueur, A.; Pommier de Santi, V.; de Laval, F.; Lefebvre, T.; Holweck, G.; Durand, G. A.; Leparc-Goffart, I.; Texier, G.; Simon, F. The Challenging Management of Rift Valley Fever in Humans: Literature Review of the Clinical Disease and Algorithm Proposal. *Ann. Clin. Microbiol. Antimicrob.* **2020**, *19*. <https://doi.org/10.1186/s12941-020-0346-5>. | |

## **Rotavirus**

Rotavirus is transmitted via the fecal-oral route. Person-to-person spread is the most common mode of transmission, though foodborne outbreaks are associated with rotavirus. Contamination of food with fecal material can occur anywhere along the farm-to-fork continuum, including viral contamination at the source. Rotavirus vaccine coverage is estimated to be 56% nationally but varies greatly by region.^1^

| **Overview of Available Data** | Rotavirus is a well-studied disease, and there are several sources of data on health outcomes and disease characteristics of rotaviral enteritis. Rotavirus was one of the diseases identified in the 2015 Estimates of global, regional, and national morbidity, mortality, and etiologies of diarrheal disease: a systematic analysis for the Global Burden of Disease Study 2015. |
| --- | --- |
| **Proportion foodborne** | A 2008 article conducted an expert elicitation attributing 13% (13%- 28%) to rotavirus cases to foodborne transmission in the Netherlands^2^. |
| **Identified Health Outcomes & Disability Weights** | According to the US CDC, rotavirus disease results in a gastrointestinal illness characterized by fever, dehydration, watery diarrhea, and abdominal pain. The disease primarily affects infants; however, adults how are sickened generally have more mild illnesses.^3^ |
| **Incidence** | Not available |
| **% cases by outcome** | Not available |
| **Disease Duration** | Symptoms typically appear around 2 days after exposure to the virus; vomiting and diarrhea can last 3 to 8 days.^3^ |
| **Case fatality rate or ratio** | In 2015, 2587.9 deaths were attributed to Rotavirus in children below 5 years of age in Ethiopia; however, a total DALY specific to Rotavirus in Ethiopia was not presented with the estimates.^4^ |
| **Age of mortality** | Not available |
| **DALYs** | According the IHME GBD Compare Data Visualization Hub.^5^ in Ethiopia in 2015, rotavirus accounted for 9.71% (3.63% to 20.67%) of DALYs attributed to diarrheal diseases and 7.61% (2.71% - 16.53%) of deaths attributed to diarrheal diseases. |
| **References**  Anne Geweniger and Kaja M. Abbas, “Childhood Vaccination Coverage and Equity Impact in Ethiopia by Socioeconomic, Geographic, Maternal, and Child Characteristics,” *Vaccine* 38, no. 20 (April 29, 2020): 3627–38, https://doi.org/10.1016/j.vaccine.2020.03.040.  ^2^ Arie H. Havelaar et al., “Attribution of Foodborne Pathogens Using Structured Expert Elicitation,” *Foodborne Pathogens and Disease* 5, no. 5 (October 2008): 649–59, <https://doi.org/10.1089/fpd.2008.0115>.  ^3^ Learn more about Rotavirus. (2021, March 26). Centers for Disease Control and Prevention. https://www.cdc.gov/rotavirus/index.html  ^4^ GBD Diarrhoeal Diseases Collaborators, “Estimates of Global, Regional, and National Morbidity, Mortality, and Aetiologies of Diarrhoeal Diseases: A Systematic Analysis for the Global Burden of Disease Study 2015,” *The Lancet*. Infectious Diseases 17, no. 9 (2017): 909–48, https://doi.org/10.1016/S1473-3099(17)30276-1.  ^5^ IHME, “GBD Compare Data Visualization Hub,” n.d., https://vizhub.healthdata.org/gbd-compare/. | |

##

## ***Staphylococcus aureus***

Staphylococcal food poisoning is caused by the consumption of preformed staphylococcal enterotoxins (SEs) produced by *Staphylococcus aureus* (SA). Most outbreaks are the result of poor hygiene during processing resulting in transfer of SA or SEs to the food reservoir from human skin or dairy animals with mastitis^1^.

***All text in quotation marks was pulled directly from the 2015 WHO Estimates of the Global Burden of Foodborne Disease ***

| **Rationale for Inclusion in the Risk Ranking** | Exposure to the bacterium SA is well established in Ethiopia. Studies have been conducted in several parts of the country investigating the prevalence of SA in dairy farms, abattoir, and among human food handlers, but this data is not specific to presence of SE^2^. |
| --- | --- |
| **Overview of Available Data** | **The data included here for *Staphylococcus aureus* was taken from the 2015 FERG WHO Estimates of the global burden of foodborne disease^3^**. This data was collected and analyzed, but ultimately *Staphylococcus aureus* was removed from the global estimates because **data was only available for high-income countries.** |
| **Proportion foodborne** | Assumed to be 100% because staphylococcal food poisoning requires multiplication of the bacteria in food. Note: not all SA strains produce SEs; SEs production at toxic levels when SA level is over 10^5^ cfu/g. |
| **Identified Health Outcomes & Disability Weights** | “Clinical outcomes were acute gastroenteritis due to *S. aureus* intoxication and death due to *S. aureus* intoxication [273].” “Acute gastroenteritis due to *S. aureus* intoxication: GBD2010 disability weight 0.061 (95% UI 0.036–0.093) for diarrhea, mild [82].” |
| **Incidence** | “ Estimates of incidence were only conducted for the 61 EUR and other subregion A (low mortality) countries. Based on a literature review for articles with national estimates of foodborne diseases that included *Staphylococcus aureus* intoxication, we identified national incidence estimates for S. aureus intoxication from seven countries: Australia [272], Canada [175], France [174], Netherlands [154], New Zealand [252], England and Wales as a proxy for United Kingdom [172], and the United States of America [188]. The median S. aureus intoxication incidence WHO Estimates of the global burden of foodborne diseases 153 from these seven countries was from Canada, therefore the *S. aureus* intoxication incidence from the Canada (77.3 per 100 000 population with a 95% confidence interval of 50.65–118.0 per 100 000) was used as the *S. aureus* intoxication incidence for all EUR and other subregion A countries” |
| **Disease Duration** | “Acute gastroenteritis due to *S. aureus* intoxication: duration 1 day (min. 0.25 days–max. 2.5 days) [273].” |
| **Mortality** | “Estimates of mortality were only conducted for the 61 EUR and other subregion A (low mortality) countries. National estimates of *S. aureus* intoxication cases and deaths were available from the Netherlands [154] and the United States of America [188]; the case fatality ratio (CFR) for the Netherlands was 0.0024% and for the United States of America was 0.0025%. We used the CFR from the United States of America as the CFR for all EUR and other subregion A countries with a 95% confidence interval of 0.0012%-0.0045%.” |
| **Average age of fatal cases** | **“**Acute gastroenteritis and deaths due to *S. aureus* intoxication age distribution: 5% <5 years; 19% 5–14 years; 48% 15–54 years; 28% >55 years**”** |
| **DALYs** | The total number of global DALYs was not calculated in WHO FERG report, but an accompanying FERG paper^4^ provided global estimates of 1,073,339 (658,463-1,639,524) cases, 25 (10-55) deaths, and 1,575 (702-3,244) DALYS, for an estimate of ~ 0.001 DALYs/case. |
| **References**  Jacques-Antoine Hennekinne et al., “How Should Staphylococcal Food Poisoning Outbreaks Be Characterized?,” *Toxins* 2, no. 8 (August 10, 2010): 2106–16, https://doi.org/10.3390/toxins2082106.  ^2^ Yodit Ayele et al., “Assessment of Staphylococcus Aureus along Milk Value Chain and Its Public Health Importance in Sebeta, Central Oromia, Ethiopia,” *BMC Microbiology* 17, no. 1 (27 2017): 141, https://doi.org/10.1186/s12866-017-1048-9; Takele Beyene et al., “Prevalence and Antimicrobial Resistance Profile of Staphylococcus in Dairy Farms, Abattoir and Humans in Addis Ababa, Ethiopia,” BMC Research Notes 10, no. 1 (April 28, 2017): 171, https://doi.org/10.1186/s13104-017-2487-y; Getenet Beyene et al., “Nasal and Hand Carriage Rate of Staphylococcus Aureus among Food Handlers Working in Jimma Town, Southwest Ethiopia,” Ethiopian Journal of Health Sciences 29, no. 5 (September 2019): 605–12, https://doi.org/10.4314/ejhs.v29i5.11.  ^3^ World Health Organization, ed., WHO Estimates of the Global Burden of Foodborne Diseases (Geneva, Switzerland: World Health Organization, 2015). | |

## ***Taenia saginata***

*Taenia saginata* is an intestinal cestode (tapeworm) that lives in the intestines of humans as the definitive host. The larval stage occurs in tissues of cattle, as the intermediate host, and were previously known as *Cysticercus bovis*. Consuming infected meat can lead to human infection.

| **Rationale for Inclusion** | Cysticercosis / taeniasis was ranked the #11 priority zoonotic disease in Ethiopia in a risk ranking exercise conducted by the US CDC, EPHI and various other organizations^1^. There are no known fatalities, but infection presents a quality control issue because of meat inspection requirements. The European Food Safety Agency has categorized T. saginata a low priority for bovine meat inspection. |
| --- | --- |
| **Overview of Available Data** | A 2019 publication summarized data from questionnaires on the distribution of human taeniasis over the years 2011-2018, providing a starting point to generate exposure estimates^2^. |
| **Percent of cases considered foodborne:** | Because infections result from the consumption of undercooked beef, 100% of cases are assumed to be foodborne. |
| **Identified Health Outcomes & Disability Weights** | According to the US CDC: “ Most people with tapeworm infections have no symptoms or mild symptoms. Patients with *T. saginata* taeniasis often experience more symptoms that those with *T. solium or T. asiatica* infections because the *T. saginata* tapeworm is larger in size (up to 10 meters (m)) than the other two tapeworms (usually 3 m). Tapeworms can cause digestive problems including abdominal pain, loss of appetite, weight loss, and upset stomach. The most visible sign of taeniasis is the active passing of proglottids (tapeworm segments) through the anus and in the feces. In rare cases, tapeworm segments become lodged in the appendix, or the bile and pancreatic ducts.” “*Taenia saginata* does not cause cysticercosis in humans.”^3^ |
| **Incidence** | Self-reported prevalence on human taeniasis ranged from 7.8% on Mojo, Oromia Region to 89.4% in Addis Ababa City, Addis Ababa.^5^ Additionally, a 2020 report estimated prevalence ranging from 0.6 to 10.7%^6^. |
| **Percentage of cases by outcome** | N/a |
| **Disease Duration** | Taeniasis can persist for 2-3 years^3^. |
| **Case fatality rate or ratio** | Very few cases lead to death. |
| **Average age of fatal cases** | N/a |
| **DALYs** | No global or country DALY estimates were identified. |
| **References**  (Pieracci et al., 2016)  2 (Hiko & Seifu, 2019)  ^3^ (*CDC - Taeniasis - General Information - Frequently Asked Questions (FAQs)*, 2020)  ^4^ (Salomon et al., 2015)  ^5^ (Hiko & Seifu, 2019)  ^6^ Jorga, Edilu, Inge Van Damme, Bizunesh Mideksa, and Sarah Gabriël. 2020. “Identification of Risk Areas and Practices for Taenia Saginata Taeniosis/Cysticercosis in Ethiopia: A Systematic Review and Meta-Analysis.” *Parasites & Vectors* 13 (1): 375. [https://doi.org/10.1186/s13071-020-04222-y](https://urldefense.com/v3/__https:/doi.org/10.1186/s13071-020-04222-y__;!!KGKeukY!zorvUpIrPIBnvVyitbDl7VGE4eHKiyEo_s0RtVbCEydz6c-54NL3pAaT0M_qW9eZmK9vHstGDh12xVf8kCvp5jzBEwxneEEL4eHaMg$). | |

# **Appendix C: Expert Feedback**

## *Bacillus anthracis*

| **Assumptions** | **Respondent A** | **Respondent B** | **Respondent D** | **Respondent E** | **Respondent F** |
| --- | --- | --- | --- | --- | --- |
| Incidence  % of anthrax cases that are GI  Lower = 1%  Midpt = 5%  Upper = 20% | Yes | Yes | Incidence low from eating meat infected with bacteria. 5% is a good estimate. | We do have anthrax outbreaks. Gondar. Pastoral areas. Culture – when an animal is about to die, they slaughter and eat. | Spores can also be acquired from animals. Co-living with animals.  Agree. Maybe a little higher. |
| Average age of death/onset = 20 | No  Lower = 20  Midpt = 40  Upper = 60  Rationale: If that was an old data I agree, Ethiopia has reported significant improvement on that recently which needs to be considered | No  Lower = 5  Midpt = n/a  Upper = 60  Rationale: It has an acute pathogenesis in most of the cases | Did not know. Mentioned EPHI case study article – I have asked for this. | Not sure. Will look for life expectancy in pastoral areas. | Agree |
| Mortality  Annual number of deaths  Lower = 0  Midpt = 0.2  Upper = 0.9 | No  Total deaths per year  Lower = 10  Midpt = 40  Upper = 75  Rationale: The small number reported is part of the health system problem in properly detecting, diagnosing, and reporting. There will for sure be a lot more missing cases | No  CFR < 0.5% | n/a | n/a | n/a |
| Assume all burden from death? | No  Estimate provided = 0  Rationale: the basis for the assumptions are old data and I don't think this is a major problem now | Yes | Agree | Not sure | Agree |

## *Clostridium botulinum*

| **Assumptions** | **Respondent A** | **Respondent B** | **Respondent D** | **Respondent E** | **Respondent F** |
| --- | --- | --- | --- | --- | --- |
| Incidence^[[1]](#footnote-2)^  Lower bd = 0.02 per 100k or 20 per year in ET  Midpt = 0.04 per 100k or 40 per year in ET  Upper = 0.08 per 100k or 80 per year in ET | Yes | Yes | Should be lower. We don’t eat processed and canned foods like the Western countries these estimates are based on. 80% of population lives in rural areas and only eats fresh food. This is not a good reference for ET. | Should be lower. Mostly consume home-cooked food. Canned food is only consumed in cities and even there is low. | There is not much info on this. I have seen one case report on 14 cases.  Note: I requested this report and will share when I receive it. |
| Mortality^1^  Lower bd = 0.018 deaths per case  Midpt = 0.053 deaths per case  Upper = 0.088 deaths per case | [No response] | Yes | No health system. No medical infrastructure. This should be reported as number of deaths. There is an EPHI case study outbreak in Walega. 50% CFR. Paper attached. The mortality rate should be higher. | Most FBD is undiagnosed. Do not seek medical help. These cases would not be identified and treated. Eg., From our work on brucellosis -no diagnosis. Would have higher CFR than Western. | Agree |
| DALY^1^  Lower bd = 0.53 DALYS per case  Midpt = 2.4 DALYS per case  Upper bd = 8.7 DALYS per case | [No response] | Yes | Would expect that Western would be higher than ET with good clinical care | Would be higher | agree |

## *Lathyrus sativus*

| **Assumptions** | **Respondent A** | **Respondent B** | **Respondent D** | **Respondent E** | **Respondent F** |
| --- | --- | --- | --- | --- | --- |
| Incidence  Lower bd = 8,500 per year  Midpt = 17,000 per year  Upper bd = 34,000 per year | Yes | Yes | We heard about in childhood. I have never seen a case. Northern central. Limited area. Now well understood risk. Assume lower. | Only use when drought. Will seek demographic info on # of people who live in areas where this crops grows. | No response – he’s a microbiologist |
| Age of onset  20 years old | 30  Rationale: Ethiopian population average life expectancy has changed from 40 to 60 years since the last study on Lathyrus Sativus is conducted which will have an impact on the age range |  | Unknown |  |  |
| Mortality  CFR = 0 | Yes | No  CFR < 0.5% | Agree | Agree |  |
| Disability weight  0.377 | No  Estimate provided = 0  Rationale: the basis for the assumptions are old data and I don't think this is a major problem now | Yes | Agree | No comment | No comment |

## Rift Valley Fever Virus

| **Assumptions** | **Respondent A** | **Respondent B** | **Respondent C** | **Different framing of estimates** | **Respondent D** | **Respondent E** | **Respondent F** |
| --- | --- | --- | --- | --- | --- | --- | --- |
| Incidence  Lower bound: 0 cases  Midpoint: 0.001 cases per 100,000  Upper bound : 0.005 cases per 100,000 | No response | Yes | No  Lower = 3%  Midpt = [no value given]  Upper = 12%  Rationale: Ethiopia has a large pastoralist community being exposed significantly to the disease | # of RVF cases in ET annually  Lower bd = 0.1  Best estimate = 2  Upper bd = 5 | None reported since 2016. Does not mean no cases. Could be a limitation of lab capacity. Could be more. Would want to be higher. | Cannot assess. Large population is living in pastoral area. | RVF not reported, not isolated. We know it is in Kenya and Sudan. We do have sero-prevalence data. Agree with these estimates. |
| Mortality  Assumed 0 | No  [no estimate provided] | No  Total annual deaths  Lower = 0  Midpt = 50  Upper = 100 | No  CFR < 0.01 |  | Can be cured. Self-limiting. Endemic in areas with poor infrastructure. A few deaths – make a very small number. Some papers estimate 22%. | I don’t know the natures of the disease. We share a large border with Kenya where it is common in pastoral com-munities | Agree with 0 |
| DALY  DALY/case = 0.029 | No | Yes | Yes | n/a | n/a | n/a | n/a |
| Proportion foodborne  Lower = 0  Midpt = 0  Upper = 1% | No  [no estimate provided] | Yes | Yes | Lower 0.1%  Midpt 1%  Upper 5% | Agree, mostly from mosquito bites | agree | agree |

## Rotavirus

| **Assumptions** | **Respondent A** | **Respondent B** | **Respondent D** | **Respondent E** | **Respondent F** |
| --- | --- | --- | --- | --- | --- |
| Total Incidence rate for Rotavirus (not only foodborne)  Lower = 2000 per 100,000  Mid = 7000 per 100,000  Upper = 1,700,000 per 100,000 | Yes | Yes | n/a | n/a | n/a |
| Total Rotavirus Mortality (not only foodborne)  Lower = 889  Midpt = 3560  Upper = 10,509 | Yes | No /  Lower = 10,000  Midpt = 15,000  Upper = 25,000 | n/a | n/a | n/a |
| Total DALYs for rotavirus(not only foodborne)  Lower: 64,000 DALYs  Midpoint: 240,000 DALYs  Upper: 686,000 DALYs | Yes | No  Lower = 110,000  Midpt = 500,000  Upper = 10,000,000 | n/a | n/a | n/a |
| Attribution to food  Lower bound: 0%  Midpoint estimate: 13%  Upper bound: 28% | No  5/12/18  Rationale: We can't translate the finding in Netherlands to Ethiopia as there are significant difference in a number of parameters. Vaccination status, the year the study was done, the availability exposure factors, co morbidities, health seeking behavior of individuals. | No  10/20/40  diarrhea caused by the Rota virus is rampant in Ethiopia | Would want to use a country with similar economic status. Since driven by personal hygiene and food handling. Would assume much higher for ET. Assume this is mostly children under 5.  Provided attached info sheet on rotavirus vaccines | We do not have much information on prevalence or on foodborne.  This assumption is good since the nature of rotavirus is fecal-oral and we have low sanitation | Agree. Most transmission is person-to-person. |

## *Staphylococcus aureus*

| **Assumptions** | **Respondent A** | **Respondent B** | **Respondent C** | **Respondent D** | **Respondent E** | **Respondent F** |
| --- | --- | --- | --- | --- | --- | --- |
| Incidence^[[2]](#footnote-3)^  Lower bound: 51 per 100,000  Midpoint: 77 per 100,000  Upper bound : 120 per 100,000 | No  Lower bound =20 per 100k  Best = 30 per 100k  Upper = 40 per 100k Rationale: [None provided] | No  [No estimates provided] | Yes | Hygiene and safe handling play a role. Would expect ET is higher. | With Staph A, toxin is synthesized by bacteria. Milk, cheese, yogurt. Many population pastoral and high land. Cultural habit to consume raw milk. Ambient temp for days, releases more toxin. I would assume more cases per year in ET. | Agree |
| Mortality ^[[3]](#footnote-4)^  CFR of 0.0025% (range 0.0012%- 0.0045%) | No  [No estimate provided] | Yes | Yes | Agree. Not as high as C. Bot | Agree. Self-limiting | Agree |
| DALY  (2015 WHO Estimate)  average DALY per case was 0.0015 with an uncertainty range of (0.0006, 0.0035) | No  [No estimate provided] | Yes | Yes | Agree | Agree | Agree |

## *Taenia saginata*

| **Assumptions** | **Respondent A** | **Respondent D** | **Respondent E** | **Respondent F** |
| --- | --- | --- | --- | --- |
| Incidence  Lower bd = 2,700,000 per year  Midpt = 10M per year  Upper = 30M per year | Yes | From raw meat. 35-45% of population is Muslim and does not eat raw meat. Only northern central and some big cities eat raw meat. More than 80% are rural and only eat meat 1-2 days per year. Therefore this number is too large. Maybe 8%. Need to estimate % that eat raw meat; not all of them will get TS. | Cultural habit to eat raw meat. These estimates are good. Would be different in highlands. Most are orthodox Christian. There is less consumption of meat. More in pastoral areas. Will look for demographic data on % Muslim, % pastoral for 2010. | It’s very common to eat raw meat.  For example, kifto and dullet (?)  These numbers should be higher.  Lower = 10M  Midpt = 20M  Upper = 40M |
| Duration of illness  3 years | No, 1 year  I am suggesting that based on my scientific and clinical experience | 3 years is too long | n/a | n/a |
| Mortality  CFR = 0 | No  CFR should be 0.001  Mainly because of complications including malabsorption, appendicitis and Cysticercosis following the infection | Agree. Traditional medicine is used to treat. | Agree. Indigenous people have cultural medications to treat. Do not seek healthcare. They can de-worm. They know when they are infected. | Agree |
| Disability weight  Lower = 0.006 mild nausea  Midpt = 0.04  Upper = 0.074 severe nausea | Yes | Agree | Upper bound may be lower.  Usually no symptoms. Freq exposure leads to less impact. | Agree. Do not expect symptoms |

1. From WHO estimates using data from Canada, France, Georgia, Poland, and U.S. [↑](#footnote-ref-2)
2. From WHO estimates using data from Canada, France, Georgia, Poland, and U.S. [↑](#footnote-ref-3)
3. 2015 WHO Estimate [↑](#footnote-ref-4)
