# Supplementary material for: A Proposed Framework for Ranking and Prioritizing Food Safety Risks in Low Resource Settings Using Foodborne Disease Burden Metrics: A Case Study in Ethiopia
Source: J Food Prot. 2025 Jun 23;88(7):100525. doi: 10.1016/j.jfp.2025.100525 (PMC12548780; doi:10.1016/j.jfp.2025.100525)
Supplement: Supplementary Appendix C [file mmc3.pdf]

Risk ranking technical appendix

2025-05-28

Table of contents

1 Introduction **3**

2 Disease burden estimates **3**

3 Burden calculations non-FERG hazards in `R` **4**

3.1 Functions 4

3.1.1 Function code and fixed inputs 6

3.1.2 Fixed inputs 6

3.2 Burden estimation 6

3.2.1 Acrylamide 6

3.2.2 Aflatoxin M1 6

3.2.3 *Bacillus anthracis* 6

3.2.4 *Clostridium botulinum* toxins 6

3.2.5 *Lathyrus sativus* 6

3.2.6 Rift Valley Fever virus 6

3.2.7 Rotavirus 6

3.2.8 *Staphylococcus aureus* toxins 6

3.2.9 *Taenia saginata* 6

3.2.10 Results 6

4 Burden calculations non-FERG hazards in `Excel` **8**

4.1 Multiplying or dividing distributions 8

4.2 Beta distributions 11

4.3 Gamma distributions 11

5 Disease burden dashboard **12**

5.1 Data 13

5.1.1 Data Collection and Usage 13

5.1.2 Risk Metric Definitions 13

5.2 Features . . . 14
5.2.1 All Hazards Tab . . . 14
5.2.2 FERG Hazards . . . 16
5.2.3 Scatter Plot . . . 16
5.2.4 Weighted Scatter Plot . . . 16
5.2.5 Add New Hazards . . . 17

6 Risk ranking 19
6.1 Methods . . . 19
6.2 Results . . . 19
6.2.1 Descriptive analysis . . . 19
6.2.2 Ordinal logistic regression . . . 21

7 Attribution of foodborne deaths to hazards 24
7.1 The Delphi method . . . 24
7.2 Round 1 . . . 25
7.2.1 Elicitation instrument . . . 25
7.2.2 Data . . . 27
7.2.3 Average expert estimates . . . 27
7.3 Expert agreement . . . 27
7.4 Expert rationale . . . 30
7.5 Evaluation Round 1 . . . 30
7.6 Round 2 . . . 31
7.6.1 Instructions . . . 31
7.6.2 Data . . . 31
7.6.3 Updated expert estimates . . . 31
7.7 Attribution to food groups . . . 32
7.8 Updated attributable deaths . . . 33

8 Prioritization 33
8.1 Foodborne deaths attributable to Supply Chain Control Points in four food value chains . . . 33
8.2 Supply Chain Control Points . . . 35

References 39

Appendix A. Expert rationale in Round 1 43
Aflatoxin B1 . . . 43
Arsenic . . . 43
Campylobacter . . . 44
EPEC . . . 45
ETEC . . . 45
Salmonella . . . 46

Norovirus . . . 46
Rotavirus . . . 46
S. Typhi . . . 47
Shigella . . . 47
V. cholerae . . . 48

1 Introduction

This document provides detailed information on the data analysis and visualization of results from the workshops with governmental decision makers and relevant stakeholders to identify, rank and prioritize food safety hazards in Ethiopia as described in the manuscript "A proposed framework for ranking and prioritizing food safety risks in low resource settings using foodborne disease burden metrics: a case study in Ethiopia" by B. Kowalcyk *et al*.

Code and data are available on GitHub for the dashboard and technical appendix. The latter repository also includes a pdf file with all code visible.

2 Disease burden estimates

The workshops have been informed by comprehensive estimates of the burden of foodborne disease in Ethiopia. With permission from the Ethiopian government, data on disease incidence, mortality and Disability-Adjusted Life Years (DALYs) for 31 hazards were extracted from the World Health Organization (WHO) Foodborne Disease Burden Epidemiology Reference group (FERG) (*4*). The burden of heavy metals was based on estimates published by Gibb *et al.* (*2*). Monte Carlo sample data of both datasets were kindly provided by Dr. Brecht Devleesschauwer, Sciensano, Brussels, Belgium.

Mean estimates and 95% uncertainty intervals were calculated for each of the selected risk metrics for hazards not considered by WHO FERG. When available, data were extracted from published literature. In the absence of published data, assumptions were made, reviewed with Ethiopian experts and, when appropriate, adjusted. A standardized method was used to estimate uncertainty for each input. Whenever possible, uncertainty estimates were computed from reported data. If no data were available to assess uncertainty, broad uncertainty was assumed. For age of onset, uncertainty bounds were assumed to be ± 20 years of the midpoint. In all other cases, the midpoint was, as appropriate, divided or multiplied by 10 to obtain lower and upper uncertainty bounds. If data were available, uncertainty in proportions (e.g., case-fatality ratio) was modeled as a Beta distribution while uncertainty in rates was modeled with a Gamma distribution (*11*).

Once inputs and assumptions were finalized, an approximate analytical approach was used to calculate best estimates and uncertainty intervals for each risk metric for each pathogen. In

many cases, this involved multiplying or dividing two uncertainty distributions. If samples from these distributions were available, this could be achieved using Monte Carlo simulations. In many cases where only a mean estimate and 95% uncertainty interval were available, we assumed uncertainty could be modeled using lognormal distributions.

Since the FERG estimates are the most recent available global estimates, 2010 was used as the reference year for all burden estimates.

3 Burden calculations non-FERG hazards in `R`

3.1 Functions

Input data for calculating the disease burden of non - FERG hazards were uncertainty distributions defined by three characteristic values: *lower* and *upper* bounds representing the 95% uncertainty interval, and a *mean* estimate. Disease burden calculations involved multiplying or dividing two uncertainty distributions: Approximate calculations assuming lognormal uncertainty distributions were performed in `Excel` spreadsheets and in `R` (*6*). This section provides the theoretical background and the `R` code.

$$d_{12} = d_1 \times d_2 \;\; or \tag{1}$$

$$d_{12} = d_1 / d_2 \tag{2}$$

Taking logarithms gives:

$$log(d_{12}) = log(d_1) + log(d_2) \;\; or \tag{3}$$

$$log(d_{12}) = log(d_1) - log(d_2) \tag{4}$$

The mean of a normal distribution with known upper and lower bounds is the average of these bounds, and the width of the 95% uncertainty interval is two times 1.96 standard deviations:

$$m = \frac{lower + upper}{2} \tag{5}$$

$$sd = \frac{upper - lower}{2 * 1.96} \tag{6}$$

Equation 5 can be used to partly check the lognormal assumption of the data, as the symmetry of a normal distribution requires the calculated mean to be close to the specified *mean*.

The mean and standard deviation of the sum or difference of two uncorrelated normal distributions are calculated as follows:

$$log(d_1) \sim N(m_1, sd_1) \;\; and \tag{7}$$

$$log(d_2) \sim N(m_2, sd_2) \tag{8}$$

$$log(d_1) + log(d_2) \sim N(m_1 + m_2, \sqrt{sd_1^2 + sd_2^2}) \;\; or \tag{9}$$

$$log(d_1) - log(d_2) \sim N(m_1 - m_2, \sqrt{sd_1^2 + sd_2^2}) \tag{10}$$

Finally, the mean and 95% uncertainty interval on the original scale are calculated by back-transformation from the mean m and standard deviation sd on the log scale:

$$log(lower) = m - 1.96 * sd \tag{11}$$

$$log(upper) = m + 1.96 * sd \tag{12}$$

$$mean = exp^{m+sd^2/2} \tag{13}$$

$$lower = exp^{log(lower)} \tag{14}$$

$$upper = exp^{log(upper)} \tag{15}$$

Here, exp is the base of the logarithm used to transform the data, typically e or 10.

The assumption of uncorrelated distributions is reasonable for virtually all calculations that involve multiplications. In several calculations that involve divisions, this assumption is less defensible and not accounting for correlation will lead to larger estimates of the confidence intervals. In the absence of data on correlation coefficients, we accept this conservative approach.

3.1.1 Function code and fixed inputs

3.1.2 Fixed inputs

We define life expectancy as 90 years for men and women in accordance with WHO FERG. The population size of Ethiopia in 2010 was 87,640,000 (*10*).

3.2 Burden estimation

This section shows code and results for the burden estimation of non-FERG hazards. The code is not visible in this document but can be accessed in the pdf with code in the Github repository.

3.2.1 Acrylamide

3.2.2 Aflatoxin M1

3.2.3 *Bacillus anthracis*

3.2.4 *Clostridium botulinum* toxins

3.2.5 *Lathyrus sativus*

3.2.6 Rift Valley Fever virus

3.2.7 Rotavirus

3.2.8 *Staphylococcus aureus* toxins

3.2.9 *Taenia saginata*

3.2.10 Results

3.2.10.1 Inputs and evaluation of lognormal assumption

Table 1 shows the input data and an evaluation of the lognormal assumption for the non-FERG hazards, which is reasonably well met for most inputs.

Table 1: Inputs and symmetry of log - transformed data

Hazard	Variable	Lower Bound	Mean	Upper Bound	Symmetry[1]
Acrylamide	Proportion_AA	0.000021	0.00021	0.0021	1.0
Acrylamide	Cancer_Mortality	270	1,300	3,900	0.97
Acrylamide	Cancer_Incidence	410	2,000	5,300	0.96
Aflatoxin M1	DALYs_per_Case	31	33	35	1.0
Aflatoxin M1	AFB1_Deaths	160	380	910	1.0
Aflatoxin M1	AFB1_Cases	180	430	1,000	1.0
Aflatoxin M1	AFM1_Cases_per_100000	0.00079	0.0025	0.0079	1.0
Bacillus anthracis	DALYs	23	78	230	0.98
Bacillus anthracis	Age_of_Onset	30	50	70	0.98
Bacillus anthracis	Case - Fatality Ratio	0.013	0.017	0.020	1.0
Bacillus anthracis	Incidence	1,000	1,000	1,100	1.0
Clostridium botulinum	Case - Fatality_Ratio	0.10	0.30	0.50	1.2
Clostridium botulinum	Proportion Severe	0.20	0.35	0.50	1.1
Clostridium botulinum	Incidence per 100000	0.020	0.040	0.080	1.0
Clostridium botulinum	Global DALYs	300	1,000	2,800	0.98
Clostridium botulinum	Global Incidence	180	480	990	0.98
Lathyrus sativus	Disability weight	0.0038	0.038	0.38	1.0
Lathyrus sativus	Age of onset	5.0	20	40	0.88
Lathyrus sativus	Incidence per 100000	0.017	0.17	1.7	1.0
Rift Valley Fever	RVF_DALYS_per_Case	0.0029	0.029	0.29	1.0
Rift Valley Fever	Case_Fatality_Ratio	0.0030	0.010	0.020	1.1
Rift Valley Fever	Proportion_foodborne	0.0010	0.010	0.050	1.1
Rift Valley Fever	Total_RVF_Cases	0.10	2.0	5.0	-0.50
Rotavirus	Rotavirus_DALYs	170,000	490,000	1,100,000	0.99
Rotavirus	Rotavirus_Mortality	2,000	5,800	12,000	0.98
Rotavirus	Proportion_foodborne	0.050	0.13	0.28	1.0
Rotavirus	Proportion_DALYs_Rotavirus	0.053	0.12	0.20	1.1
Rotavirus	Total_Diarrheal_Cases	88,000,000	97,000,000	110,000,000	1.0
Staphylococcus aureus toxins	Global Cases	660,000	1,100,000	1,600,000	1.0
Staphylococcus aureus toxins	Global DALYs	700	1,600	3,200	0.99
Staphylococcus aureus toxins	Case - Fatality Ratio	0.0024	0.0050	0.0090	1.0
Staphylococcus aureus toxins	Incidence per 100000	51	77	120	1.0
Taenia saginata	Proportion_symptomatic	0.35	0.35	0.35	1.0
Taenia saginata	Disability_Weight	0.0060	0.011	0.040	0.92
Taenia saginata	Duration	0.30	3.0	30	1.0
Taenia saginata	Prevalence	0.016	0.019	0.022	1.0

[1]Symmetry is calculated as the ratio of the mean of the lower and upper bounds to the specified mean. A value of 1 indicates perfect symmetry, while values < 1 indicate right skewness and values > 1 indicate left skewness.

3.2.10.2 Foodborne disease burden estimates

Table 2 shows the burden estimates for the non-FERG hazards by risk metric.

4 Burden calculations non-FERG hazards in `Excel`

The workbook `Burden_calc_approx.xlsx` provides approximate models to multiply or divide uncertainty distributions to support calculations of the burden of foodborne disease when few data are present in a spreadsheet format. Spreadsheets are also provided to calculate the uncertainty distributions of proportions and rates, using Beta and Gamma distributions, respectively. The workbook presents empty spreadsheets where users can enter data, as well as worked examples.

4.1 Multiplying or dividing distributions

The approximate models are based on the assumption that the distributions to be multiplied or divided are lognormal. Lognormal distributions are flexible and allow us to model data with long right tails. These distributions cannot assume negative values, which is realistic for all parameters included in burden calculations. Analytical formulas are available to perform the multiplication or division of normal distributions and are implemented in the spreadsheets.

Figure 1 illustrates the calculations for multiplying two distributions using mortality from AFM1 as an example. Inputs are the incidence of liver cancer due to exposure to AFM1 and the case-fatality ratio. Users enter descriptors of the inputs and output in cells C4:D4 and C17, and data in the cells C5:D7. Lower bounds in cells C5:D5 and upper bounds in cells C7:D7 are assumed to represent 95% uncertainty intervals. The mean values entered in cells C6:D6 are not used in the calculations, they are used to check if the lognormal assumption is realistic for both input distributions. If so, the results in cells E6:F6 should be approximately the same as the input means. If means are not available, medians can be entered but a larger difference with the check values is expected.

Calculations are performed in two steps. In the first step (cells C11:D14), upper and lower bounds are transformed to the log scale and parameters of the distributions are calculated. In the second step (cells C18:C22), the multiplication or division is carried out as addition or subtraction on the log scale. The results are obtained by back-transformation of the calculation results in cells C26:C28. A second check is included by providing the product or quotient of the means of the input distributions (cell E27). This value should be approximately the same as the calculated mean.

The calculations for dividing two distributions are the same as for multiplying, except that in cell C18, the mean logs of the two input distributions are subtracted instead of added and in cell D27, the input means are divided instead of multiplied.

Table 2: Foodborne disease burden estimates non - FERG hazards by risk metric

Hazard Name	Risk Metric	Lower Bound	Mean	Upper Bound
Acrylamide	Incidence	0.022	0.46	4.3
Acrylamide	Incidence__per__100000	0.000025	0.00052	0.0049
Acrylamide	Mortality	0.015	0.32	3.1
Acrylamide	Mortality__per__100000	0.000017	0.00037	0.0035
Acrylamide	Case__Fatality__Ratio	0.055	0.54	0.97
Acrylamide	DALYs	0.51	11	99
Acrylamide	DALYs__per__100000	0.00058	0.012	0.11
Acrylamide	DALYs__per__Case	23	23	23
Aflatoxin M1	Incidence	0.69	2.2	6.9
Aflatoxin M1	Incidence__per__100000	0.00079	0.0025	0.0079
Aflatoxin M1	Mortality	0.60	2.1	6.0
Aflatoxin M1	Mortality__per__100000	0.00069	0.0023	0.0069
Aflatoxin M1	Case__Fatality__Ratio	0.84	0.87	0.90
Aflatoxin M1	DALYs	23	78	230
Aflatoxin M1	DALYs__per__100000	0.026	0.089	0.26
Aflatoxin M1	DALYs__per__Case	31	33	35
Bacillus anthracis	Incidence	10	53	210
Bacillus anthracis	Incidence__per__100000	0.012	0.060	0.24
Bacillus anthracis	Mortality	0.17	0.88	3.5
Bacillus anthracis	Mortality__per__100000	0.00019	0.0010	0.0040
Bacillus anthracis	Case__Fatality__Ratio	0.013	0.017	0.020
Bacillus anthracis	DALYs	5.3	31	130
Bacillus anthracis	DALYs__per__100000	0.0061	0.035	0.15
Bacillus anthracis	DALYs__per__Case	0.064	0.75	5.2
Clostridium botulinum toxin	Incidence	18	35	70
Clostridium botulinum toxin	Incidence__per__100000	0.020	0.040	0.080
Clostridium botulinum toxin	Mortality	0.78	2.7	7.9
Clostridium botulinum toxin	Mortality__per__100000	0.00089	0.0031	0.0090
Clostridium botulinum toxin	Case__Fatality__Ratio	0.10	0.30	0.50
Clostridium botulinum toxin	DALYs	16	87	360
Clostridium botulinum toxin	DALYs__per__100000	0.018	0.099	0.41
Clostridium botulinum toxin	DALYs__per__Case	0.53	2.4	8.7
Lathyrus sativus	Incidence	150	1,500	15,000
Lathyrus sativus	Incidence__per__100000	0.17	1.7	17
Lathyrus sativus	Mortality	0	0	0
Lathyrus sativus	Mortality__per__100000	0	0	0
Lathyrus sativus	Case__Fatality__Ratio	0	0	0
Lathyrus sativus	DALYs	140	6,700	96,000
Lathyrus sativus	DALYs__per__100000	0.16	7.6	110
Lathyrus sativus	DALYs__per__Case	0.24	3.3	25
Rift Valley Fever virus	Incidence	0.00031	0.0077	0.079
Rift Valley Fever virus	Incidence__per__100000	0.00000036	0.0000088	0.000091
Rift Valley Fever virus	Mortality	0.0000021	0.000063	0.00072
Rift Valley Fever virus	Mortality__per__100000	0.0000000024	0.000000072	0.00000082
Rift Valley Fever virus	Case__Fatality__Ratio	0.0030	0.010	0.020
Rift Valley Fever virus	DALYs	0.0000040	0.00030	0.0053
Rift Valley Fever virus	DALYs__per__100000	0.0000000045	0.00000034	0.0000060
Rift Valley Fever virus	DALYs__per__Case	0.00031	0.093	2.7
Rotavirus	Incidence	390,000	1,300,000	3,500,000
Rotavirus	Incidence__per__100000	450	1,400	4,100
Rotavirus	Mortality	170	640	2,100
Rotavirus	Mortality__per__100000	0.19	0.74	2.4
Rotavirus	Case__Fatality__Ratio	0.00047	0.00051	0.00055
Rotavirus	DALYs	14,000	55,000	180,000
Rotavirus	DALYs__per__100000	17	63	200
Rotavirus	DALYs__per__Case	0.0081	0.050	0.23
Staphylococcus aureus toxins	Incidence	44,000	68,000	100,000
Staphylococcus aureus toxins	Incidence__per__100000	51	77	120
Staphylococcus aureus toxins	Mortality	140	330	690
Staphylococcus aureus toxins	Mortality__per__100000	0.16	0.37	0.79
Staphylococcus aureus toxins	Case__Fatality__Ratio	0.0024	0.0050	0.0090
Staphylococcus aureus toxins	DALYs	37	100	260
Staphylococcus aureus toxins	DALYs__per__100000	0.042	0.12	0.30
Staphylococcus aureus toxins	DALYs__per__Case	0.00060	0.0015	0.0035
Taenia saginata	Incidence	55,000	740,000	5,500,000
Taenia saginata	Incidence__per__100000	62	850	6,300
Taenia saginata	Mortality	0	0	0
Taenia saginata	Mortality__per__100000	0	0	0
Taenia saginata	Case__Fatality__Ratio	0	0	0
Taenia saginata	DALYs	81	31,000	990,000
Taenia saginata	DALYs__per__100000	0.092	36	1,100
Taenia saginata	DALYs__per__Case	0.000086	0.077	3.1

	A	B	C	D	E	F
1						
2			***Mortality AFM1***			
3		**Inputs**				
4			*Incidence AFM1*	*CFR AFM1*		
5		lower	0.200	0.830	Check means	
6		mean	0.875	0.87	0.958	0.864
7		upper	3.62	0.90		
8						
9		**Calculations**				
10			*Incidence AFM1*	*CFR AFM1*		
11		lower log	-0.699	-0.081		
12		upper log	0.559	-0.046		
13		mean log	-0.070	-0.063		
14		sd log	0.321	0.009		
15		var log	0.103	0.000		
16						
17			*Mortality AFM1*			
18		mean log	-0.133			
19		var log	0.103			
20		sd log	0.321			
21		lower log	-0.763			
22		upper log	0.496			
23						
24		**Results**				
25			*Mortality AFM1*			
26		lower	0.173	Check		
27		mean	0.828	0.761		
28		upper	3.131			

Figure 1: Multiplication of two lognormal uncertainty distributions in Excel

4.2 Beta distributions

Figure 2 shows the calculation of quantiles of an uncertainty distribution for proportions, illustrated by the case-fatality ratio of liver cancer due to AFM1. It is assumed that this is the same as for aflatoxin B1 (AFB1) as the cancer caused by these two hazards is the same. According to FERG data, there were 433 cases of liver cancer due to AFB1 in Ethiopia in 2010, of which 376 died. These inputs are entered in cells C5:C6. We use a Bayesian approach to estimate the parameters and quantiles of a Beta distribution to model the uncertainty in the case-fatality ratio (*12*). A Beta distribution is bounded between 0 and 1 and can take many shapes, which makes it a good choice to model proportions. If there are n cases and s deaths, the Beta distribution defining the uncertainty around the mean is:

$$Beta(s+1, n-s+1) \tag{16}$$

The parameters of this distribution are calculated in cells D5:D6. The mean case-fatality ratio is $(s+1)/(n+2)$, which is calculated in cell C12. Quantiles of this distribution (i.e., the 2.5, 50 and 97.5 percentiles) can be calculated using the inverse Beta function in `Excel`, e.g., $upper = BETA.INV(0.975, C5, C6)$, and are provided in cells C10, C11 and C13.

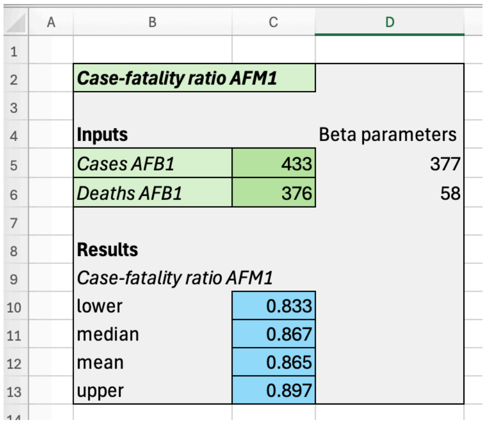

	A	B	C	D
1				
2		***Case-fatality ratio AFM1***		
3				
4		**Inputs**		Beta parameters
5		*Cases AFB1*	433	377
6		*Deaths AFB1*	376	58
7				
8		**Results**		
9		*Case-fatality ratio AFM1*		
10		lower	0.833	
11		median	0.867	
12		mean	0.865	
13		upper	0.897	

Figure 2: Estimation of quantiles of Beta uncertainty distribution for proportions

4.3 Gamma distributions

Figure 3 shows the calculation of quantiles of an uncertainty distribution for rates, illustrated by the incidence of anthrax due to infection with *Bacillus anthracis*. According to (*1*), there were 5,197 human cases of anthrax in Ethiopia in 5 years. These inputs are entered in cells C5:C6. We use a Bayesian approach to estimate the parameters and quantiles of a Gamma distribution to model the uncertainty in the incidence rate (*12*). A Gamma distribution is

bounded between 0 and ∞, and is often used to model rates. If there are n cases in y years, the Gamma distribution defining the uncertainty around the mean is:

$$Gamma(n, 1/y)$$

Here, n is the shape parameter and $1/y$ the scale parameter. The parameters of this distribution are calculated in cells D5:D6. The mean incidence is $n \times (1/y)$, which is calculated in cell C12. Quantiles of this distribution (i.e., the 2.5, 50 and 97.5 percentiles) can be calculated using the inverse Gamma function in `Excel`, e.g., $upper = GAMMA.INV(0.975, C5, C6)$, and are provided in cells C10, C11 and C13.

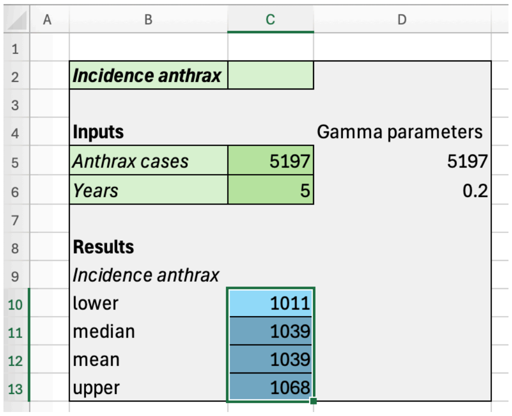

	A	B	C	D
1				
2		***Incidence anthrax***		
3				
4		**Inputs**		Gamma parameters
5		*Anthrax cases*	5197	5197
6		*Years*	5	0.2
7				
8		**Results**		
9		*Incidence anthrax*		
10		lower	1011	
11		median	1039	
12		mean	1039	
13		upper	1068	

Figure 3: Estimation of quantiles of Gamma uncertainty distribution for rates

5 Disease burden dashboard

The disease burden dashboard was created to provide users with a friendly, yet comprehensive way of visualizing, and comparing data on disease burden of FERG hazards. Features include multiple ways of graphing hazard data, multiple scaling options and fine-grained capability to compare a subset of hazards side by side. It has since expanded to generate and graph data for additional hazards through a user accessible simulation. This functionality has been used to add the burden of non-FER hazards selected by Ethiopina stakehoilders to the dashboard. In addition, users are able to run the simulation on data collected for custom hazards of chocie and feed the results back into the dashboard to be visualized alongside the other hazards.

The plots, graphical interface and simulations were created in R statistical software using the Shiny and ggplot2 packages. The dashboard can be accessed at https://osu-cfi.shinyapps.io/ethdashboard/.

The following sections describe the data, terminology and features of the dashboard.

5.1 Data

This section discusses how the data in the dashboard was collected and used, and relevant definitions.

5.1.1 Data Collection and Usage

The dashboard graphically plots two different data sets. The first data set, referred to as FERG Hazards in the following sections, contains Ethiopian estimates of foodborne disease burden attributed to various hazards obtained from FERG report. The second data set, referred to as non-FERG hazards, contains data on hazards that did not have FERG estimates available but were prioritized by the Ethiopian stakeholders. Monte Carlo samples for the dashboard were generated using the 'Minimum Quantile Information Distribution' in the `mc2d` package. This distribution uses linear interpolation between three defined quantiles to construct a cumulative distribution function (cdf) (*3*). The minimum and maximum of the cdf are defined by an overshoot k, i.e., the cdf is expanded on both sides by $k\%$ of the range between the lower and upper quantiles.

5.1.2 Risk Metric Definitions

Each hazard in both data sets listed above have multiple risk metrics that describe it. The dashboard allows users to select which metric to visualize. The definitions of each metric are listed below:

- Incidence – Number of new cases of disease during a specified time interval.
- Incidence_Rate_100K – Incidence rate per 100,000 people per year.
- Mortality - Number of new deaths that occur during during a specified time interval.
- Mortality_Rate_100K –The number of deaths per 100,000 people per year.
- Disability-Adjusted Life Year (DALY) - A health gap measure that combines the years of life lost due to premature death (YLL) and the years lived with disability (YLD) from a disease or condition, for varying degrees of severity, making time itself the common metric for death and disability. One DALY equates to 1 year of healthy life lost.
- DALY_Rate_100K - The number of DALYs per 100,000 peole per year.
- Case_Fatality_ratio - Proportion of people who die from a specified disease among all individuals diagnosed with the disease over a certain period of time.
- DALY_per_case - Number of DALYs divided by incidence.
- Years of Life Lost (YLL) – The number of deaths due to a specific disease or condition multiplied by the standard life expectancy at the age at which death occurs.

- Years Lived with Disability (YLD) – Number of years lived with a disability due to a specific disease or condition multiplied by a diasability weight.

5.2 Features

5.2.1 All Hazards Tab

The All Hazards Tab by default displays all of the FERG and Non-FERG hazards in a single set of box plots. As shown below in Figure 4, the red box plots denote FERG hazards and the blue box plots denote the Non-FERG hazards.

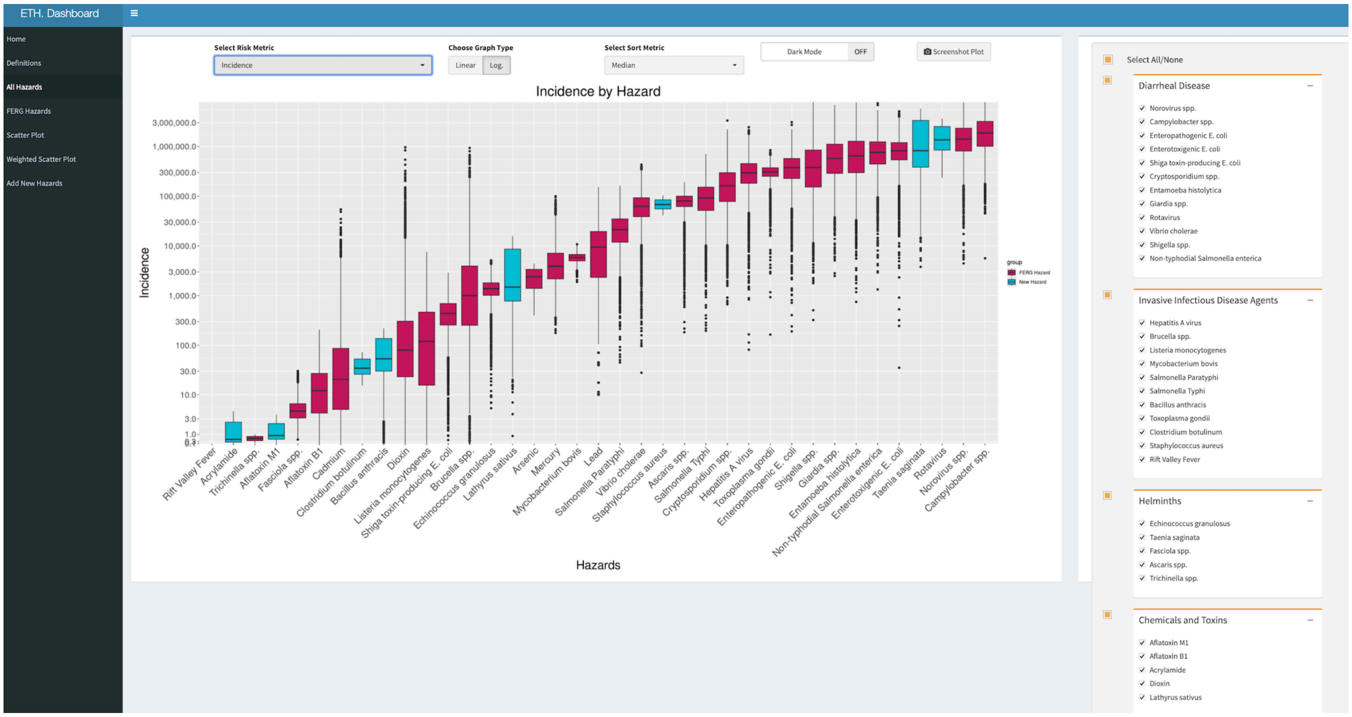

Figure 4: All Hazards tab

The top left drop-down box labeled "Select Risk Metric" allows users to choose from the following risk metrics to display on the y-axis defined in Section 5.1.2:

- Incidence
- Mortality
- DALYs
- Incidence_Rate_100K
- Mortality_Rate_100K
- DALY_Rate_100K

- Case_Fatality_ratio
- DALY_per_case

The "Choose Graph Type" select box switches the y-axis between a logarithmic and linear scale. The default scale is the log scale.

The "Select Sort Metric" allows users to sort the x-axis based on the alphabetical order of hazard names or the median risk metric value. The default sort metric is the median i.e. the hazards on the x-axis are ordered such that the hazard's median value is increasing.

Two additional boxes allow the user to select Dark Mode, and to create a screenshot of the plot for future reference.

Finally, the checkboxes on the right hand side allow users to select which hazards to plot. Two levels of granularity are given: users are able to select/deselect individual hazards one at a time or by entire hazard groups. Hazard groups include Diarrheal Disease Agents, Invasive Infectious Disease Agents, Helminths, Chemicals and Toxins and Metals.

Figure 5 below is an example where we only want to compare Helminths and Metals and choose specific hazards within these two groups.

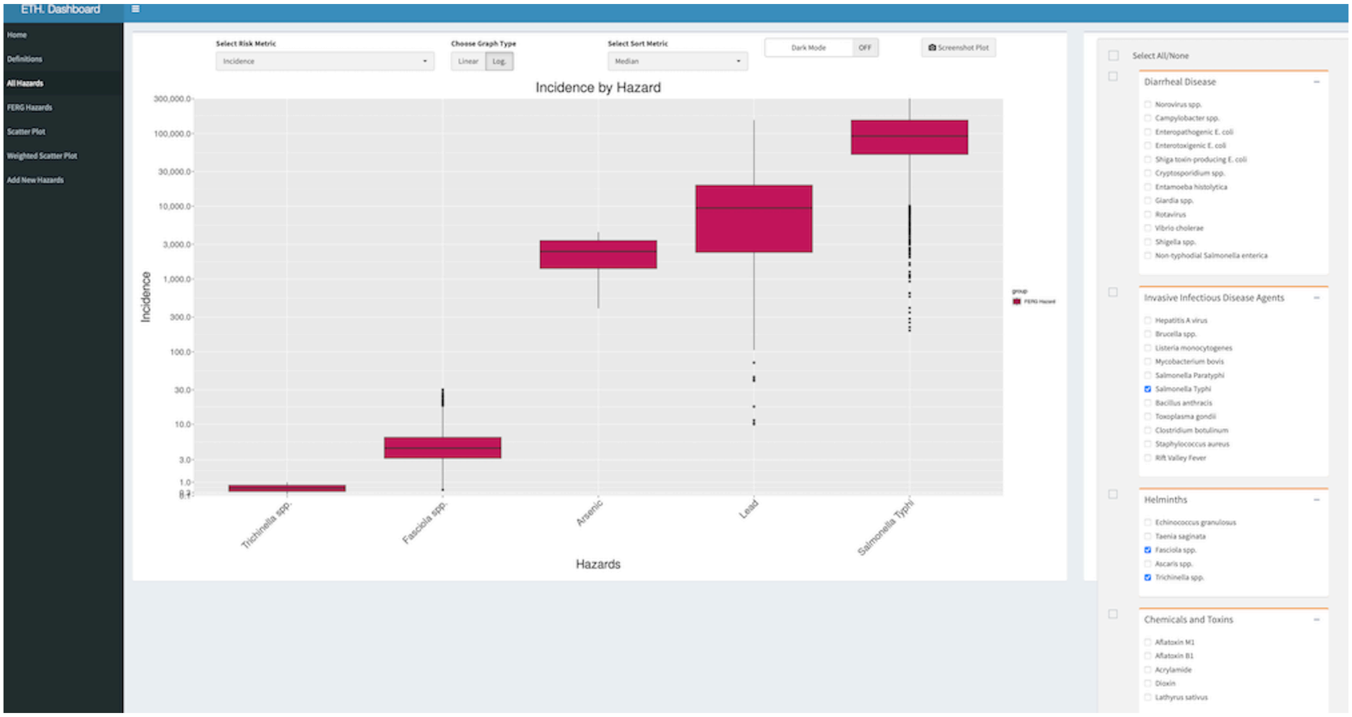

Figure 5: All Hazards tab selection

5.2.2 FERG Hazards

THe FERG Hazards tab displays the data for the FERG hazards only. The layout and functionality is the same as the All Hazards tab. FERG disease burden estimates are available for the total population as well as for two age groups (children under 5 years of age and people over 5 years of age). An additional drop-down box Select Dataset is provided to allow users to choose between the two datasets.

5.2.3 Scatter Plot

The Scatterplot tab allows users to create two-dimensional plots of the data by choosing different metrics for the x-axis and y-axis, see Figure 6. This allows users to explore, for example, the two dimensions of risk (e.g. incidence as a metric of likelihood and DALYs per case as a metric of severity) for each hazard.The scatterplot is labeled by hazard names, color coded by Hazard Group. The user can select the x-axis and y-axis metrics from the dropdown menus. The user can also select the metrics on both axes as well as the dataset using the Graph Options drop-down box.

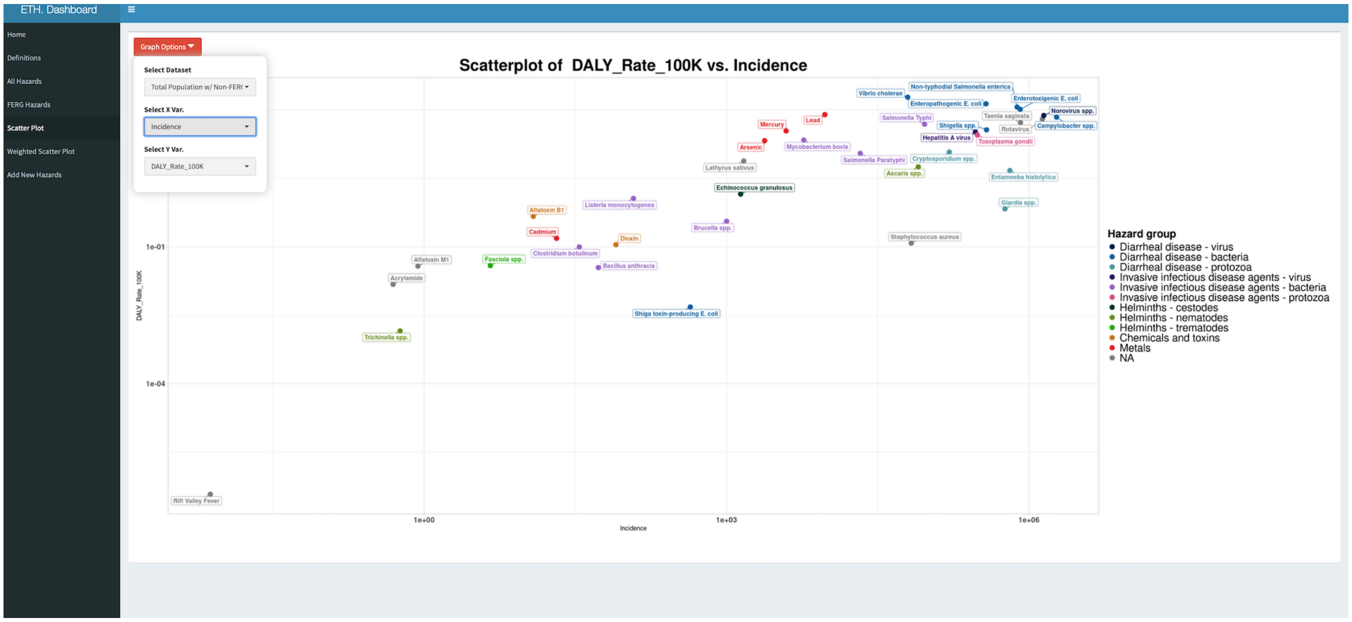

Figure 6: Scatter Plot tab

5.2.4 Weighted Scatter Plot

The Weighted Scatterplot (Figure 7) allows the user to add a third dimension to the plot, with the chosen metric for the third dimension being used to calculate the size of the dots. The

functionality is otherwise the same as for the Scatter Plot.

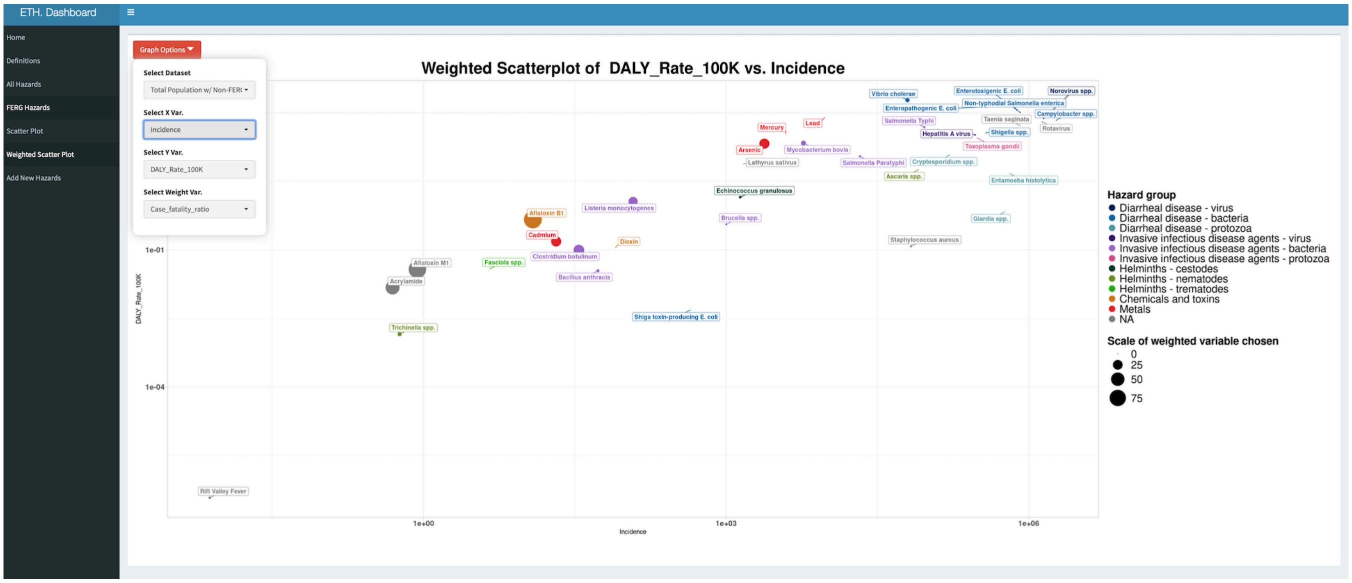

Figure 7: Weighted Scatter Plot tab

5.2.5 Add New Hazards

The Add New Hazards tab allows users to generate data for custom hazards. Detaile dinstructions are provided on the web page (Figure 8).

The simulation consists of three steps:

1. Gathering and Preparing the Data

   The simulation requires the lower,middle and upper distribution value of each hazard. These should be formatted as in Figure 9.

2. Formatting the Data

   Once all the distribution values have been calculated, the data must be formatted in an Excel or CSV file. The exact format can be found in the second select tab labelled "2. Formatting the Data".

3. Upload and Running the Simulation

   The final step is to upload the formatted Excel or CSV file and pressing the "Run and Download" button.

The resulting data from the simulation can then be plotted in the dashboard.

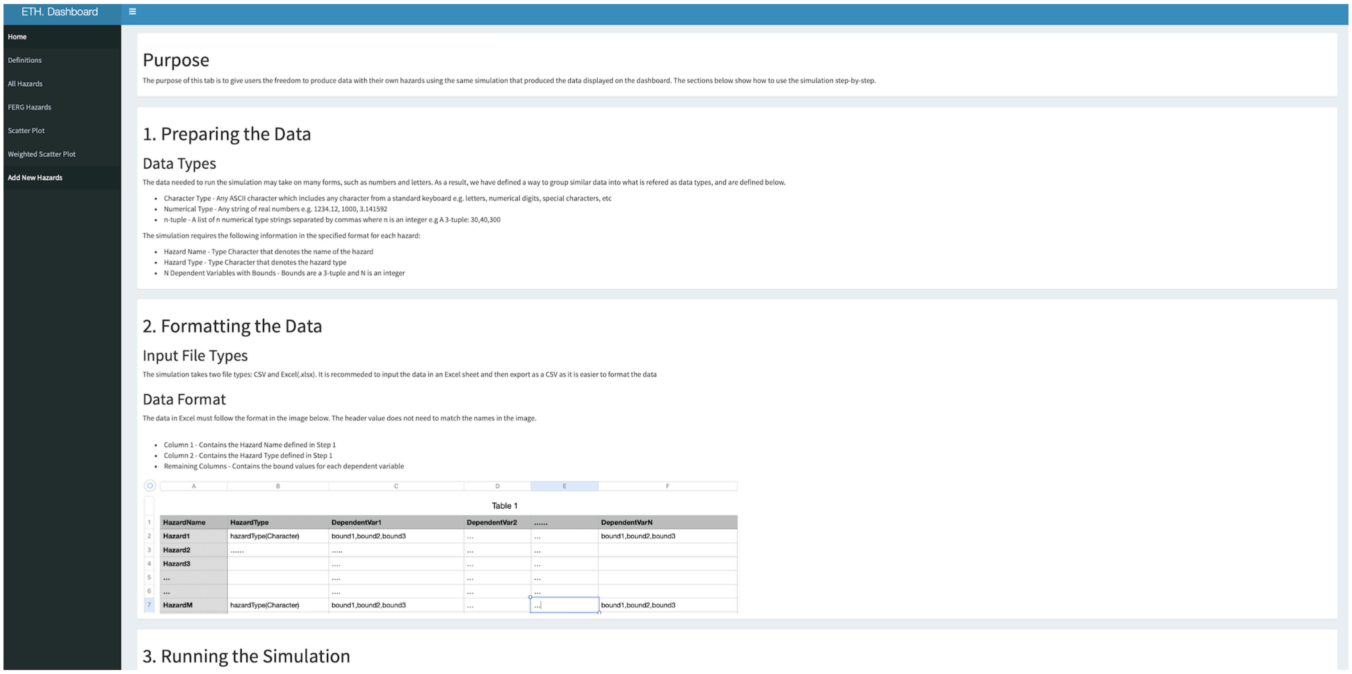

Figure 8: Add New Hazards tab

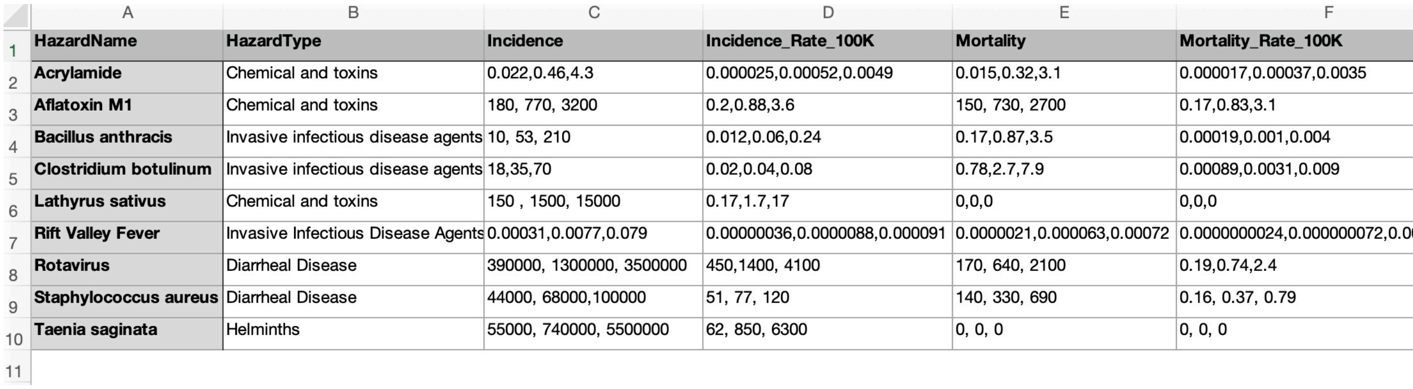

HazardName	HazardType	Incidence	Incidence_Rate_100K	Mortality	Mortality_Rate_100K
Acrylamide	Chemical and toxins	0.022,0.46,4.3	0.000025,0.00052,0.0049	0.015,0.32,3.1	0.000017,0.00037,0.0035
Aflatoxin M1	Chemical and toxins	180, 770, 3200	0.2,0.88,3.6	150, 730, 2700	0.17,0.83,3.1
Bacillus anthracis	Invasive infectious disease agents	10, 53, 210	0.012,0.06,0.24	0.17,0.87,3.5	0.00019,0.001,0.004
Clostridium botulinum	Invasive infectious disease agents	18,35,70	0.02,0.04,0.08	0.78,2.7,7.9	0.00089,0.0031,0.009
Lathyrus sativus	Chemical and toxins	150 , 1500, 15000	0.17,1.7,17	0,0,0	0,0,0
Rift Valley Fever	Invasive Infectious Disease Agents	0.00031,0.0077,0.079	0.00000036,0.0000088,0.000091	0.0000021,0.000063,0.00072	0.0000000024,0.000000072,0.0[illegible]
Rotavirus	Diarrheal Disease	390000, 1300000, 3500000	450,1400, 4100	170, 640, 2100	0.19,0.74,2.4
Staphylococcus aureus	Diarrheal Disease	44000, 68000,100000	51, 77, 120	140, 330, 690	0.16, 0.37, 0.79
Taenia saginata	Helminths	55000, 740000, 5500000	62, 850, 6300	0, 0, 0	0, 0, 0

Figure 9: Format to import data on custom hazards in dashboard

6 Risk ranking

6.1 Methods

Data from the first two rounds of the risk ranking workshop were collected in spreadsheets and merged into a single dataset. All data extraction, manipulation, plots and statistical testing were generated in **R** statistical software (*6*), using the `dplyr` package (*14*) for data processing. The final dataset is available in file `rr_dat.rds`.

Descriptive statistics of the risk ranking results included cross-tabulations and stacked bar-charts using the `ggplot2` package (*13*) while mosaic plots were prepared using the `ggmosaic` package (*5*). Univariate and multivariate ordinal logistic regression models were created using the `polr` function in the `MASS` package (*9*). The `GGally` (*8*) package was used to visually check for multicollinearity. Model selection was based on the Akaike Information Criterion. The proportional odds assumption was checked using the Brant test. The final model was used to identify variables that were most predictive of the rank in round 2. Information on the software versions used is provided in the Session Information at the end of this document.

6.2 Results

6.2.1 Descriptive analysis

The ranking results for each hazard in round 1 are shown in Figure 10. There were five hazards that were assigned the same rank by all groups (High: *Mycobacterium bovis*, Medium: Shiga-toxin producing *Escherichia coli* and *Trichinella* spp., Low: *Echinococcus granulosus* and Rift Valley Fever virus) and these ranks were considered final.

In round 2, groups ranked 32 hazards and five ranks were carried over from round 1. Overall changes in ranking are visualized in Figure 11. There was a high number of hazards that were ranked Low in both rounds but changes from Low to Medium did occur. Changes from Low to High did not occur. Most hazards that were ranked Medium in round 1 were also ranked Medium in round 2, but changes occurred to both Low and High ranks. Most changes in ranking occurred for hazards that were ranked High in round 1, changing to Medium or even Low ranks.

A more detailed analysis of changes in ranking from round 1 to round 2 per metric used in round 1 is presented in Figure 12. The group that used incidence as the metric in round 1 changed 20 out of 33 rankings. Hazards with High or Medium rank were reassigned to the same categories but with relatively many crossovers and some hazards were moved from Low to Medium rank. The group using mortality as metric in round 1 changed 27 out of 33 rankings, mainly Medium and Low ranks in round 1. The group using case-fatality ratio as metric in round 1 changed 22 out of 33 ranking, mainly crossovers between High and Medium. The

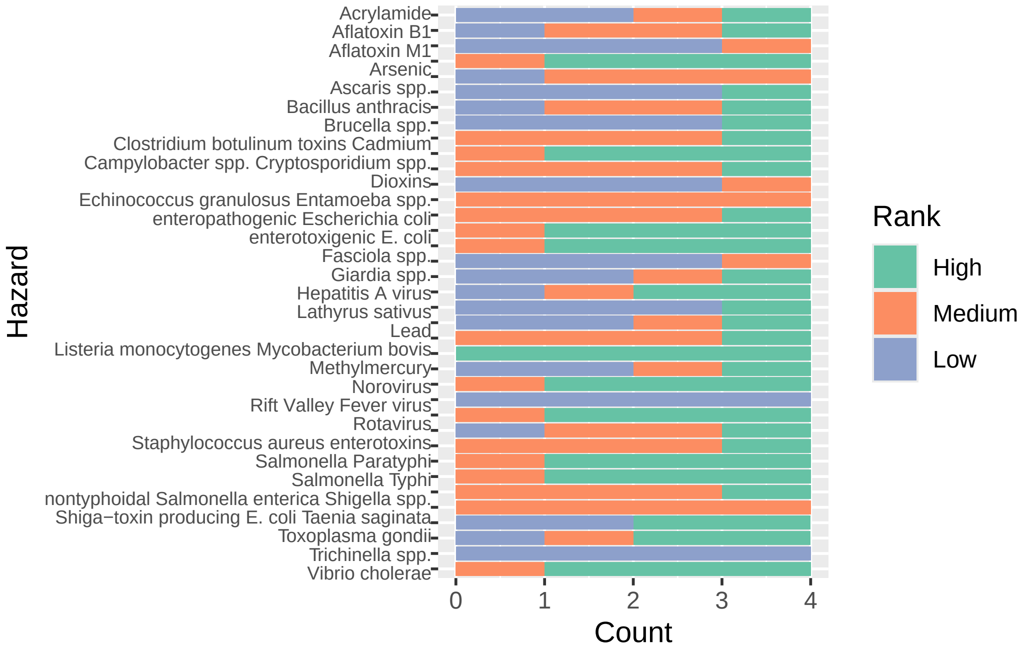

Figure 10: Risk ranking results in round 1

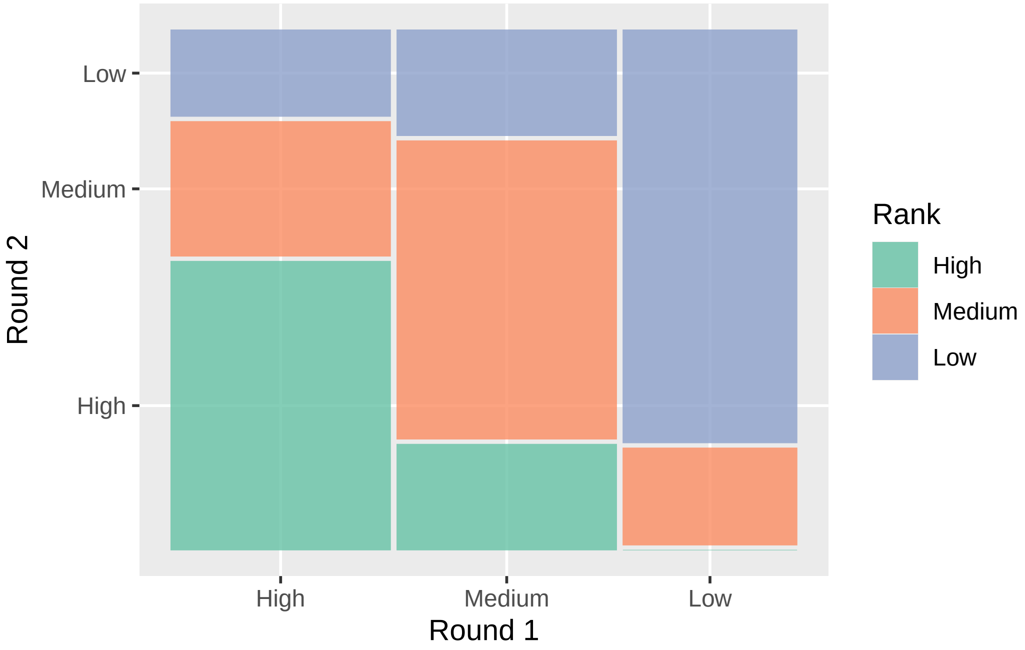

Figure 11: Changes in ranking from round 1 to round 2

group using DALYs as metric in round 1 changed 26 out of 33 rankings, mainly downranking hazards ranked as High or Medium in round 1.

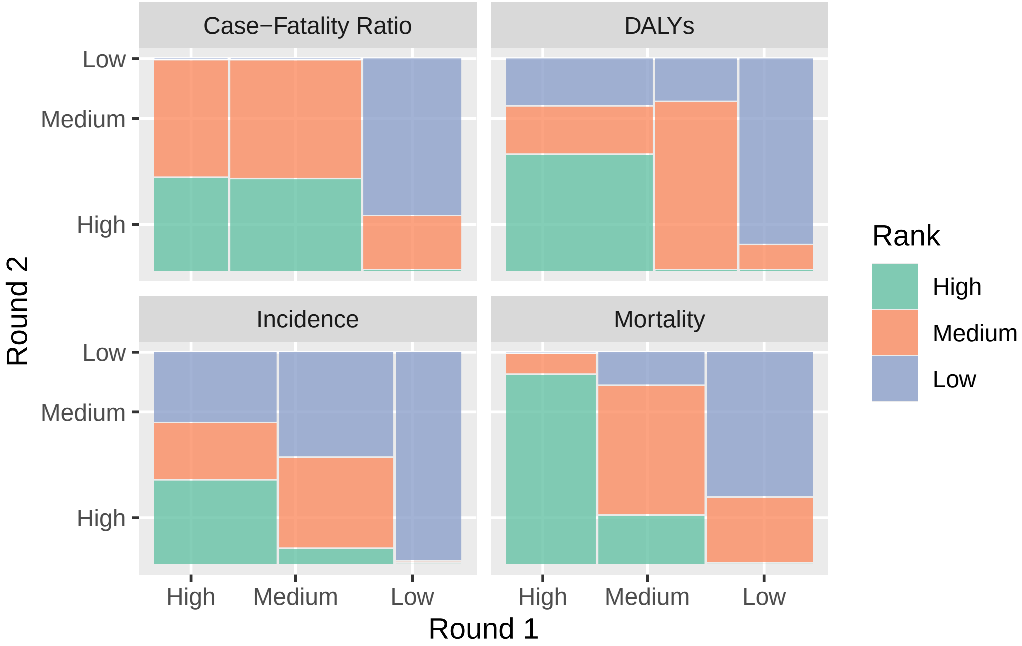

Figure 12: Changes in ranking from round 1 to round 2 by metric assigned to groups in round 1

The ranking results for each hazard following round 2 are shown in Figure 13. There were fifteen hazards that were assigned the same rank by all groups (High: enterotoxigenic *Escherichia coli*, *Mycobacterium bovis*,rotavirus, *Salmonella enterica* subsp. *enterica* (non-typhoidal) and *Vibrio cholerae*; Medium: *Cryptosporidium* spp., *Echinococcus granulosus*, *Salmonella enterica* subsp. *enterica* serovar Paratyphi and Shiga-toxin producing *Escherichia coli*; Low: dioxins, *Fasciola* spp., *Lathyrus sativus*, Rift Valley Fever virus, *Taenia saginata* and *Trichinella* spp.) and these ranks were considered final.

Ranking of hazards for which no agreement was reached after round 2 were finalized by group discussions as described in the main text.

6.2.2 Ordinal logistic regression

In the univariate analysis, all predictor variables were highly significant (Table 3). The multivariate model was developed using backward selection, starting with the model including all significant variables in the univariate analysis. There was substantial correlation between the disease burden metrics, see Figure 14.

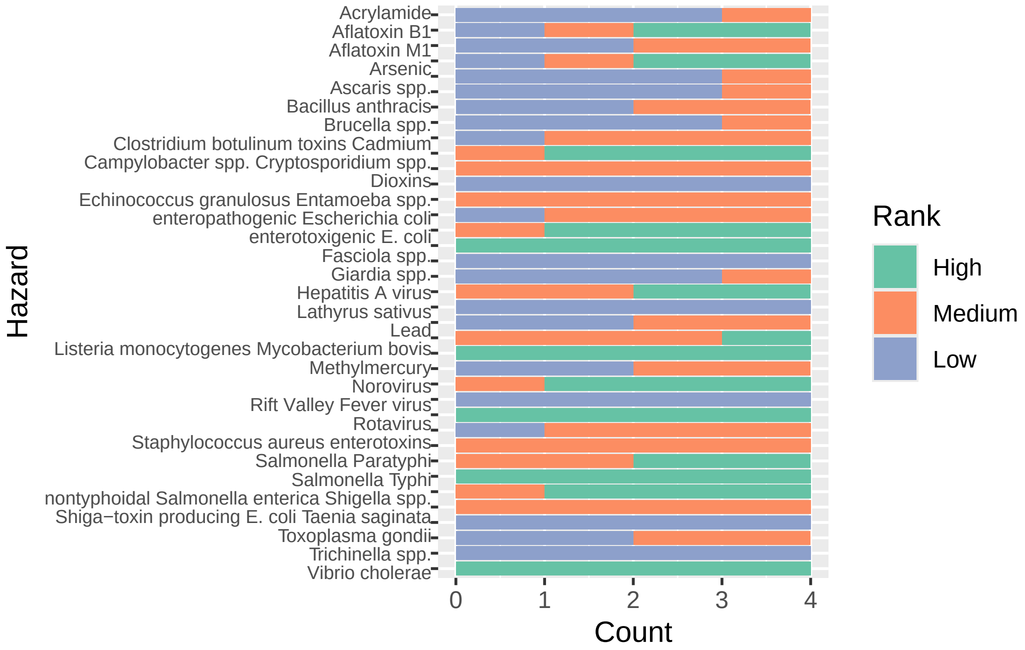

Figure 13: Risk ranking results in round 2

Table 3: Univariate analysis for round 2 rank

	Odds ratio		
Variable	Median	2.5%	97.5%
Rank1 Medium	3.27	1.55	7.07
Rank1 Low	42.10	15.63	125.19
Incidence rate(log10)	0.61	0.51	0.73
Mortality rate(log10)	0.57	0.49	0.64
Case-fatality ratio (log10)	0.80	0.71	0.90
Disability-Adjusted Life Years rate (log10)	0.66	0.58	0.74

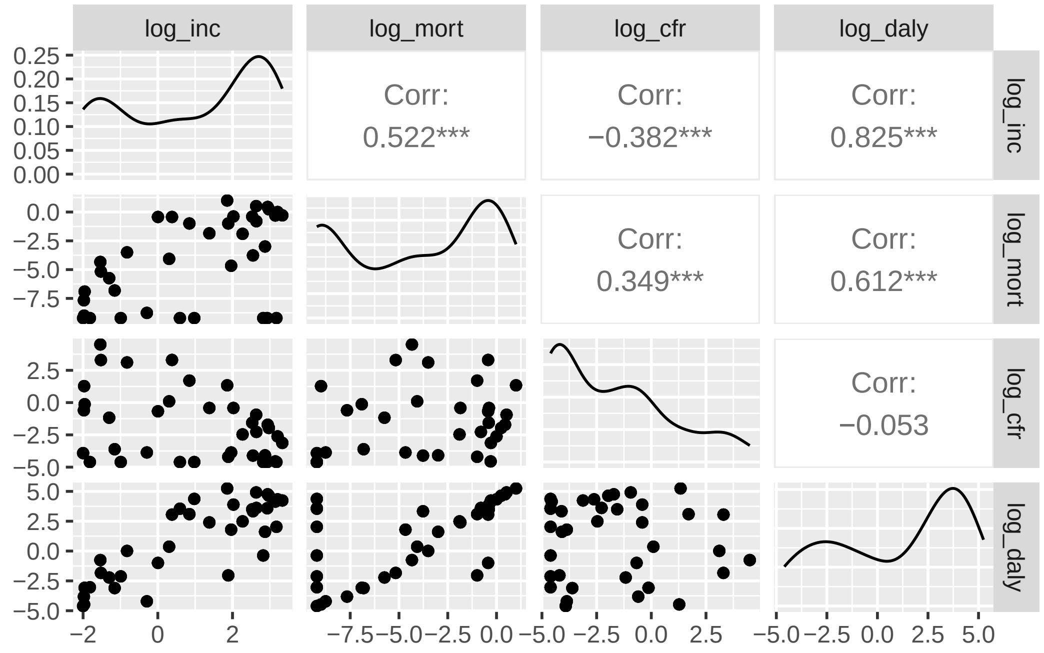

Figure 14: Correlation between disease burden metrics

The final model included the rank in round 1 and log10 mortality as the strongest predictors and an interaction term between these variables (Table 4). If Rank1 was Low, the odds of Rank2 being Low (vs. Medium or High) was 105 times higher than if Rank1 was Medium or High. For every unit increase in log mortality, the odds of Rank2 being Low (vs. Medium or High) decreased by 52%.

The interaction between the two predictors is shown in Figure 15. The top row of the figure suggests that the probability of Rank2 being Low increases if Rank1 moves from High to Low and decreases with increasing log mortality. The probability of Rank2 being High decreases if Rank1 moves from High to Low and increases with increasing log mortality (bottom row of

Table 4: Multivariate analysis for round 2 rank

	Odds ratio		
Variable	Median	2.5%	97.5%
rank1Medium	4.913263	1.5910469	16.0600742
rank1Low	105.352138	10.6810331	1245.9507300
log_mort	0.478776	0.3606483	0.6020927
rank1Medium:log_mort	1.399504	1.0639050	1.8998095
rank1Low:log_mort	1.677238	1.1682069	2.4689701

the figure). The probability of Rank2 being Medium is independent of Rank1 and there is no monotonous trend with log mortality. Note that the effect of log mortality on Rank2 is strong. For example, the upper right pane in the plot shows that the probability of Rank 2 being Low if Rank 1 is Low, decreases from approximately 60% to almost 0% if log mortality increases from -8 to 0. Likewise, if Rank1 is High, the probability of Rank2 being High increases from approx. 0% to approx. 80% if log mortality increases from -8 to 0.

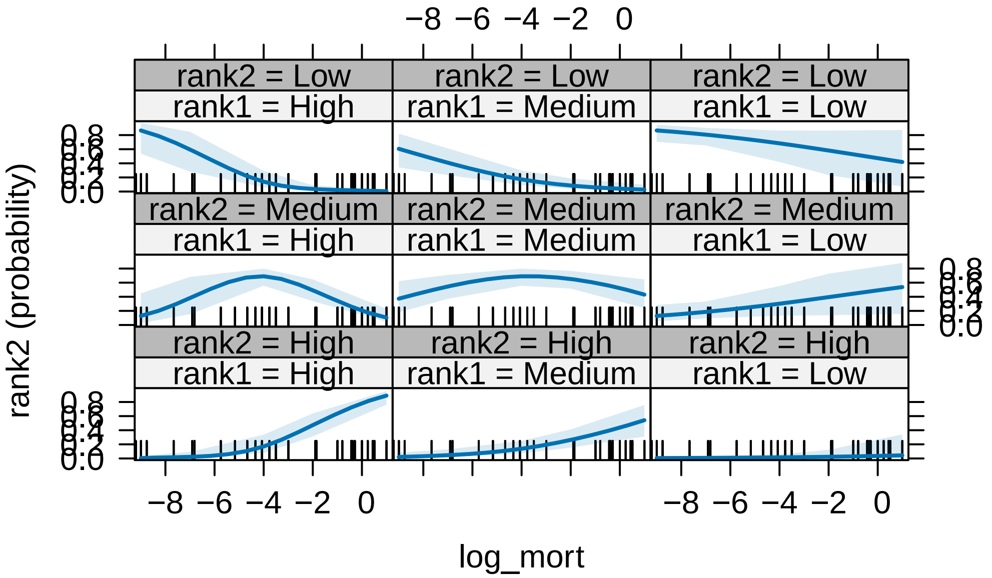

Figure 15: Interaction between predictors of rank in round 2

7 Attribution of foodborne deaths to hazards

7.1 The Delphi method

We used a Delphi process to collect information from Ethiopian experts on attribution of foodborne deaths to food groups. The Delphi method is a structured, interactive technique to elicit information from a panel of experts and aims to move towards consensus about the study objective. To this purpose, experts answered questions in two rounds.

- In Round 1, experts individually provided estimates of the proportion of illness from a given hazard, attributed to different food groups after having been briefed in a webinar about the specific goals of the study and how to complete the elicitation instrument.
- After the first round, the experts received a summary of all the expert estimates and the rationale that was provided to support these estimates.

- In Round 2, the experts were provided the opportunity to revise their individual estimates in the light of the group results.
- Results from Round 2 were summarized and used to present data on the impact of foodborne disease by hazard and food group to the risk prioritization workshop.

7.2 Round 1

7.2.1 Elicitation instrument

Experts were provided a spreadsheet to complete their attribution estimates, see Figure 16. In addition to the sheet shown here, there was also a sheet with free text fields for each hazard, in which the experts could provide the rationale for their estimates.

Hazard	Beef	Small Ruminant Meat	Dairy	Poultry Meat	Eggs	Vegetables	Fruits and Nuts	Grains and Beans	Oils and Sugar	Fish and Shellfish	Other	Specify other	Total
Aflatoxin B1					0								0
Arsenic					0								0
Campylobacter spp.					0								0
Enteropathogenic *Escherichia coli*					0								0
Enterotoxigenic *Escherichia coli*					0								0
Mycobacterium bovis	0	0	100	0	0	0	0	0	0	0	0		100
Non-typhoidal *Salmonella enterica*													0
Norovirus					0								0
Rotavirus					0								0
Salmonella Typhi					0								0
Shigella spp.					0								0
Vibrio cholerae					0								0

Figure 16: Elicitation instrument

The instructions to complete the elicitation instrument were:

- The aim of this exercise is to attribute all cases of *foodborne disease* by 12 *hazards* that were assigned a high priority in the risk ranking workshop in a *typical year* in Ethiopia to *food groups*.
- *Foodborne disease* is defined as a case of illness that was caused by exposure to a microbial or chemical hazard in food. Many of these hazards can also be transmitted by other pathways such as water, soil or contact with humans or animals. The data on foodborne illness that will be used in the study have already considered this attribution to major pathways.
- The *point of attribution* will be the point where the hazards entered the place where the foods are prepared for final consumption. Hence, experts are asked to consider both the risk of direct consumption of the food group as well as the risk of cross-contamination from the specified food group to the home kitchen environment or food preparation area and ready-to-eat foods prepared there. For example, attribution of *Campylobacter* to the food group "Poultry meat" includes the risks of eating (undercooked) poultry meat as

well as the risks of salads and other ready-to-eat foods that may have been contaminated through cutting boards, benchtops, knives, hands etc.

- A *typical year* is defined as a year in which no major incidents (e.g., a large outbreak but also COVID-19) affected the incidence of foodborne disease.
- *Food groups* included in the study are based on the WHO study, using 13 groups: beef, small ruminant's meat, dairy, pig's meat, poultry meat, eggs, vegetables, fruit and nuts, grains and beans, oils and sugars, finfish, shellfish, seaweed. Pork consumption is very low in Ethiopia and has been excluded. Eggs are not likely to be a relevant transmission pathway of any of the hazards except non-typhoidal *Salmonella enterica.* Consumption of fish, shellfish and seaweed is low to absent in Ethiopia and are excluded. Attribution to oils and sugars in Africa is very low and this group has also been excluded. A category "other foods" is included to allow experts to name any food groups they may consider relevant, including those removed by the study team.
- Consider all cases in the country, regardless of age, residence etc.
- Start by thinking about a hazard that you are familiar with. Ask yourself, how are foodborne cases by that hazard distributed across food groups in a typical year.
- For food groups of which you think not at all involved in the transmission of the hazard, enter 0 in the cell for the combination of that hazard and food group. For example, *Mycobacterium bovis* can only be transmitted by dairy. We have already assigned 0 to all food groups except dairy in the spreadsheet. We have also assigned 0 to eggs for transmission of all hazards except *Salmonella.* Then, consider food groups that you think are involved in transmission of a hazard and rank them from high to low. Distribute 100 percentage points over these food groups, according to your belief how important each food group is. If you believe a hazard is transmitted by food groups that are not included in the table, you can assign points to the group "Other". In that case, please specify the food in the next column.
- The sum of all points that you assign should be 100. You can check this in the column "Total", the cells will have a green color when the sum is 100.
- In this study, we only seek your best estimate and do not consider the uncertainty of these estimates.
- Once you have finalized your estimates for a specific hazard, briefly describe your rationale in the sheet "Rationale". Write your rationale in the preassigned cell in the sheet only; the text can be longer than the width of the cell provided.
- Repeat this process or all other hazards
- You may decide that you are not sufficiently familiar with one or more hazards to provide estimates. In that case, leave all cells for that hazard blank.

- Save the spreadsheet with the file name Exp*xx*.xlsx, where *xx* is the expert number that was communicated to you in the invitation email. This number is only known to one person in the Ohio State University Global One Health Initiative who is not involved in the study and serves to protect your anonymity.

7.2.2 Data

Completed spreadsheets were received from 15 experts. Table 5 provides details on adjustments made to expert sheets in Round1. These edits were necessary to assure consistency between the individual expert estimates and to assure the data would fit in the computational framework.

1. A draft expert sheet was distributed with the invitation for the webinar on May 25, 2023 providing details of the process. This draft was revised based on feedback received from the experts. The final expert sheet was distributed after this webinar. Several experts used the draft format. Their sheets were reformatted to be consistent with the final format by sorting the hazards alphabetically, adding the food groups "Oils and Sugar" and "Fish and Shellfish", and adding the hazard "Rotavirus".
2. Several experts only filled cells in their spreadsheet for food groups to which transmission of a hazard was attributed. For computational purposes, empty cells for these hazards were filled with 0's.
3. The column "Details" summarized edits that were unique to individual expert sheets.

7.2.3 Average expert estimates

The average of expert attribution estimates is presented in Table 6. For each hazard, the table presents the percentage of cases of illness that is attributed to each of the 11 food groups (including a group "Other"). The attribution percentages per hazard sum to 100%.

7.3 Expert agreement

Table 7 shows a metric for the (dis)agreement between experts, i.e., the standard deviation of the estimates (in percent) for each food-hazard pair. A zero in the table means that there was full agreement among the experts, the higher the value, the more disagreement. The final column in Table 7 shows the average standard deviation across each row, i.e., for each hazard. We note the the experts agreed most on ETEC and least on Rotavirus. For food groups, experts agreed most on OS and least on Vg. Note that *Mycobacterium bovis* was excluded from these considerations because it was assigned 100% to dairy by the study team.

Table 5: Adjustments to expert sheets

Expert	Format	Zeros	Details
2		Added	Redistributed aflatoxin B1 over non-animal source foods
4	R	Added	Expert added “Seafood”, which was available in new format;
NA			estimates moved to this column.
NA			Redistributed aflatoxin B1 over non-animal source foods
5		Added	S.Typhi Total was 110. Reduced all numbers by 10%.
NA			Redistributed aflatoxin B1 over non-animal source foods
9	R	Added	Redistributed “Other”
11			Redistributed aflatoxin B1 over non-animal source foods
12		Added	None
15		Added	Divided all entries by 4 or 5 (Salmonella) to assure “Total” is 100
16		Added	Redistributed “Other”
18		Added	Redistributed aflatoxin B1 over non-animal source foods
19		Added	Changed column heading to “Fish and Shellfish”
20			Redistributed “Other”
22	R	Added	M. bovis was attributed 100% to dairy by default.
NA			Redistributed aflatoxin B1 over non-animal source foods
24		Added	Removed three rows with hazards added by expert as not prioritized .
NA			Attributed Shigella and V. cholerae from eggsk to 0.
25			Redistributed “Other”
26	R		Moved “Fish”estimates moved to this column.
NA			Changed “Vegetables” for EPEC to 10 to assure “Total” is 100.
NA			Redistributed aflatoxin B1 over non-animal source foods

Table 6: Average of expert attribution estimates in Round 1 (%). See full food group names in Figure 16.

Hazard	Bf	SR	Dy	Py	Eg	Vg	FN	GB	OS	SF	Ot	Tl
Aflatoxin B1	0	0	0	0	0	5	19	66	2	3	6	100
Arsenic	5	3	4	3	0	39	14	12	0	19	1	100
Campylobacter	14	9	24	40	3	2	2	0	0	5	1	100
EPEC	22	15	25	12	1	15	6	1	0	3	1	100
ETEC	24	15	26	12	1	13	4	0	0	3	1	100
M. bovis	0	0	100	0	0	0	0	0	0	0	0	100
Norovirus	13	7	14	12	2	23	18	1	0	8	2	100
Rotavirus	30	10	5	5	0	11	2	0	0	22	14	100
S. Typhi	19	15	15	21	4	13	3	0	0	3	6	100
Salmonella	10	11	10	13	18	24	9	0	0	5	0	100
Shigella	9	10	11	12	0	28	15	4	1	7	2	100
V. cholerae	9	6	9	6	1	37	11	2	2	14	3	100

Table 7: Standard deviation of expert attribution estimates in Round 1 (%)

Hazard	Bf	SR	Dy	Py	Eg	Vg	FN	GB	OS	SF	Ot	Average
Aflatoxin B1	0	0	0	0	0	9	17	27	4	9	10	7
Arsenic	9	7	8	6	0	23	17	16	0	24	3	10
Campylobacter	10	8	16	24	8	3	3	1	0	10	4	8
EPEC	10	12	9	9	3	14	7	3	0	6	3	7
ETEC	7	11	14	9	3	11	5	1	0	6	2	6
M. bovis	0	0	0	0	0	0	0	0	0	0	0	0
Norovirus	11	10	11	13	4	21	19	2	0	11	4	10
Rotavirus	48	12	10	10	0	13	5	0	0	21	16	12
S. Typhi	9	11	10	12	8	11	4	1	1	5	11	8
Salmonella	10	14	10	11	26	24	13	1	1	13	1	11
Shigella	8	12	8	17	0	19	15	6	3	10	5	9
V. cholerae	12	10	9	9	3	31	9	4	4	27	8	11
NA	11	9	9	10	5	15	9	5	1	12	6	8

7.4 Expert rationale

Experts were asked to provide the rationale for their estimates, and these are reproduced *ad verbatim* in @-tbl-rat (Appendix A). This information was shared to help other experts evaluate the group consensus and decide whether they wanted to adjust their estimates in Round 2.

7.5 Evaluation Round 1

After considering the expert's estimates and rationale, the TARTARE team provided the following observations for consideration by the experts in round 2.

1. Aflatoxin B1 only occurs in foods of plant origin, mainly tree nuts and grains and oil seeds. It can also be present in animal feed. However, after ingestion by animals, aflatoxin B1 is converted into aflatoxin M1 and excreted in urine and milk. Aflatoxin B1 does not occur in meat and dairy. Aflatoxin M1 was included in the risk ranking workshop and was not considered a high priority foodborne hazard.

2. Arsenic occurs naturally in soils and can be accumulated by plants grown in contaminated soils. Imported rice is an important, but under-recognized source of exposure to arsenic in Africa. Fish mainly contains arsenobetain (the least toxic form of arsenic), rice, which contains primarily inorganic arsenic, which is much more toxic (L. van Ingenbleek, WHO, personal communication).

3. Enteropathogenic and enterotoxigenic *E. coli*, Norovirus, Rotavirus, *Salmonella* Typhi and *Shigella* spp. have exclusively human reservoirs. The reservoirs of *Vibrio cholerae* are humans and water. Cross-contamination from food handlers to animal source foods is possible, and Ethiopian experts have indicated that meat and dairy may be involved in the transmission of these hazards (*7*). However, it is unlikely that animal source foods are their main transmission routes.

4. Some experts mentioned the importance of water as a transmission route. There is no doubt that waterborne transmission contributes significantly to the spread of many hazards considered in this study. However, the differentiation between food- and waterborne exposure is already accounted for in the WHO FERG estimates and the attribution estimated from this study will be applied to estimates of the foodborne disease burden only. Experts should therefore not consider waterborne transmission in their estimates.

Table 8: Average of expert attribution estimates in Round 2(%)

Hazard	Bf	SR	Dy	Py	Eg	Vg	FN	GB	OS	SF	Ot	Tl
Aflatoxin B1	0	0	0	0	0	6	20	67	1	2	5	100
Arsenic	4	2	3	2	0	39	14	15	0	20	1	100
Campylobacter	14	9	23	40	3	3	2	0	0	5	1	100
EPEC	22	15	25	12	1	15	6	1	0	3	2	100
ETEC	24	15	26	12	1	13	4	0	0	3	1	100
M. bovis	0	0	100	0	0	0	0	0	0	0	0	100
Norovirus	14	8	16	13	2	22	16	1	0	7	2	100
Rotavirus	27	9	6	4	0	15	6	0	0	22	11	100
S. Typhi	19	15	15	21	4	13	3	0	0	3	6	100
Salmonella	10	11	12	12	17	23	9	0	0	5	1	100
Shigella	9	10	11	13	0	28	15	3	1	7	2	100
V. cholerae	10	6	10	6	1	37	10	2	2	14	3	100

7.6 Round 2

7.6.1 Instructions

All experts were invited to review the changes made to their worksheets by the study team and adjust their Round 2 estimates if they disagreed with any of the edits. Experts who used the draft format did not consider attribution of “Rotavirus” nor the newly added food groups and were invited to reconsider their estimates in Round 2 to take these changes into account.

Experts were also invited to review the Round 1 group results and the comments from the study team and, if they wished, change their estimates based on this information.

If they did not want to make any changes, they were asked to confirm by reply email. After a set deadline, the study team assumed Round 1 estimates were still valid.

7.6.2 Data

In Round 2, three experts provided revised estimates. For all other experts, the Round 1 estimates were considered final.

7.6.3 Updated expert estimates

Updated expert estimates are provided in Table 8. Because of the low number of revised estimates, the results are quite similar to those in Round 1.

Table 9: Prior attribution estimates (percent)

Hazard	Bf	SR	Dy	Py	Eg	Vg	FN	GB	OS	SF	Ot	Tl
Aflatoxin B1	0	0	0	0	0	0	50	50	0	0	0	100
Arsenic	0	0	0	0	0	0	0	90	0	10	0	100
Campylobacter	12	12	15	51	0	8	2	0	0	0	0	100
EPEC	13	9	24	21	0	19	7	3	3	1	0	100
ETEC	13	9	24	21	0	19	7	3	3	1	0	100
M. bovis	0	0	100	0	0	0	0	0	0	0	0	100
Norovirus	8	8	7	35	23	7	6	2	1	2	2	101
Rotavirus	13	9	24	21	0	19	7	3	3	1	0	100
S. Typhi	13	9	24	21	0	19	7	3	3	1	0	100
Salmonella	13	9	24	21	0	19	7	3	3	1	0	100
Shigella	13	9	24	21	0	19	7	3	3	1	0	100
V. cholerae	13	9	24	21	0	19	7	3	3	1	0	100

7.7 Attribution to food groups

In the risk ranking workshop, the experts indicated that mortality was the main metric considered in hazard ranking. This was also evident from the analysis presented in Table 4. Therefore, data on deaths per hazard/ food group were presented as the key input in the risk prioritization workshop. Note that attribution of foodborne deaths was assumed to be proportional to attribution of foodborne disease cases. In total, 1.48077×10^5 deaths were estimated to have occurred in 2010 due to foodborne disease in Ethiopia. The proportion of cases attributed by FERG to different food/hazard combinations for the AFRE subregion, to which Ethiopia belongs, is shown in Table 9. These estimates are the average for the subregion, and were updated for Ethiopia using the results from the Delphi survey.

FERG has not presented attribution estimates to food groups for pathogens with human reservoirs (ETEC, EPEC, *Shigella* spp., Norovirus) and arsenic. Estimates for ETEC were available for Ethiopia from an expert elicitation for the TARTARE and Pull Push projects (*7*). It was assumed that attribution for other pathogens with human reservoirs was the same as for ETEC. In Africa, fish and rice (mainly imported rice) are the main sources of foodborne exposure to arsenic (Luc van Inglenbeek, WHO; personal communication). A less toxic form of arsenic occurs in fish than in rice. Hence 90% of all deaths by arsenic were attributed to the food group "Grains_Beans" and 10% to "(Shell)fish".

We updated the prior attribution data using the data provided by the experts by calculating a weighted average. Let a_{ij} be the prior estimates of percentage points of deaths by hazard i assigned to food group j, and b_{ijk} be the percentage points of deaths by hazard i assigned to food group j by expert k. An equal weights average was chosen to combine the prior estmates and expert's inputs:

Table 10: Posterior attribution combining estimates from experts in Ethiopia and from FERG

Hazard	Bf	SR	Dy	Py	Eg	Vg	FN	GB	OS	SF	Ot	Tl
Aflatoxin B1	0	0	0	0	0	3	35	58	0	1	2	100
Arsenic	2	1	2	1	0	19	7	52	0	15	0	100
Campylobacter	13	11	19	46	2	5	2	0	0	3	1	100
EPEC	17	12	24	16	0	17	6	2	2	2	1	100
ETEC	19	12	25	17	0	16	6	2	2	2	1	100
M. bovis	0	0	100	0	0	0	0	0	0	0	0	100
Norovirus	11	8	11	24	13	14	11	1	0	5	2	100
Rotavirus	20	9	15	13	0	17	6	2	2	12	5	100
S. Typhi	16	12	19	21	2	16	5	2	2	2	3	100
Salmonella	12	10	18	16	8	21	8	2	2	3	0	100
Shigella	11	10	17	17	0	24	11	3	2	4	1	100
V. cholerae	11	8	17	14	0	28	9	2	2	7	1	100

$$\mu_{ij} = \frac{a_{ij} + \sum_k (b_{ijk}/k)}{2}$$

7.8 Updated attributable deaths

Applying the updated attribution estimates resulted in estimates of the number of foodborne deaths attributed to each hazard/food group pair, see Table 11.

The code below creates a data frame and two plots showing the distribution of attributable deaths by hazard and food group. Plots are included in the main text of the manuscript.

8 Prioritization

8.1 Foodborne deaths attributable to Supply Chain Control Points in four food value chains

The code in this section aggregates the attributable deaths to three categories of hazards and creates the number of deaths for each combination of hazards and food groups.

Table 11: Attributable deaths by hazard and food group, combining attribution estimates from experts in Ethiopia and literature

Hazard	Bf	SR	Dy	Py	Eg	Vg	FN	GB	OS	SF	Ot	Total
Aflatoxin B1	0	0	0	0	0	2	20	32	0	1	1	56
Arsenic	13	6	13	6	0	123	45	337	0	97	0	640
Campylobacter	86	73	125	304	13	33	13	0	0	20	7	674
EPEC	248	175	350	233	0	248	87	29	29	29	15	1443
ETEC	210	133	277	188	0	177	66	22	22	22	11	1128
M. bovis	0	0	125	0	0	0	0	0	0	0	0	125
Norovirus	148	108	148	324	175	189	148	13	0	67	27	1347
Rotavirus	173	78	130	113	0	147	52	17	17	104	43	874
S. Typhi	102	77	122	134	13	102	32	13	13	13	19	640
Salmonella	71	60	107	95	48	125	48	12	12	18	0	596
Shigella	44	40	67	67	0	95	44	12	8	16	4	397
V. cholerae	260	189	402	331	0	663	213	47	47	166	24	2342
Total	1355	939	1866	1795	249	1904	768	534	148	553	151	10262

Table 12: Number of foodborne deaths per year in Ethiopia by three groups of hazards attributed to four food chains

	**CATEGORIES**			
**Foodgroups**	**AnthroponoticPathogens**	**ZoonoticPathogens**	**Chemicals**	**Total**
Red Meat	1860	415	19	2294
Dairy	1455	398	13	1866
Poultry and Eggs	1222	816	6	2044
Vegetables	1557	222	125	1904

Table 13: Relative contribution of SCCPs in the beef and small ruminant meat value chains to reducing foodborne deaths in Ethiopia

Step	SCCP	Anthroponotic	Zoonotic	Chemicals
Farm	Feeding	0	0.0	5
Farm	Vaccination	0	0.0	0
Abattoir	Antemortem Inspection	0	2.0	0
Abattoir	Post-mortem Inspection	0	2.0	0
Abattoir	Carcass Wash	2	1.5	5
Abattoir	Carcass Cold Storage	1	0.5	0
Transport	Transportation Carcass	2	1.0	0
Market/Retail	Storage at Butcher	3	1.5	0
Household	Storage at Home	1	0.5	0
Household	Cooking	1	1.0	0

8.2 Supply Chain Control Points

Participants identified SCCPs in the four selected farm-to-fork chains and then weighted the relative impact of each SCCP on preventing deaths due to three hazard categories: anthroponotic pathogens, zoonotic pathogens and chemicals. They distributed 10 points per hazard category over each identified SCCP in the corresponding food supply chain in a group discussion. The number of preventable deaths by each SCCP was then calculated as the proportion of points assigned to that SCCP, multiplied by the number of attributable deaths per hazard category for each food chain separately, see Table 13 through Table 20.

Table 14: Preventable deaths by SCCPs in the beef and small ruminant meat value chains in Ethiopia

Step	SCCP	Anthroponotic	Zoonotic	Chemicals	Total Deaths
Farm	Feeding	0	0	10	10
Farm	Vaccination	0	0	0	0
Abattoir	Antemortem Inspection	0	83	0	83
Abattoir	Post-mortem Inspection	0	83	0	83
Abattoir	Carcass Wash	372	62	10	444
Abattoir	Carcass Cold Storage	186	21	0	207
Transport	Transportation Carcass	372	42	0	414
Market/Retail	Storage at Butcher	558	62	0	620
Household	Storage at Home	186	21	0	207
Household	Cooking	186	42	0	228

Table 15: Relative contribution of SCCPs in the dairy value chain to reducing foodborne deaths in Ethiopia

Step	SCCP	Anthroponotic	Zoonotic	Chemicals
Farm	Feed & Water	0.0	0.0	5.0
Farm	Pre-milking	2.0	2.0	0.0
Farm	Post-milking	0.5	0.0	0.0
Collector	Quality Check	2.0	2.0	2.5
Collector	Cold transport	0.5	0.5	0.0
Supplier	Cold transport	0.5	0.5	0.0
Processor	Raw Material (milk?)	1.0	1.0	2.5
Processor	Pasteurization	2.0	2.0	0.0
Market/Retail	Cold transport	0.5	1.0	0.0
Household	Storage and handling	1.0	1.0	0.0

Table 16: Preventable deaths by SCCPs in the dairy value chain in Ethiopia

Step	SCCP	Anthroponotic	Zoonotic	Chemicals	Total Deaths
Farm	Feed & Water	0	0	10	10
Farm	Pre-milking	372	83	0	455
Farm	Post-milking	93	0	0	93
Collector	Quality Check	372	83	5	460
Collector	Cold transport	93	21	0	114
Supplier	Cold transport	93	21	0	114
Processor	Raw Material (milk?)	186	42	5	233
Processor	Pasteurization	372	83	0	455
Market/Retail	Cold transport	93	42	0	135
Household	Storage and handling	186	42	0	228

Table 17: Relative contribution of SCCPs in the poultry value chain to reducing foodborne deaths in Ethiopia

Step	SCCP	Anthroponotic	Zoonotic	Chemicals
All	Water Quality	2.0	0.2	2.0
All	Sanitation & Hygiene	3.0	1.0	0.5
Farm	Seed Stock Quality	0.0	2.0	0.5
Farm	Feed Quality	0.0	0.0	3.0
Farm	Feed	0.2	0.2	3.0
Farm	Vaccines & Drugs	0.0	0.0	0.0
Farm	Housing & Bedding	0.2	0.2	0.5
Farm	Equipment	0.2	0.0	0.0
Processing	Slaughter	0.5	1.0	0.0
Processing	Egg Collection	0.5	0.2	0.0
Processing	Egg packaging	0.5	0.0	0.0
Storage	Egg	0.2	0.2	0.0
Storage	Meat	0.2	0.4	0.0
Transport	Temperature	0.0	0.2	0.0
Transport	Vehicles & Containers	0.0	0.2	0.0
Market/Retail	Temperature	0.0	0.2	0.0
Market/Retail	Live birds	0.0	2.0	0.5
Market/Retail	Eggs	0.5	1.0	0.0
Household	Cooking	1.0	0.5	0.0
Household	Cross-contamination	1.0	0.5	0.0

Table 18: Preventable deaths by SCCPs in the poultry value chain in Ethiopia

Step	SCCP	Anthroponotic	Zoonotic	Chemicals	Total Deaths
All	Water Quality	372	8	4	384
All	Sanitation & Hygiene	558	42	1	601
Farm	Seed Stock Quality	0	83	1	84
Farm	Feed Quality	0	0	6	6
Farm	Feed	37	8	6	51
Farm	Vaccines & Drugs	0	0	0	0
Farm	Housing & Bedding	37	8	1	46
Farm	Equipment	37	0	0	37
Processing	Slaughter	93	42	0	135
Processing	Egg Collection	93	8	0	101
Processing	Egg packaging	93	0	0	93
Storage	Egg	37	8	0	45
Storage	Meat	37	17	0	54
Transport	Temperature	0	8	0	8
Transport	Vehicles & Containers	0	8	0	8
Market/Retail	Temperature	0	8	0	8
Market/Retail	Live birds	0	83	1	84
Market/Retail	Eggs	93	42	0	135
Household	Cooking	186	21	0	207
Household	Cross-contamination	186	21	0	207

Table 19: Relative contribution of SCCPs in the vegetable value chain to reducing foodborne deaths in Ethiopia

Step	SCCP	Anthroponotic	Zoonotic	Chemicals
Farm	Agricultural Water	2.0	2.0	10
Harvest	Sanitation	1.0	1.5	0
Harvest	Worker Hygiene	1.5	1.5	0
Transport	Clean vehicles	1.5	1.0	0
Transport	Sanitation	1.5	1.0	0
Market/Retail	Improved infrastructure	1.5	2.0	0
Household	Education	1.0	1.0	0

Table 20: Preventable deaths by SCCPs in the vegetable value chain in Ethiopia

Step	SCCP	Anthroponotic	Zoonotic	Chemicals	Total Deaths
Farm	Agricultural Water	372	83	19	474
Harvest	Sanitation	186	62	0	248
Harvest	Worker Hygiene	279	62	0	341
Transport	Clean vehicles	279	42	0	321
Transport	Sanitation	279	42	0	321
Market/Retail	Improved infrastructure	279	83	0	362
Household	Education	186	42	0	228

References

1. Bahiru, G., A. Bekele, B. Seraw, L. Boulanger, and A. Ali. 2016. Human and animal anthrax in Ethiopia: A retrospective record review 2009-2013. *Ethiopian Veterinary Journal* 20:76–85.
2. Gibb, H. J., A. Barchowsky, D. Bellinger, P. M. Bolger, C. Carrington, A. H. Havelaar, S. Oberoi, Y. Zang, K. O'Leary, and B. Devleesschauwer. 2019. Estimates of the 2015 global and regional disease burden from four foodborne metals – arsenic, cadmium, lead and methylmercury. *Environmental Research* 174:188–194.
3. Hanea, A. M., and G. F. Nane. 2021. An in-depth perspective on the classical model, p. 225–256. *In* A.M. Hanea, G.F. Nane, T. Bedford, and S. French (eds.),. Springer, Cham.
4. Havelaar, A. H., M. D. Kirk, P. R. Torgerson, H. J. Gibb, T. Hald, R. J. Lake, N. Praet, D. C. Bellinger, N. R. de Silva, N. Gargouri, N. Speybroeck, A. Cawthorne, C. Mathers, C. Stein, F. J. Angulo, B. Devleesschauwer, and on behalf of W. H. O. F. D. B. E. R. Group. 2015. World Health Organization Global Estimates and Regional Comparisons of the Burden of Foodborne Disease in 2010. *PLOS Medicine* 12:e1001923.
5. Jeppson, H., H. Hofmann, and D. Cook. 2021. Ggmosaic: Mosaic plots in the 'ggplot2' framework.
6. R Core Team. 2023. R: A language and environment for statistical computing. R Foundation for Statistical Computing, Vienna, Austria.
7. Sapp, A. C., M. P. Amaya, A. H. Havelaar, and G. F. Nane. 2022. Attribution of country level foodborne disease to food group and food types in three African countries: Conclusions from a structured expert judgment study. *PLOS Neglected Tropical Diseases* 16:e0010663.
8. Schloerke, B., D. Cook, J. Larmarange, F. Briatte, M. Marbach, E. Thoen, A. Elberg, and J. Crowley. 2023. GGally: Extension to 'ggplot2'.
9. Venables, W. N., and B. D. Ripley. 2002. Modern applied statistics with S.Fourth edition. Springer, New York.

10. Vos, T., S. S. Lim, C. Abbafati, K. M. Abbas, M. Abbasi, M. Abbasifard, M. Abbasi-Kangevari, H. Abbastabar, F. Abd-Allah, A. Abdelalim, M. Abdollahi, I. Abdollahpour, H. Abolhassani, V. Aboyans, E. M. Abrams, L. G. Abreu, M. R. M. Abrigo, L. J. Abu-Raddad, A. I. Abushouk, A. Acebedo, I. N. Ackerman, M. Adabi, A. A. Adamu, O. M. Adebayo, V. Adekanmbi, J. D. Adelson, O. O. Adetokunboh, D. Adham, M. Afshari, A. Afshin, E. E. Agardh, G. Agarwal, K. M. Agesa, M. Aghaali, S. M. K. Aghamir, A. Agrawal, T. Ahmad, A. Ahmadi, M. Ahmadi, H. Ahmadieh, E. Ahmadpour, T. Y. Akalu, R. O. Akinyemi, T. Akinyemiju, B. Akombi, Z. Al-Aly, K. Alam, N. Alam, S. Alam, T. Alam, T. M. Alanzi, S. B. Albertson, J. E. Alcalde-Rabanal, N. M. Alema, M. Ali, S. Ali, G. Alicandro, M. Alijanzadeh, C. Alinia, V. Alipour, S. M. Aljunid, F. Alla, P. Allebeck, A. Almasi-Hashiani, J. Alonso, R. M. Al-Raddadi, K. A. Altirkawi, N. Alvis-Guzman, N. J. Alvis-Zakzuk, S. Amini, M. Amini-Rarani, A. Aminorroaya, F. Amiri, A. M. L. Amit, D. A. Amugsi, G. G. H. Amul, D. Anderlini, C. L. Andrei, T. Andrei, M. Anjomshoa, F. Ansari, I. Ansari, A. Ansari-Moghaddam, C. A. T. Antonio, C. M. Antony, E. Antriyandarti, D. Anvari, R. Anwer, J. Arabloo, M. Arab-Zozani, A. Y. Aravkin, F. Ariani, J. Ärnlöv, K. K. Aryal, A. Arzani, M. Asadi-Aliabadi, A. A. Asadi-Pooya, B. Asghari, C. Ashbaugh, D. D. Atnafu, S. R. Atre, F. Ausloos, M. Ausloos, B. P. Ayala Quintanilla, G. Ayano, M. A. Ayanore, Y. A. Aynalem, S. Azari, G. Azarian, Z. N. Azene, E. Babaee, A. Badawi, M. Bagherzadeh, M. H. Bakhshaei, A. Bakhtiari, S. Balakrishnan, S. Balalla, S. Balassyano, M. Banach, P. C. Banik, M. S. Bannick, A. B. Bante, A. G. Baraki, M. A. Barboza, S. L. Barker-Collo, C. M. Barthelemy, L. Barua, A. Barzegar, S. Basu, B. T. Baune, M. Bayati, G. Bazmandegan, N. Bedi, E. Beghi, Y. Béjot, A. K. Bello, R. G. Bender, D. A. Bennett, F. B. Bennitt, I. M. Bensenor, C. P. Benziger, K. Berhe, E. Bernabe, G. J. Bertolacci, R. Bhageerathy, N. Bhala, D. Bhandari, P. Bhardwaj, K. Bhattacharyya, Z. A. Bhutta, S. Bibi, M. H. Biehl, B. Bikbov, M. S. Bin Sayeed, A. Biondi, B. M. Birihane, D. Bisanzio, C. Bisignano, R. K. Biswas, S. Bohlouli, M. Bohluli, S. R. R. Bolla, A. Boloor, A. S. Boon-Dooley, G. Borges, A. M. Borzì, R. Bourne, O. J. Brady, M. Brauer, C. Brayne, N. J. K. Breitborde, H. Brenner, P. S. Briant, A. M. Briggs, N. I. Briko, G. B. Britton, D. Bryazka, R. Buchbinder, B. R. Bumgarner, R. Busse, Z. A. Butt, F. L. Caetano dos Santos, L. L. A. Cámera, I. R. Campos-Nonato, J. Car, R. Cárdenas, G. Carreras, J. J. Carrero, F. Carvalho, J. M. Castaldelli-Maia, C. A. Castañeda-Orjuela, G. Castelpietra, C. D. Castle, F. Castro, F. Catalá-López, K. Causey, C. R. Cederroth, K. M. Cercy, E. Cerin, J. S. Chandan, A. R. Chang, F. J. Charlson, V. K. Chattu, S. Chaturvedi, O. Chimed-Ochir, K. L. Chin, D. Y. Cho, H. Christensen, D.-T. Chu, M. T. Chung, F. M. Cicuttini, L. G. Ciobanu, M. Cirillo, E. L. Collins, K. Compton, S. Conti, P. A. Cortesi, V. M. Costa, E. Cousin, R. G. Cowden, B. C. Cowie, E. A. Cromwell, D. H. Cross, C. S. Crowe, J. A. Cruz, M. Cunningham, S. M. A. Dahlawi, G. Damiani, L. Dandona, R. Dandona, A. M. Darwesh, A. Daryani, J. K. Das, R. Das Gupta, J. das Neves, C. A. Dávila-Cervantes, K. Davletov, D. De Leo, F. E. Dean, N. K. DeCleene, A. Deen, L. Degenhardt, R. P. Dellavalle, F. M. Demeke, D. G. Demsie, E. Denova-Gutiérrez, N. D. Dereje, N. Dervenis, R. Desai, A. Desalew, G. A. Dessie, S. D. Dharmaratne, G. P. Dhungana, M. Dianatinasab, D. Diaz, Z. S. Dibaji Forooshani, Z. V. Dingels, M. A. Dirac, S. Djalalinia, H. T. Do, K. Dokova, F. Dorostkar, C. P. Doshi, L. Doshmangir, A. Douiri, M. C. Doxey, T. R. Driscoll, S. J. Dunachie, B. B. Duncan, A. R. Duraes, A. W. Eagan, M. Ebrahimi Kalan, D. Edvardsson, J. R. Ehrlich, N. El Nahas, I. El Sayed, M. El Tantawi, I. Elbarazi, I. Y. Elgendy, H. R. Elhabashy, S. I. El-Jaafary, I. R. Elyazar, M. H. Emamian, S. Emmons-Bell, H. E. Erskine, B. Eshrati, S. Eskandarieh, S. Esmaeilnejad, F. Esmaeilzadeh, A. Esteghamati, K. Estep, A. Etemadi, A. E. Etisso, M. Farahmand, A. Faraj, M. Fareed, R. Faridnia, C. S. e. S. Farinha, A. Farioli, A. Faro, M. Faruque, F. Farzadfar, N. Fattahi, M. Fazlzadeh, V. L. Feigin, R. Feldman, S.-M. Fereshtehnejad, E. Fernandes, A. J. Ferrari, M. L. Ferreira, I. Filip, F. Fischer, J. L. Fisher, R. Fitzgerald, C. Flohr, L. S. Flor, N. A. Foigt, M. O. Folayan, L. M. Force, C. Fornari, M. Foroutan, J. T. Fox, M. Freitas, W. Fu, T. Fukumoto, J. M. Furtado, M. M. Gad, E. Gakidou, N. C. Galles, S. Gallus, A. Gamkrelidze, A. L. Garcia-Basteiro, W. M. Gardner, B. S. Geberemariyam, A. M. Gebrehiwot, K. B. Gebremedhin, A. A. A. A. Gebreslassie, A. Gershberg Hayoon, P. W. Gething, M. Ghadimi, K. Ghadiri, M. Ghafourifard, A. Ghajar, F. Ghamari, A. Ghashghaee, H. Ghiasvand, N. Ghith, A. Gholamian, S. A. Gilani, P. S. Gill, M. Gitimoghaddam, G. Giussani, S. Goli, R. S. Gomez, S. V. Gopalani, G. Gorini, T. M. Gorman, H. C. Gottlich, H. Goudarzi, A. C. Goulart, B. N. G. Goulart, A. Grada, M. Grivna, G. Grosso, M. I. M. Gubari, H. C. Gugnani, A. L. S. Guimaraes, R. A. Guimarães, R. A. Guled, G. Guo, Y. Guo, R. Gupta, J. A. Haagsma, B. Haddock, N. Hafezi-Nejad, A. Hafiz, H. Hagins, L. M. Haile, B. J.

Hall, I. Halvaei, R. R. Hamadeh, K. Hamagharib Abdullah, E. B. Hamilton, C. Han, H. Han, G. J. Hankey, J. M. Haro, J. D. Harvey, A. I. Hasaballah, A. Hasanzadeh, M. Hashemian, S. Hassanipour, H. Hassankhani, R. J. Havmoeller, R. J. Hay, S. I. Hay, K. Hayat, B. Heidari, G. Heidari, R. Heidari-Soureshjani, D. Hendrie, H. J. Henrikson, N. J. Henry, C. Herteliu, F. Heydarpour, T. R. Hird, H. W. Hoek, M. K. Hole, R. Holla, P. Hoogar, H. D. Hosgood, M. Hosseinzadeh, M. Hostiuc, S. Hostiuc, M. Househ, D. G. Hoy, M. Hsairi, V. C. Hsieh, G. Hu, T. M. Huda, F. N. Hugo, C. K. Huynh, B.-F. Hwang, V. C. Iannucci, S. E. Ibitoye, K. S. Ikuta, O. S. Ilesanmi, I. M. Ilic, M. D. Ilic, L. R. Inbaraj, H. Ippolito, S. S. N. Irvani, M. M. Islam, M. Islam, S. M. S. Islam, F. Islami, H. Iso, R. Q. Ivers, C. C. D. Iwu, I. O. Iyamu, J. Jaafari, K. H. Jacobsen, F. Jadidi-Niaragh, H. Jafari, M. Jafarinia, D. Jahagirdar, M. A. Jahani, N. Jahanmehr, M. Jakovljevic, A. Jalali, F. Jalilian, S. L. James, H. Janjani, M. D. Janodia, A. U. Jayatilleke, P. Jeemon, E. Jenabi, R. P. Jha, V. Jha, J. S. Ji, P. Jia, O. John, Y. O. John-Akinola, C. O. Johnson, S. C. Johnson, J. B. Jonas, T. Joo, A. Joshi, J. J. Jozwiak, M. Jürisson, A. Kabir, Z. Kabir, H. Kalani, R. Kalani, L. R. Kalankesh, R. Kalhor, Z. Kamiab, T. Kanchan, B. Karami Matin, A. Karch, M. A. Karim, S. E. Karimi, G. M. Kassa, N. J. Kassebaum, S. V. Katikireddi, N. Kawakami, G. A. Kayode, S. H. Keddie, C. Keller, M. Kereselidze, M. A. Khafaie, N. Khalid, M. Khan, K. Khatab, M. M. Khater, M. N. Khatib, M. Khayamzadeh, M. T. Khodayari, R. Khundkar, N. Kianipour, C. Kieling, D. Kim, Y.-E. Kim, Y. J. Kim, R. W. Kimokoti, A. Kisa, S. Kisa, K. Kissimova-Skarbek, M. Kivimäki, C. J. Kneib, A. K. S. Knudsen, J. M. Kocarnik, T. Kolola, J. A. Kopec, S. Kosen, P. A. Koul, A. Koyanagi, M. A. Kravchenko, K. Krishan, K. J. Krohn, B. Kuate Defo, B. Kucuk Bicer, G. A. Kumar, M. Kumar, P. Kumar, V. Kumar, G. Kumaresh, O. P. Kurmi, D. Kusuma, H. H. Kyu, C. La Vecchia, B. Lacey, D. K. Lal, R. Lalloo, J. O. Lam, F. H. Lami, I. Landires, J. J. Lang, V. C. Lansingh, S. L. Larson, A. O. Larsson, S. Lasrado, Z. S. Lassi, K. M.-M. Lau, P. M. Lavados, J. V. Lazarus, J. R. Ledesma, P. H. Lee, S. W. H. Lee, K. E. LeGrand, J. Leigh, M. Leonardi, H. Lescinsky, J. Leung, M. Levi, S. Lewington, S. Li, L.-L. Lim, C. Lin, R.-T. Lin, C. Linehan, S. Linn, H.-C. Liu, S. Liu, Z. Liu, K. J. Looker, A. D. Lopez, P. D. Lopukhov, S. Lorkowski, P. A. Lotufo, T. C. D. Lucas, A. Lugo, R. Lunevicius, R. A. Lyons, J. Ma, J. H. MacLachlan, E. R. Maddison, R. Maddison, F. Madotto, P. W. Mahasha, H. T. Mai, A. Majeed, V. Maled, S. Maleki, R. Malekzadeh, D. C. Malta, A. A. Mamun, A. Manafi, N. Manafi, H. Manguerra, B. Mansouri, M. A. Mansournia, A. M. Mantilla Herrera, J. C. Maravilla, A. Marks, F. R. Martins-Melo, I. Martopullo, S. Z. Masoumi, J. Massano, B. B. Massenburg, M. R. Mathur, P. K. Maulik, C. McAlinden, J. J. McGrath, M. McKee, M. M. Mehndiratta, F. Mehri, K. M. Mehta, W. B. Meitei, P. T. N. Memiah, W. Mendoza, R. G. Menezes, E. W. Mengesha, M. B. Mengesha, A. Mereke, A. Meretoja, T. J. Meretoja, T. Mestrovic, B. Miazgowski, T. Miazgowski, I. M. Michalek, K. M. Mihretie, T. R. Miller, E. J. Mills, A. Mirica, E. M. Mirrakhimov, H. Mirzaei, M. Mirzaei, M. Mirzaei-Alavijeh, A. T. Misganaw, P. Mithra, B. Moazen, M. Moghadaszadeh, E. Mohamadi, D. K. Mohammad, Y. Mohammad, N. Mohammad Gholi Mezerji, A. Mohammadian-Hafshejani, N. Mohammadifard, R. Mohammadpourhodki, S. Mohammed, A. H. Mokdad, M. Molokhia, N. C. Momen, L. Monasta, S. Mondello, M. D. Mooney, M. Moosazadeh, G. Moradi, M. Moradi, M. Moradi-Lakeh, R. Moradzadeh, P. Moraga, L. Morales, L. Morawska, I. Moreno Velásquez, J. Morgado-da-Costa, S. D. Morrison, J. F. Mosser, S. Mouodi, S. M. Mousavi, A. Mousavi Khaneghah, U. O. Mueller, S. B. Munro, M. K. Muriithi, K. I. Musa, S. Muthupandian, M. Naderi, A. J. Nagarajan, G. Nagel, B. Naghshtabrizi, S. Nair, A. K. Nandi, V. Nangia, J. R. Nansseu, V. C. Nayak, J. Nazari, I. Negoi, R. I. Negoi, H. B. N. Netsere, J. W. Ngunjiri, C. T. Nguyen, J. Nguyen, M. Nguyen, M. Nguyen, E. Nichols, D. Nigatu, Y. T. Nigatu, R. Nikbakhsh, M. R. Nixon, C. A. Nnaji, S. Nomura, B. Norrving, J. J. Noubiap, C. Nowak, V. Nunez-Samudio, A. Oţoiu, B. Oancea, C. M. Odell, F. A. Ogbo, I.-H. Oh, E. W. Okunga, M. Oladnabi, A. T. Olagunju, B. O. Olusanya, J. O. Olusanya, M. M. Oluwasanu, A. Omar Bali, M. O. Omer, K. L. Ong, O. E. Onwujekwe, A. U. Orji, H. M. Orpana, A. Ortiz, S. M. Ostroff, N. Otstavnov, S. S. Otstavnov, S. Øverland, M. O. Owolabi, M. P a, J. R. Padubidri, A. P. Pakhare, R. Palladino, A. Pana, S. Panda-Jonas, A. Pandey, E.-K. Park, P. G. K. Parmar, D. K. Pasupula, S. K. Patel, A. J. Paternina-Caicedo, A. Pathak, M. Pathak, S. B. Patten, G. C. Patton, D. Paudel, H. Pazoki Toroudi, A. E. Peden, A. Pennini, V. C. F. Pepito, E. K. Peprah, A. Pereira, D. M. Pereira, N. Perico, H. Q. Pham, M. R. Phillips, D. M. Pigott, T. Pilgrim, T. M. Pilz, M. Pirsaheb, O. Plana-Ripoll, D. Plass, K. N. Pokhrel, R. V. Polibin, S. Polinder, K. R. Polkinghorne, M. J. Postma, H. Pourjafar, F. Pourmalek, R. Pourmirza Kalhori, A. Pourshams, A. Poznańska, S. I. Prada, V. Prakash, D.

R. A. Pribadi, E. Pupillo, Z. Quazi Syed, M. Rabiee, N. Rabiee, A. Radfar, A. Rafiee, A. Rafiei, A. Raggi, A. Rahimi-Movaghar, M. A. Rahman, A. Rajabpour-Sanati, F. Rajati, K. Ramezanzadeh, C. L. Ranabhat, P. C. Rao, S. J. Rao, D. Rasella, P. Rastogi, P. Rathi, D. L. Rawaf, S. Rawaf, L. Rawal, C. Razo, S. B. Redford, R. C. Reiner, N. Reinig, M. B. Reitsma, G. Remuzzi, V. Renjith, A. M. N. Renzaho, S. Resnikoff, N. Rezaei, M. sadegh Rezai, A. Rezapour, P.-A. Rhinehart, S. M. Riahi, A. L. P. Ribeiro, D. C. Ribeiro, D. Ribeiro, J. Rickard, N. L. S. Roberts, S. Roberts, S. R. Robinson, L. Roever, S. Rolfe, L. Ronfani, G. Roshandel, G. A. Roth, E. Rubagotti, S. F. Rumisha, S. Sabour, P. S. Sachdev, B. Saddik, E. Sadeghi, M. Sadeghi, S. Saeidi, S. Safi, S. Safiri, R. Sagar, A. Sahebkar, M. A. Sahraian, S. M. Sajadi, M. R. Salahshoor, P. Salamati, S. Salehi Zahabi, H. Salem, M. R. R. Salem, H. Salimzadeh, J. A. Salomon, I. Salz, Z. Samad, A. M. Samy, J. Sanabria, D. F. Santomauro, I. S. Santos, J. V. Santos, M. M. Santric-Milicevic, S. Y. I. Saraswathy, R. Sarmiento-Suárez, N. Sarrafzadegan, B. Sartorius, A. Sarveazad, B. Sathian, T. Sathish, D. Sattin, A. N. Sbarra, L. E. Schaeffer, S. Schiavolin, M. I. Schmidt, A. E. Schutte, D. C. Schwebel, F. Schwendicke, A. M. Senbeta, S. Senthilkumaran, S. G. Sepanlou, K. A. Shackelford, J. Shadid, S. Shahabi, A. A. Shaheen, M. A. Shaikh, A. S. Shalash, M. Shams-Beyranvand, M. Shamsizadeh, M. Shannawaz, K. Sharafi, F. Sharara, B. S. Sheena, A. Sheikhtaheri, R. S. Shetty, K. Shibuya, W. S. Shiferaw, M. Shigematsu, J. I. Shin, R. Shiri, R. Shirkoohi, M. G. Shrime, K. Shuval, S. Siabani, I. D. Sigfusdottir, R. Sigurvinsdottir, J. P. Silva, K. E. Simpson, A. Singh, J. A. Singh, E. Skiadaresi, S. T. S. Skou, V. Y. Skryabin, E. Sobngwi, A. Sokhan, S. Soltani, R. J. D. Sorensen, J. B. Soriano, M. B. Sorrie, I. N. Soyiri, C. T. Sreeramareddy, J. D. Stanaway, B. A. Stark, S. C. Ştefan, C. Stein, C. Steiner, T. J. Steiner, M. A. Stokes, L. J. Stovner, J. L. Stubbs, A. Sudaryanto, M. B. Sufiyan, G. Sulo, I. Sultan, B. L. Sykes, D. O. Sylte, M. Szócska, R. Tabarés-Seisdedos, K. M. Tabb, S. K. Tadakamadla, A. Taherkhani, M. Tajdini, K. Takahashi, N. Taveira, W. L. Teagle, H. Teame, A. Tehrani-Banihashemi, B. F. Teklehaimanot, S. Terrason, Z. T. Tessema, K. R. Thankappan, A. M. Thomson, H. R. Tohidinik, M. Tonelli, R. Topor-Madry, A. E. Torre, M. Touvier, M. R. R. Tovani-Palone, B. X. Tran, R. Travillian, C. E. Troeger, T. C. Truelsen, A. C. Tsai, A. Tsatsakis, L. Tudor Car, S. Tyrovolas, R. Uddin, S. Ullah, E. A. Undurraga, B. Unnikrishnan, M. Vacante, A. Vakilian, P. R. Valdez, S. Varughese, T. J. Vasankari, Y. Vasseghian, N. Venketasubramanian, F. S. Violante, V. Vlassov, S. E. Vollset, A. Vongpradith, A. Vukovic, R. Vukovic, Y. Waheed, M. K. Walters, J. Wang, Y. Wang, Y.-P. Wang, J. L. Ward, A. Watson, J. Wei, R. G. Weintraub, D. J. Weiss, J. Weiss, R. Westerman, J. L. Whisnant, H. A. Whiteford, T. Wiangkham, K. E. Wiens, T. Wijeratne, L. B. Wilner, S. Wilson, B. Wojtyniak, C. D. A. Wolfe, E. E. Wool, A.-M. Wu, S. Wulf Hanson, H. Y. Wunrow, G. Xu, R. Xu, S. Yadgir, S. H. Yahyazadeh Jabbari, K. Yamagishi, M. Yaminfirooz, Y. Yano, S. Yaya, V. Yazdi-Feyzabadi, J. A. Yearwood, T. Y. Yeheyis, Y. G. Yeshitila, P. Yip, N. Yonemoto, S.-J. Yoon, J. Yoosefi Lebni, M. Z. Younis, T. P. Younker, Z. Yousefi, M. Yousefifard, T. Yousefinezhadi, A. Y. Yousuf, C. Yu, H. Yusefzadeh, T. Zahirian Moghadam, L. Zaki, S. B. Zaman, M. Zamani, M. Zamanian, H. Zandian, A. Zangeneh, M. S. Zastrozhin, K. A. Zewdie, Y. Zhang, Z.-J. Zhang, J. T. Zhao, Y. Zhao, P. Zheng, M. Zhou, A. Ziapour, S. R. M. Zimsen, M. Naghavi, and C. J. L. Murray. 2020. Global burden of 369 diseases and injuries in 204 countries and territories, 19902019: a systematic analysis for the Global Burden of Disease Study 2019. *The Lancet* 396:1204–1222.

11. Vose, D. 2008. Risk analysis, a quantitative guide Third edition. John Wiley & Sons, Chicester, England.

12. Vose, D. 2008. Risk analysis, a quantitative guide Third edition. John Wiley & Sons, Chicester, England.

13. Wickham, H. 2016. ggplot2: Elegant graphics for data analysis. Springer-Verlag New York.

14. Wickham, H., R. François, L. Henry, K. Müller, and D. Vaughan. 2023. Dplyr: A grammar of data manipulation.

Session information

Show the code

```
─ Session info ───────────────────────────────────────────────────────────────
 setting  value
 version  R version 4.4.2 (2024-10-31)
 os       macOS Sequoia 15.3.1
 system   x86_64, darwin20
 ui       X11
 language (EN)
 collate  en_US.UTF-8
 ctype    en_US.UTF-8
 tz       America/New_York
 date     2025-03-06
 pandoc   3.2 @
/Applications/RStudio.app/Contents/Resources/app/quarto/bin/tools/x86_64/
(via rmarkdown)

─ Packages ───────────────────────────────────────────────────────────────────
 package      * version date (UTC) lib source
 brant        * 0.3-0   2020-09-22 [1] CRAN (R 4.4.0)
 carData      * 3.0-5   2022-01-06 [1] CRAN (R 4.4.0)
 dplyr        * 1.1.4   2023-11-17 [1] CRAN (R 4.4.0)
 effects      * 4.2-2   2022-07-13 [1] CRAN (R 4.4.0)
 forcats      * 1.0.0   2023-01-29 [1] CRAN (R 4.4.0)
 GGally       * 2.2.1   2024-02-14 [1] CRAN (R 4.4.0)
 ggmosaic     * 0.3.3   2021-02-23 [1] CRAN (R 4.4.0)
 ggplot2      * 3.5.1   2024-04-23 [1] CRAN (R 4.4.0)
 ggpubr       * 0.6.0   2023-02-10 [1] CRAN (R 4.4.0)
 ggtext       * 0.1.2   2022-09-16 [1] CRAN (R 4.4.0)
 gt           * 0.11.1  2024-10-04 [1] CRAN (R 4.4.1)
 Hmisc        * 5.1-3   2024-05-28 [1] CRAN (R 4.4.0)
 janitor      * 2.2.0   2023-02-02 [1] CRAN (R 4.4.0)
 knitr        * 1.48    2024-07-07 [1] CRAN (R 4.4.0)
 lubridate    * 1.9.3   2023-09-27 [1] CRAN (R 4.4.0)
 MASS         * 7.3-61  2024-06-13 [1] CRAN (R 4.4.2)
 mc2d         * 0.2.1   2024-06-05 [1] CRAN (R 4.4.0)
 mvtnorm      * 1.3-1   2024-09-03 [1] CRAN (R 4.4.1)
 purrr        * 1.0.2   2023-08-10 [1] CRAN (R 4.4.0)
 purrrlyr     * 0.0.8   2022-03-29 [1] CRAN (R 4.4.0)
 RColorBrewer * 1.1-3   2022-04-03 [1] CRAN (R 4.4.0)
 readr        * 2.1.5   2024-01-10 [1] CRAN (R 4.4.0)
 readxl       * 1.4.3   2023-07-06 [1] CRAN (R 4.4.0)
 reshape2     * 1.4.4   2020-04-09 [1] CRAN (R 4.4.0)
 stringr      * 1.5.1   2023-11-14 [1] CRAN (R 4.4.0)
 tibble       * 3.2.1   2023-03-20 [1] CRAN (R 4.4.0)
 tidyr        * 1.3.1   2024-01-24 [1] CRAN (R 4.4.0)
 tidyverse    * 2.0.0   2023-02-22 [1] CRAN (R 4.4.0)

 [1] /Library/Frameworks/R.framework/Versions/4.4-x86_64/Resources/library
```

Appendix A. Expert rationale in Round 1

Hazard	Rationale
Aflatoxin	Milk and grain takes the highest percentage of aflatoxin source. Inadequate harvesting and storage techniques allow for the growth of aflatoxin-producing fungus. Oil seeds and byproducts are also common sources.
Aflatoxin B1	The study on occurrence of hazards in aflatoxin in Ethiopia aims to identify the priority hazards, reveal specific sources and contribute the choice of interventions. The aflatoxin B1 (AFB1) prioritized as hazard foodborne disease especially in the following food items: 1. Found contamination of maize with aflatoxin B1 (AFB1) in Southern Ethiopia with concentration of 22.72μg/kg. 2. According to a report by USAID in 2011, aflatoxin B1 was detected in four major crops of Ethiopia: barley, sorghum, Teff and wheat 3. staple cereals 4. Dairy and Dairy products 5. beef and ruminant meat
Aflatoxin B1	Most people in Ethiopia feed their animals which can be a source for Aflatoxin and grain we consume could be potential sources of the problem
Aflatoxin B1	Aflatoxin B1 can be found in many food commodities but the risk level in grains and beans can be the highest since most of the staple food in Ethiopia is grain based. Next to grains and beans, dairy, beef and poultry could be at high risk of carrying aflatoxin B1 mainly from feed sources.
Aflatoxin B1	Consumption of peanut butter and other Cereal grain crops products which are contaminated with fungal and mold.
Aflatoxin B1	Commonly found in chili powder which commonly used in Ethiopian foods
Aflatoxin B1	major source is peanuts and rice stored in warm and humid conditions, and it the major source for Aflatoxin M1 from animal fed contaminated by Aflatoxin B1
Aflatoxin B1	This could be dangerous in animal feeds like grains and bean or nuts that could also be affect in Dairy and fish.
Aflatoxin B1	it mostly common in lack of good storage condition of cereal and nuts and lack of Good Agricultural practices, and also lack of food safety knowledge. This all are very common in our country
Aflatoxin B1	Currently Aflatoxin B1 is found in different agricultural product in Ethiopia, especially in dairy products
Aflatoxin B1	This is because of the nature of the food, when grains and beans are stored in moist areas the fungus responsible for the production of the toxin can easily grow and contaminate the food. Regarding spices like berbere (red pepper), malpractices done by the seelers (adding water to it to get economic advantages favours the release of the toxins). In addition, consumption of these products are high in Ethiopia.

Hazard	Rationale
Aflatoxin B1	Recent studies in Ethiopia revealed that Aflatoxin is an emerging food safety problem. These studied pointed out that dairy (milk), nuts and grains are heavily contaminated by Aflatoxin B. These problems are associated with improper storage of animal feeds and grains.
Arsenic	Arsenic poisoning constitutes a major threat to humans, causing various health problems; as a World Africa is third exposed continent to Arsenic food poisoning heavy metals mainly; Grain and beans, Vegetables, and Shellfish (Oysters)
Arsenic	Arsenic poisoning occurs when you ingest or consume high levels of arsenic. It shares features of other heavy metal poisonings, including mercury and lead. Drinking contaminated water causes most cases of arsenic poisoning.
Arsenic	Because of the water sources we dink especially in low land areas
Arsenic	Vegetables, and fruits and nuts are the two high risk food groups in terms of Arsenic exposure. Thirdly, poultry meat, and grains and beans have equal risk.
Arsenic	Consumption of contaminated fruit and vegetable product which are grown by contaminated water discharged from industries and marine/fish product harvested from contaminated river or lake
Arsenic	With out withdrawal period medication of Arsenic could lead to dose of consumers.
Arsenic	mostly common in vegetable contaminated by river water from industries in the city
Arsenic	Arsenic is usually in the environment like soil, water and air. Its amount can also be high in areas where arsenic containing pesticides are used and as a result it can contaminate those food like grains, beans, vegetables. It can also be transmitted to animal origin foods like poultry, beef etc. Consumption of these foods are also relatively high in Ethiopia.
Arsenic	Some studies in Ethiopia revealed that Arsenic (AS) is found in an elevated level in some lakes and surface water bodies, which potentially harms the health of the people through ingestion of contaminated irrigated crops (vegetables and fruits) and water. Besides, literature evidences showed that Arsenic is found in ground water, in which irrigated crops (vegetables, seafoods, fishes, foods prepared by ground water) can be easily contaminated by Arsenic.
Campylobacter	*Campylobacter* infection is mostly by eating raw or undercooked poultry and meat but also other foods like seafood. .
Campylobacter spp.	Dairy, beef and poultry mainly attribute to the foodborne diseases in Ethiopia due to the people in the country consumption habit for the mentioned food items contribute high rate.

Hazard	Rationale
Campylobacter spp.	The majority of isolates obtained from human samples had co-occurrence with isolates from cattle, poultry or water samples from household. The use of stored water, the practice of indoor and outdoor manure collecting, and animal very common in Ethiopia.
Campylobacter spp.	Dairy, beef and poultry products are the three most vulnerable food sources for *Campylobacter* spp. contamination. The predominant raw meat eating habit may contribute for consumers exposure to the bacteria. Although poultry products can be contaminated by *Campylobacter* spp. more than dairy and beef, the eating/cooking/ practice mostly involves high temperature long time process leading to reduced risk of transferring the bacteria to consumers. However, cross contamination may commonly occur in kitchen environment.
Campylobacter spp.	We found this pathogen from poultry meat in our laboratory
Campylobacter spp.	the most common known source for *Campylobacter* is poultry, but due to their diverse nature reservoir, *Campylobacter* can also transmitted in water. Other known sources of *Campylobacter* infections include food products (raw & undercook foods), such as unpasteurized milk and contaminated fresh produce (contaminated water, working area/kitchen and hands) and Ready to eat foods.
Campylobacter spp.	*Campylobacter* spp are most common dairy products and are also common in untreated drinking water. In addition, it is also common in unclean surface
Campylobacter spp.	*Campylobacter* is usually prevalent in dairy products and poultry meat. Because these food environments is the ideal place for the growth of the microorganism. Untreated water may also be contaminated with campy. Therefore, since these foods are consumed in the country, it may lead to food borne illness.
Campylobacter spp.	Research findings in Ethiopia revealed that poultry meat and egg are common sources of Campylobacter, and higher scores is given to these food types.
EPEC	Affect children, esp. young infants and people living under bad hygienic conditions. They are an important cause of child mortality in developing countries. Common foods like beef and poultry are important sources.
ETEC	Mostly the problem of developing countries and cause of diarrhea in small children due to contaminated infected food handlers
Enteropathogenic *Escherichia coli*	Enteropathogenic *Escherichia coli* (EPEC) is at type of *E. coli* bacteria that can make people sick with diarrhea. It is spread in food and poop which can get into food when people cannot wash their hands properly. Dairy product, beef and some small ruminant meat are the main primary source of EPEC in Ethiopia.
Enteropathogenic *Escherichia coli*	Most people in Ethiopia are Leaving and having unhygienic condition
Enteropathogenic *Escherichia coli*	Beef, poultry and dairy products are the three most vulnerable food groups to transfer Enteropathogenic *E. coli*.

Hazard	Rationale
Enteropathogenic *Escherichia coli*	consumption of contaminated fruit and vegetable product which are grown by contaminated effluent from domestic and also preparation of food with poor personal hygiene
Enteropathogenic *Escherichia coli*	Commonly isolated in our laboratory from raw meat and milk
Enteropathogenic *Escherichia coli*	a bacterial foodborne pathogen and is a major cause of infantile diarrhea worldwide that is associated with a high rate of morbidity and mortality.
Enteropathogenic *Escherichia coli*	it is mostly common in contaminated food and less hygienic areas
Enteropathogenic *Escherichia coli*	*E. coli* is a common bacterial pathogen in food with where there is poor hygiene and sanitation
Enteropathogenic *Escherichia coli*	Both enteropathogenic and Enterotoxigenic *E. coli* can contaminate animal and plant origin foods. Hence since beef, poultry meat, dairy products, vegetables and untreated water are moderately consumed in the country, it can be considered as a potential sources for the transmission of the bacteria.
Enteropathogenic *Escherichia coli*	EPEC commonly acquired in contaminated beef, vegetables and dairy
Enterotoxigenic *Escherichia coli*	Diary product, and beef mainly attribute to the foodborne diseases in Ethiopia due to the people in the country consume at high rate. Especially, Cattle rare region in Ethiopia use Milk and beef which are unheated and uncooked respectively. So, the foodborne around that area prioritized or associated with livelihood.
Enterotoxigenic *Escherichia coli*	not significantly found in meat by, it may happen some times.
Enterotoxigenic *Escherichia coli*	ETEC is transmitted by food or water contaminated with animal or human feces
Enterotoxigenic *Escherichia coli*	High risk of Enterotoxigenic *E. coli* is related with beef, dairy and vegetables in descending order.
Enterotoxigenic *Escherichia coli*	is a major cause of diarrhea in children & travelers in lower income countries specially among children. Its transmitted by food and or water contaminated with animal or human feces.
Enterotoxigenic *Escherichia coli*	ETEC are commonly acquired in contaminated beef, vegetables and dairy. Dairy calves can serve as a source of ETEC infection to children
Mycobacterium bovis	Most commonly, infection with *M. bovis* is due to eating or drinking contaminated, unpasteurized dairy products.

Hazard	Rationale
Mycobacterium bovis	Most causes of this disease is Diary. It is estimated that *M. bovis* causes 10-15% of human cases of tuberculosis in countries. This indicated that tuberculosis in both humans and animals is endemic in Ethiopia.
Mycobacterium bovis	Hazards of consumption of unpasteurized milk and milk products in high-in Ethiopia
Mycobacterium bovis	TB is common case in cattle. It can easily be transmitted to human through consumption of raw milk and beef
Mycobacterium bovis	Dairy products are considered as a sole source of *M. bovis*, and hence 100 percent is given.
Non-typhoidal *Salmonella enterica*	Beef, eggs and poultry meat are the main source of food for this disease. Also, dairy, small ruminant meat and other grain, vegetables contribute mild sources
Non-typhoidal *Salmonella enterica*	Most people in Ethiopia Leaving and having unhygienic condition
Non-typhoidal *Salmonella enterica*	Eggs, poultry meat ad beef are the three descending risky food items with respect to contamination by Non-typhoidal *Salmonella enterica*.
Non-typhoidal *Salmonella enterica*	Commonly isolated in our laboratory from raw meat and milk
Non-typhoidal *Salmonella enterica*	results from contaminated animal-derived food products like beef, small ruminant meat, poultry, eggs and dairy products even other ready to eat foods contaminated in kitchen and food processing areas.
Non-typhoidal *Salmonella enterica*	poultry and dairy products are the most vehicle for *Salmonella* spp. This foods are also common in most population are my reason for attribution
Non-typhoidal *Salmonella enterica*	Non-typhoidal *Salmonella* is a common bacterial pathogen in food with where there is poor hygiene and sanitation, especially in dairy products
Non-typhoidal *Salmonella enterica*	This bacteria can easily contaminate both animal and plat source foods (Beef, Dairy, Poultry meat, egg and vegetables). Different studies in the country revealed the contamination of these foods with this bacteria. People are consuming these foods but the regulation of these products in the country is weak.
Non-typhoidal *Salmonella enterica*	High scores were given to poultry meat, egg, beef and dairy products, which are common animal source foods to be contaminated by non-typhoidal salmonellosis. However, vegetables contaminated by animal manure can also be serve as a source of infection. Poultry products, meat and egg serves as a major source of salmonellosis
Norovirus	Rotavirus is a common cause of severe gastro-enteritis in children in Ethiopia. That is why it is occurred in listed food items mainly.

Hazard	Rationale
Norovirus	Norovirus infection is mostly due to eating contaminated food and/or vegetables.
Norovirus	Norovirus in Ethiopian context is mostly associated with vegetables, and fruits and nuts.
Norovirus	Limited/no studies found on level of contamination of Norovirus among Ethiopian food types. However, European Food Safety Authority (EFSA) identified norovirus (NoV) as the major foodborne viruses of public health significance. Norovirus can be transmitted through ingestion of contaminated vegetables and fruits, especially in ready to eat food items due to unhygienic food preparations.
Rotavirus	Rotavirus is the most common cause of severe diarrheal disease, which is associated with 128, 500 deaths in Ethiopia and also, Ethiopia is among the five countries with the highest rotavirus burden accounting for six percent of the global rotavirus deaths. The half cause food item is Shellfish and vegetables.
Rotavirus	Eating of contaminated food or drinking water, not properly clean utensils using for food preparation.
Rotavirus	is common in waste water contaminating food items plus untreated drinking water.
Salmonella Typhi	*S*. typhi can cause an infection of typhoid fever and is a life threating food born illness. Mostly, unsafe water and food and poor sanitation are source of the infection.
Salmonella Typhi	Even though we can get salmonella infection from a variety of food, there is high consumption of beef and poultry in our case. so that there is high possibility of cross contamination. The cases can be shown on children, immunocompromised and aged people.
Salmonella Typhi	In Ethiopia showed typhoidal *Salmonella* (*S*. typhi) accounted for 42.1% of the total isolates of *Salmonella* species reported from 1974 to 2006 years indicating typhoid fever is endemic in Ethiopia.
Salmonella Typhi	*Salmonella* Typhi common in row meat
Salmonella Typhi	Drivers and hazards of consumption of unpasteurized milk and milk products in high-in Ethiopia
Salmonella Typhi	Beef is primarily high risk food group for the transfer of *Salmonella* Typhi followed by dairy and vegetable products at equal risk level.
Salmonella Typhi	Eating of raw meat and use of non separate cooking material for raw and cooked foods in the kitchen
Salmonella Typhi	sometimes these strains are isolated in our laboratory from raw meat and milk

Hazard	Rationale
Salmonella Typhi	its more common in food that comes from animals like eggs, beef and poultry. Soil and water can contaminate fruits and vegetables.
Salmonella Typhi	*Salmonella* Typhi is a common bacterial pathogen in food with where there is poor hygiene and sanitation, especially in dairy products
Salmonella Typhi	The rational or justification given for Non-typhoidal *Salmonella* also works here
Salmonella Typhi	High score is only given to vegetables and fruits as major sources of *S*. Typhi.
Shigella	Foods that are consumed raw are more likely to be contaminated with *Shigella* germs. *Shigella* germs can also get on fruits and vegetables if the fields where they grow are contaminated with poop containing the germ.
Shigella spp.	Even though *Shigella* spp. caused by vegetables, Grains and Shellfish; Annual Disease Burden Caused by Shigella spp. is low rank
Shigella spp.	Most people in Ethiopia Leaving and having unhygienic condition
Shigella spp.	In Ethiopian context, fruits and nuts, vegetables and beef are the three high risk food commodities for the transfer of *Shigella* spp. in descending order.
Shigella spp.	Eating contaminated fruit and vegetable and not properly washed the product and using contaminated utensils in the preparation of food in the kitchen
Shigella spp.	sometimes these strains are isolated in our laboratory from raw meat and milk
Shigella spp.	is common in raw vegetables and easily contaminated high moisture containing food
Shigella spp.	*Shigella* is usually contaminating vegetables and fruits (these food products are usually produced with poor sanitation and regulatory mechanism is also very weak in the country). To some extent, it can also be found in dairy foods. Unhygienic drinking water is also a medium for the growth of shigella. Since consumption of these food products is not insignificant, it can impose food borne illness to the community.
Shigella spp.	*Shigella* is transmitted through fecal-oral route, in which contaminated vegetables, fruits, beef and fishes are major vehicles for the transmission of shigellosis
Vibrio cholerae	Cholera is most likely to occur and spread in places with inadequate water treatment, poor sanitation, and inadequate hygiene, such as in our rural case. Source of contamination is usually the feces of an infected person that contaminates water or food.
Vibrio cholerae	From food borne disease caused by vegetables *Vibrio cholerae* Annual disease cause is similar to *Shigella* spp. at low rank.

Hazard	Rationale
Vibrio cholerae	most people in Ethiopia having unhygienic condition and lack of potable water sources
Vibrio cholerae	In relation with *Vibrio cholerae*, fruits and nuts, and vegetables eaten raw serves as a major vehicle.
Vibrio cholerae	Consumption of contaminated fruit and vegetable which grow at bank of river and drinking contaminated water and also poor personal hygiene during food preparation
Vibrio cholerae	uncooked vegetables and untreated drinking water are the leading cause of *V. cholerae* and are common in our case. Also we isolated mostly commonly from this samples during epidemics
Vibrio cholerae	*Vibrio cholera* is usually affecting aquatic foods like sea foods which is rarely consumed in Ethiopia. But this bacteria can also contaminate vegetables and fruits which can be consumed by our population. In addition drinking unhygienic water is also prevalent in Ethiopia, which can be a possible way of vibrio cholera transmission.
Vibrio cholerae	High score is given to Vegetables and Fruits. Associated with unhygienic handling practices, *V. cholera* is one of the major contaminant of vegetables and fruits in Ethiopia.
